# *SARS-CoV-2* infection causes periodontal fibrotic pathogenesis through deregulating mitochondrial beta-oxidation

**DOI:** 10.1038/s41420-023-01474-2

**Published:** 2023-05-26

**Authors:** Yan Gao, Wai Ling Kok, Vikram Sharma, Charlotte Sara Illsley, Sally Hanks, Christopher Tredwin, Bing Hu

**Affiliations:** 1 Stem Cells & Regenerative Medicine Laboratory, Peninsula Dental School, Faculty of Health, University of Plymouth, 16 Research Way, Plymouth, PL6 8BU UK; 2 School of Biomedical Sciences, Faculty of Health, University of Plymouth, 16 Research Way, Plymouth, PL6 8BU UK

**Keywords:** Mechanisms of disease, Stress signalling

## Abstract

The global high prevalence of COVID-19 is a major challenge for health professionals and patients. SARS-CoV-2 virus has four structural protein components: the spike protein, envelope protein, membrane protein, and nucleocapsid protein. The SARS-CoV-2 virus mutates predominantly in the spike proteins, whilst the other key viral components usually remain stable. Essentially the pathological functions of the SARS-CoV-2 virus on different cell types are still largely unknown. Previous studies have shown that the human oral cavity can potentially act as reservoir of the SARS-CoV-2 virus. However, the consequence of SARS-CoV-2 viral infection on human oral health has not been systematically examined. COVID-19 can cause severe oral mucosa lesions and is likely to be connected with poor periodontal conditions. Fibroblasts are the major cell type inside periodontal ligament (PDL) and express the SARS-CoV-2 receptor: Angiotensin-converting enzyme 2 (ACE2), whose expression level can increase upon bacterial infection hence potentially provide a direct route of SARS-CoV-2 infection to PDL fibroblasts. In this research, we aimed to study the pathogenicity of SARS-CoV-2 viral components on human fibroblasts. We found that by exposing to SARS-CoV-2, especially to the viral envelope and membrane proteins, the human periodontal fibroblasts could develop fibrotic pathogenic phenotypes, including hyperproliferation that was simultaneously induced with increased apoptosis and senescence. The fibrotic degeneration was mediated by a down-regulation of mitochondrial β-oxidation in the fibroblasts. Fatty acid β-oxidation inhibitor, etomoxir treatment could mirror the same pathological consequence on the cells, similar to SARS-CoV-2 infection. Our results therefore provide novel mechanistic insights into how SARS-CoV-2 infection can affect human periodontal health at the cell and molecular level with potential new therapeutic targets for COVID-19 induced fibrosis.

## Introduction

Since its outbreak, the coronavirus disease 2019 (COVID-19) pandemic has infected over 761 million people globally (https://covid19.who.int) and has been a major challenge to human health. The severe acute respiratory syndrome coronavirus 2 (SARS-CoV-2 virus) has multiple transmission routes such as through respiratory fluids, therefore is highly infectious [1]. It has been reported that there were ever increasing “long COVID” [2] and repeated infection cases [3], with it has been reported in the UK alone having more than 2 million long COVID cases (https://www.ons.gov.uk/peoplepopulationandcommunity/healthandsocialcare/conditionsanddiseases/bulletins/prevalenceofongoingsymptomsfollowingcoronaviruscovid19infectionintheuk/30march2023) [4]. COVID-19 has been initially connected with acute inflammatory disease, which causes progressive pulmonary fibrosis [5], such as epithelial cell death has been considered as the essential driver of pulmonary fibrosis [6]. Increasing evidence have suggested that COVID-19 does affect other organs such as heart, skin, kidneys, and brain [7]. The human oral cavity and saliva have also been demonstrated to be an important reservoir of the SARS-CoV-2 virus [8], and saliva have been used for effective diagnosis of COVID-19 [9]. Periodontal tissues are highly vulnerable to different infectious diseases and COVID-19 has been reported to be potentially connected with poor periodontal health [10]. Furthermore, severe oral mucosa lesions including detachment of oral epithelium with inflammation and fibrosis have been reported in the COVID-19 patients [11, 12]. In particular, PDL is one of the most vulnerable human tissues that extrinsic virus and bacteria can often enter PDL directly in pathological conditions [13]. Consistent with this concept, previous reports have shown that ACE2 expression were elevated in the periodontal fibroblasts under gingivitis and periodontitis conditions [14], and could be induced by lipopolysaccharide, inflammatory cytokines etc. [15]. However, the molecular mechanisms under the potential pathological consequence of SARS-CoV-2 infection on human oral health have not been systematically investigated.

**Fig. 1:**
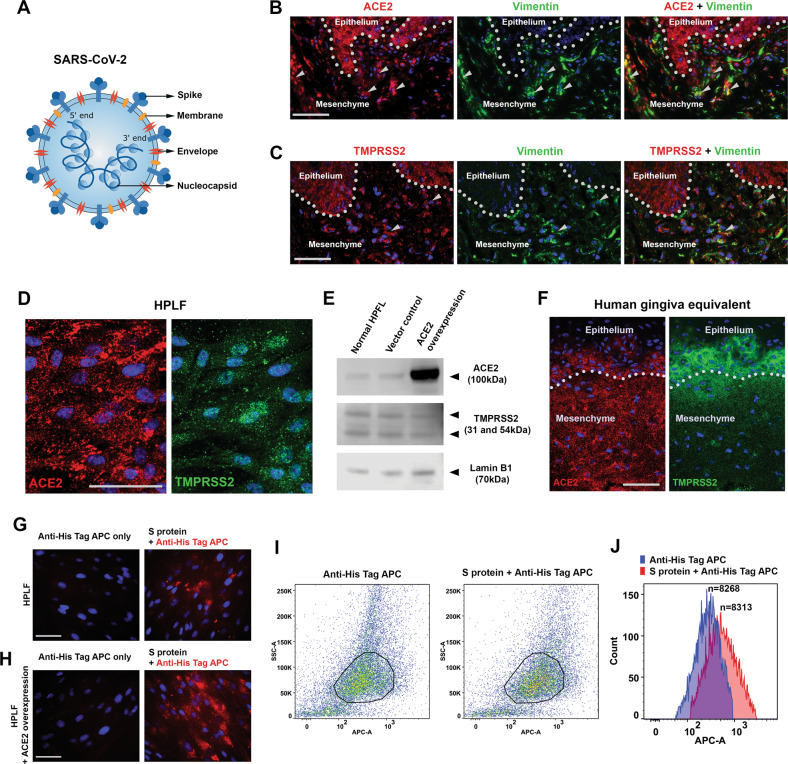
Human periodontal cells express ACE2 and TMPRSS2, and prone to SARS-CoV-2 infection. **A** Illustration of SARS-CoV-2’s key structure proteins; **B**, **C** Immunofluorescence analysis of ACE2 or TMPRSS2 co-expression with Vimentin in human periodontal tissues. using specific antibodies and Alexa 568 (red) or Alexa 488 (green) conjugated secondary antibodies. Dotted lines mark epithelia-mesenchymal junctions. Arrows indicate typical clusters of fibroblasts with co-expression of the indicated markers. **D** Immunofluorescence analysis of ACE2 or TMPRSS2 in human periodontal ligament fibroblasts (HPLF). **E** Western blotting analysis of ACE2 and TMPRSS2 expression in human periodontal ligament fibroblasts (HPLF) under normal growing condition, and lentiviral mediated ACE2 overexpression. Lamin B1 was used as loading control. All the blots were performed sequentially on the same membrane. For full blots please see Appendix Fig. 7A. **F** Immunofluorescence analysis of ACE2 or TMPRSS2 in human gingiva equivalent (also see Appendix Fig. 1A). **G**, **H** Normal HPLF or the cells with ACE2 overexpression was treated with His tagged spike (S) protein first then traced using Anti-His Tag APC conjugated antibodies. For controls, the spike protein was omitted (also see Appendix Fig. 1B). **I**, **J** FACS analysis of the samples showed in **E** and **F**. Note the anti-His Tag APC antibody did show background but the shifting of the florescence peak could still be visualized (**H**). n: particle (cell) count. Bars: 100 μm.

The evolution of SARS-CoV-2 has generated different variants that are responsible for infection speeds and symptoms (https://www.who.int/activities/tracking-SARS-CoV-2-variants). SARS-CoV-2 has four different structural protein components: envelope, membrane, nucleocapsid and spike (Fig. 1A) [16]. The mutations of SARS-CoV-2 predominantly occur in the spike proteins, particularly the receptor-binding domain (RBD), whilst the other key viral components remain stable [17, 18]. A key step of SARS-CoV-2 infection is the binding of spike RBD to angiotensin-converting enzyme 2 (ACE2) receptor on the target cells [19] and co-receptor transmembrane serine protease 2 (TMPRSS2) to trigger viral internalization together with the molecular downstream cascades [20]. Currently, most of the COVID-19 related research and vaccine development have focused on the spike protein, leaving the other structural proteins’ pathological functions remain to be elucidated.

As part of a series of research on the biological connection of COVID-19 with oral health, this study intended to use human periodontal tissue and fibroblasts as examples to dissect the direct pathological effects of SARS-CoV-2 viral structural components.

## Results

### Human periodontal tissues and fibroblasts express SARS-CoV-2 receptors

To achieve robust evidence of the presence of SARS-CoV-2 receptors, ACE2 and TMPRSS2 in human periodontal tissue and cells, we used immunofluorescence analysis and confirmed that both ACE2 and TMPRSS2 were notably expressed in the gingival epithelium, as well as in the periodontal ligament (PDL) cells (Fig. 1B, C). By analyzing the cultured human periodontal ligament fibroblast (HPLF), we could also confirm the presence of the two receptors by immunofluorescence (Fig. 1D) and western blotting analysis in those cells (Fig. 1E). With an established collagen gel-based 3D human gingiva tissue equivalent using gingival epithelial cells and HPLF (Appendix Fig. 1A), we also observed consistent clear expression of ACE2 and TMPRSS2 both in the epithelial cells and PDL fibroblasts (Fig. 1F).

### SARS-CoV-2 has high affinity to PDL fibroblasts

We next explored the pathogenic potential of SARS-CoV-2 in infecting PDL fibroblasts. By treating the cells with His Tag conjugated spike protein, followed by immunofluorescent analysis of the spike protein location using an anti-His Tag APC conjugated antibody (Appendix Fig. 1B), we observed a clear association of the spike protein with the cells both under natural growing condition (Fig. 1G), and following a lentiviral mediated ACE2 overexpression in PDL cells (Fig. 1H). The affinity of spike protein to the cells could be further validated by flow cytometry analysis (Fig. 1I, J). Therefore, we confirmed that SARS-CoV-2 indeed could infect PDL fibroblasts directly.

### SARS-CoV-2 envelope and membrane proteins induce fibrotic pathogenic phenotypes in PDL fibroblasts

**Fig. 2:**
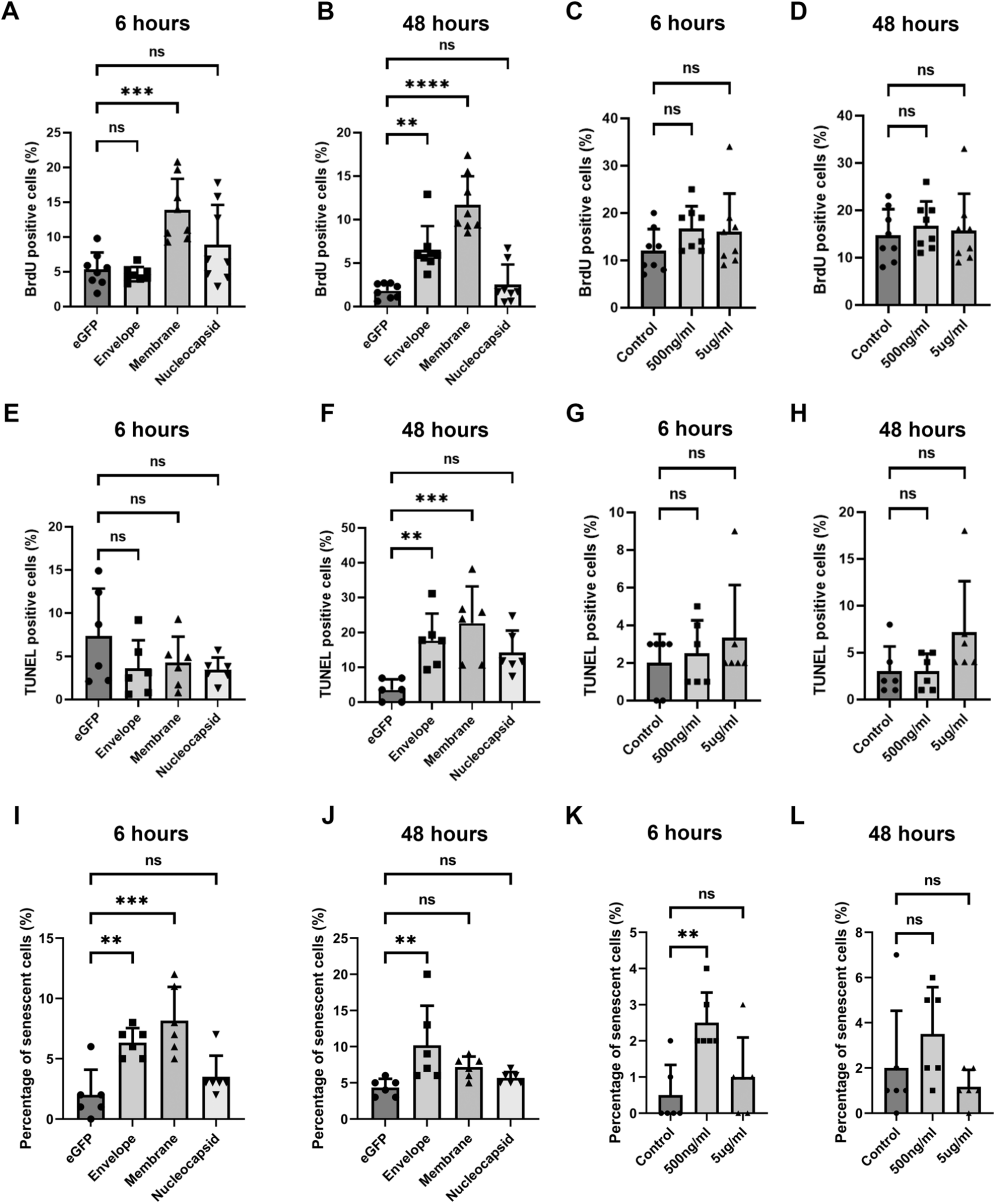
SARS-CoV-2 envelope and membrane protein could induce PDL fibroblast proliferation, apoptosis, and senescence. **A**–**D** BrdU incorporation analysis on the PDL fibroblasts treated with indicated conditions and time. Each data dot represent one random field in triplicated samples. Dunnett’s test was used for statistical analysis. Representative images can be found from Appendix Fig. 2. **E**–**H** TUNEL analysis on the PDL fibroblasts treated with indicated conditions and time. Each data dot represent one random field in triplicated samples. Dunnett’s correction was used for statistical analysis. Representative images can be found from Appendix Fig. 3. **I**–**L** Quantification of senescence-associated β-galactosidase positive cells (for identifying senescence) images on the indicated conditions. Each data dot represent one random field in triplicated samples. Representative images can be found from Appendix Fig. 4. ns no significance; **p < 0.01; ***p < 0.001.

A key pathological hallmark of COVID-19 infection is fibrosis in the lung and potentially also in the other tissues [5]. To evaluate the consequence of COVID-19 infection on the PDL, we either adopted lentiviral mediated SARS-CoV-2 infection for envelope, membrane or nucleocapsid proteins [21], or applied recombinant spike proteins at 500 ng/ml or 5 μg/ml, to cultured human PDL fibroblasts. We simulated acute and long term infection by infecting or treating PDLs for 6 h or 48 h. Cell proliferation was evaluated using Bromodeoxyuridine (BrdU) incorporation followed by immunostaining for anti-BrdU antibodies. The results indicated that under our tested conditions, only the membrane protein significantly increased cell proliferation at 6 h, while at 48 h, the envelope and membrane proteins both elevated the BrdU incorporation index (Fig. 2A, B and Appendix Fig. 2A). The spike protein, on the contrary, could not modulate cell proliferation (Fig. 2C, D and Appendix Fig. 2B).

We next evaluated apoptosis status in the cells with Terminal deoxynucleotidyl transferase dUTP nick end labeling (TUNEL) assay. The results indicated that at 48 h, again only the envelope and membrane proteins could increase apoptosis (Fig. 2E–H and Appendix 3A, B). In the meanwhile, for cellular senescence status in the tested conditions, the results showed that at 6 and 48 h, only the envelope and membrane protein groups resulted in a significant increase in senescent cells (Fig. 2I–L and Appendix 4A, B).

**Fig. 3:**
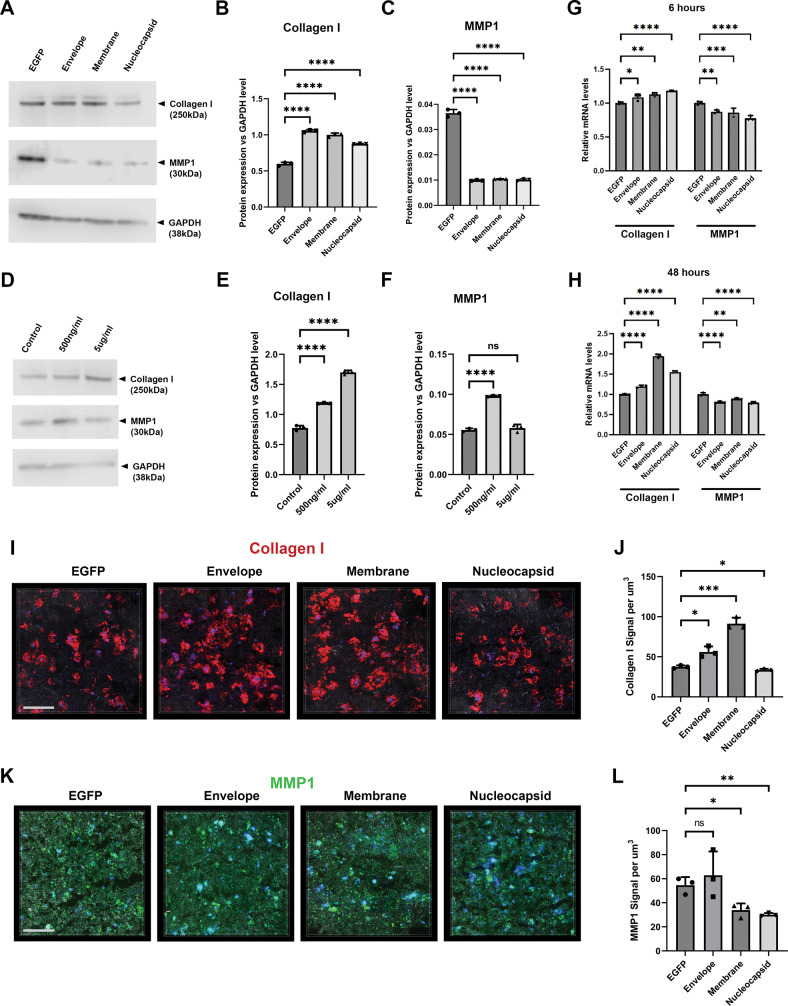
SARS-CoV-2 infection could induce collagen matrix deposition and MMP1 reduction. **A**–**F** Western blotting analysis of Collagen I and MMP1 production in the cells under indicated conditions. A shows blotting results. **B**, **C** represents statistical analysis (each dot represents a single individual measurement). Dunnett’s test was used for statistical analysis. For full blots for **A** and **D** please see Appendix Fig. 7B. ns no significance; **G**, **H** Real-time RT-PCR analysis of Collagen 1 and MMP1 transcription in the indicated conditions. **I**–**L** Representative three dimensional analysis of Collagen I and MMP1 expression in the indicated conditions at 7 days after seeding the cells and infection in the hydrogel. The gel were stained using anti-Collagen I and MMP1 specific antibodies and developed with Alexa 568 (red) or 488 (Green) conjugated secondary antibodies. Each data dot represent one random field in triplicated samples. ns no significance; *p < 0.05; **p < 0.01; ***p < 0.001; ****p < 0.0001. Bars: 100 μm.

We then investigated extracellular matrix production by focusing on Collagen I and Matrix Metallopeptidase 1 (MMP1), the two key components and enzymes responsible for PDL tissue integrity. Interestingly, western blotting analysis revealed that all of the SARS-CoV-2 structural proteins could induce Collagen I production (Fig. 3A–F), and reduce MMP1 production, and the spike protein elevated MMP1 expression under one of the two tested doses (Fig. 3A–F). Real-time RT-PCR analysis suggested the induction of Collagen I expression and MMP1 downregulation were modulated at transcription levels (Fig. 3G, H). By applying a hydrogel-based 3D culture system, we evaluated Collagen I and MMP1 production, and deposition in three-dimensions. The results showed that Collagen I deposition were again highly elevated, especially in the envelope and membrane protein groups (Fig. 3I, J). And membrane and nucleocapsid proteins could reduce the MMP1 expression (Fig. 3K, L).

Together, with the evidence of elevated fibroblast proliferation, apoptosis, and senescence concomitantly, alongside the increased matrix deposition and reduced MMP1 production, our results indicated that the SARS-CoV-2’s envelope and membrane were the most potent components to induce a fibrotic degeneration phenotype in PDL cells.

### SARS-CoV-2 envelope and membrane proteins down-regulate mitochondrial β-oxidation

**Fig. 4:**
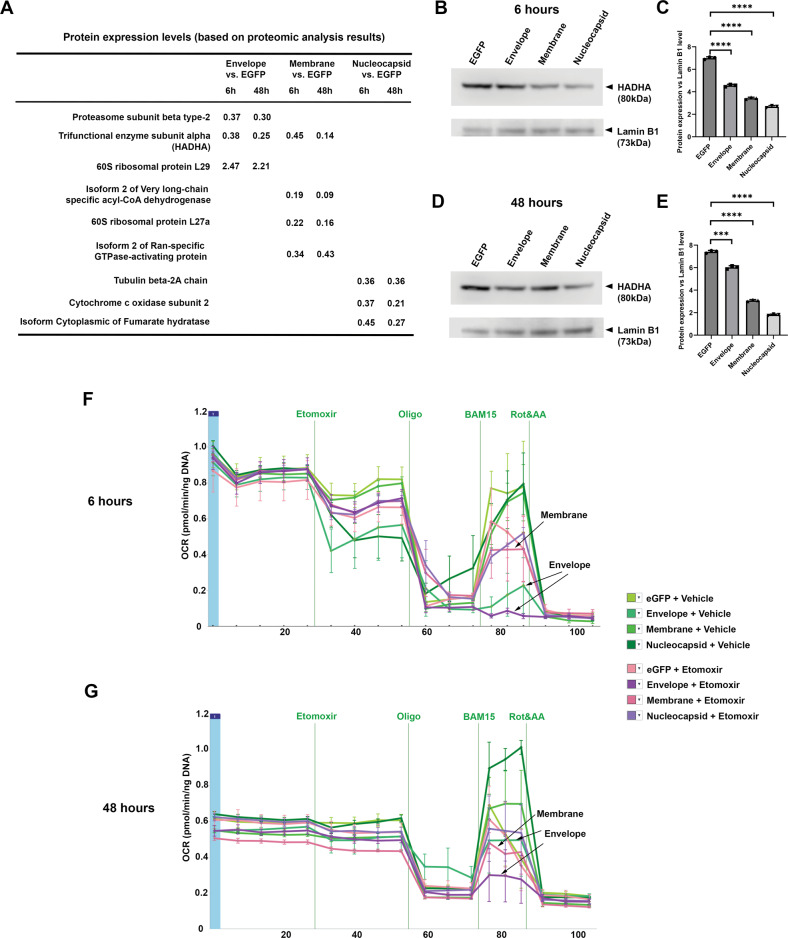
Envelope and membrane proteins are responsible to SARS-CoV-2 infection to PDL fibroblasts caused deregulate of mitochondria fatty acid β-oxidation pathway. **A** Summary of proteomic analysis results of PDL fibroblasts treated with indicated conditions and time points. Only proteins showed <0.50 or >1.5 fold changes were included. For full data analysis please see Appendix Table 1. **B**–**E** Western blotting analysis of HADHA expression in HPLF treated under indicated viral infection conditions either for 6 h (**B**, **C**), or 48 h (**D**, **E**). **C**, **E** represent statistical analysis (each dot represents a single individual measurement). ***p < 0.001; ****p < 0.0001. Lamin B1 was used as loading control. All the blots were performed sequentially on the same membrane. For full blots of **B** and **D** please see Appendix Fig. 7C, D. **F**, **G** 6 and 48 h Seahorse palmitate oxidation stress tests under indicated conditions either with vehicle alone or Etomoxir. Note for both time points, envelope group were down-regulated with/without etomoxir treatments, while membrane group also showed down regulation but only in the etomoxir treated samples.

To understand the molecular regulation mechanisms behind the observed pathological consequence of SARS-CoV-2 infection, we performed proteomic analysis on the treated PDL fibroblasts, by focusing on the effects of the envelope, membrane and nucleocapsid proteins. We then identified a group of proteins that were both suppressed at 6 and 48 h post infection (Fig. 4A and Appendix Table 1). Among the significantly down-regulated proteins, the trifunctional enzyme subunit alpha, isoform 2 of very long-chain specific acyl-CoA dehydrogenase, cytochrome c oxidase subunit 2 and isoform cytoplasmic of fumarate hydratase are all essential enzymes in mitochondria functions and mitochondrial β-oxidation (Fig. 4A). Particularly the trifunctional enzyme subunit alpha (HADHA), isoform 2 of very long-chain specific acyl-CoA dehydrogenase were connected with mitochondrial fatty acid β-oxidation. In addition, western blotting analysis could further confirm that HADHA was indeed down-regulated by SARS-CoV-2 structural protein infection (Fig. 4B–E).

We therefore conducted Seahorse Mito stress tests (Appendix Fig. 5) to evaluate if and how the mitochondria fatty acid pathway’s function could be affected by SARS-CoV-2 infection. The results showed that indeed, both SARS-CoV-2 envelope and membrane protein, could inhibit fatty acid β-oxidation at both 6 h and 48 h time points (Fig. 4F, G).

### Chemical inhibition of mitochondrial β-oxidation can mirror the fibrotic pathological consequence of SARS-CoV-2 infection

**Fig. 5:**
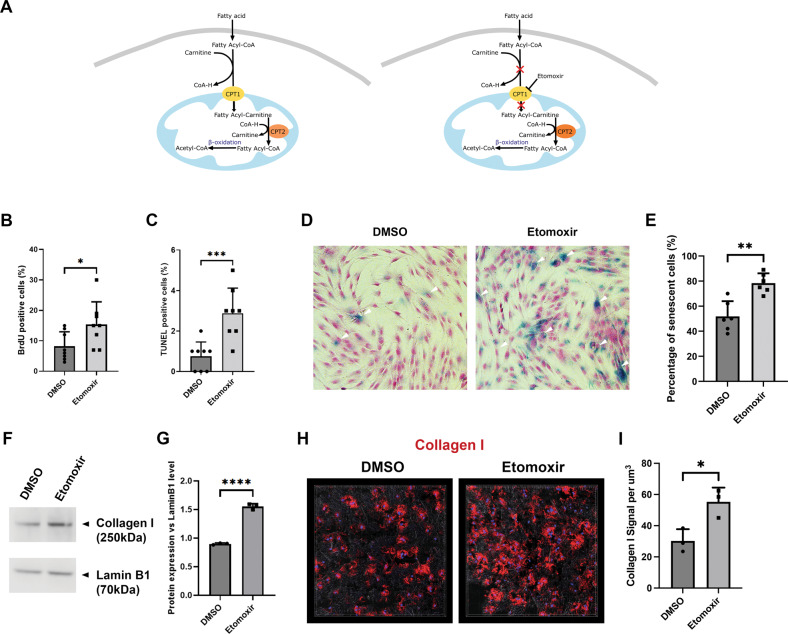
Mitochondrial fatty acid β-oxidation inhibition mirrored fibrotic degeneration phenotypes in PDL fibroblasts. **A** Schematic drawing of fatty acid β-oxidation pathway (left) and the Etomoxir functioning mechanism (right). **B**, **C** BrdU and TUNEL analysis of etomoxir treated cells. For representative images please see Appendix Fig. 6. Each data dot represent one random field in triplicated samples. **D**, **E** Representative senescence-associated β-galactosidase assay on indicated conditions. Note increased senescence in the etomoxir treated samples. Each data dot represent one random field in triplicated samples. **F**, **G** Western blotting analysis of Collagen I expression in HPLF under etomoxir treatment or vehicle (DMSO) alone. Lamin B1 was used as loading control. All the blots were performed sequentially on the same membrane. **G** represents statistical analysis (each dot represents a single individual measurement). For full blots please see Appendix Fig. 7E. **H**, **I** Representative 3D analysis of Collagen I expression in the indicated conditions at 48 hours after seeding the cells and etomoxir treatment in the hydrogel. The gel was stained using anti-Collagen I specific antibodies and developed with Alexa 568 (red) conjugated secondary antibodies. Each data dot represent one random field in triplicated samples. *p < 0.05; **p < 0.01; ***p < 0.001; ****p < 0.0001. Bars: 100 μm.

To validate the effect of fatty acid β-oxidation pathway inhibition, we next treated the cells with etomoxir, a specific inhibitor of the pathway through inhibiting carnitine palmitoyltransferase I (CPT1), a key regulatory enzyme for fatty acid to be imported into mitochondria (Fig. 5A). The results indicated that etomoxir treatment could indeed mirror the same pathological consequence on the fibroblasts of the SARS-CoV-2 infection. We again observed significantly increased cell proliferation (Fig. 5B and Appendix Fig. 6), apoptosis (Fig. 5C and Appendix Fig. 6), and senescence (Fig. 5D, E), together with elevated Collagen I production (Fig. 5F, G). Similarly, in the hydrogel-based three-dimension PDL fibroblasts culture, Collagen I deposition was highly increased in the etomoxir treated samples (Fig. 5H, I).

## Discussion

COVID-19 infection can cause a series of symptoms. Among them, fibrosis particularly in lung tissues has evoked significant attention due to the severe consequence on patient quality of life. Although the current dominant SARS-CoV-2 variants (such as the Omicron) induce milder symptoms in human bodies, the exact pathological consequence of COVID-19 to different human tissues and organs, particularly for oral cavity tissues are still missing, possibly due to the difficulty of distinguishing conventional periodontal diseases from COVID-19 caused pathological changes. Current concepts of COVID-19 etiology suggest lung fibrosis caused by SARS-CoV-2 infection can be mainly due to damages in lung epithelial cells that trigger acute inflammation followed by fibroblast hyperproliferation [22]. However, since SARS-CoV-2 infection is rapid, and in oral cavity the epithelial barrier could be rapidly disrupted upon infection [11], it is an intriguing question if the deeper cells such as fibroblasts can also be infected directly by the virus and trigger pathological changes. As such, it is reasonable to postulate that SARS-CoV-2 can also directly infect PDL fibroblasts in the already damaged PDL, and can induce further pathological changes or enhance existing periodontal lesions. Attentions therefore should be made by dental clinicians to the patients who had COVID-19 and appeared to be diagnosed with periodontal disease at the same time, particularly for those long COVID and repeated infected cases.

Mitochondrial fatty acid β-oxidation is the major pathway responsible for fatty acids degradation, hence is essential for human body energy homeostasis, impeding the pathway can cause different disorders [23]. Very recent studies have observed mitochondrial dysregulation in COVID-19 patient blood cells [24, 25]. Interestingly, the dysfunction of the fatty acid oxidation has been previously connected with fibrosis particularly in the lung and kidney [26, 27]. Our findings further confirmed that in the fibroblasts, SARS-CoV-2 could possibly induce fibrotic degeneration directly, through downregulating fatty acid β-oxidation, particularly through the virus’ envelope and membrane proteins.

Among the SARS-CoV-2’s structural proteins, envelope, membrane, nucleocapsid, and spike, the first three proteins are stable structure proteins for all the reported variants, while so far all of the identified mutations happen inside the spike proteins [18]. Although spike protein is still the target especially for COVID-19 vaccine development, increasing evidence suggest the other SARS-CoV-2 structural proteins might have unexpected yet important roles in inducing COVID-19 symptoms. Previous structural analysis of the envelope protein suggested it might be important for virus pathogenicity [28]. The envelope protein can also physically increase intra-Golgi pH and forms cation channel [29], and biochemically modulate spike protein in the meantime [30]. The most abundant protein, the membrane protein in the SARS-CoV-2 virus, is rationally important for virus assembly [31]. Our results further confirmed that the envelope and membrane proteins are actually responsible for the fibrosis phenotypes in the cells by down regulating the key fatty acid β-oxidation regulator, such as the trifunctional enzyme subunit alpha. The molecular mechanism behind this regulation axis would require further biochemical analysis.

In this study, our results provide a novel insight into the mechanisms of SARS-CoV-2 infection in PDL fibroblasts and its dysregulation of intracellular events leading to the pathologic phenotype of fibrosis. The findings could be possibly extended to the other body systems to explain and explore the COVID-19 caused fibrosis pathology and treatment.

## Materials and methods

### Cell culture

Human periodontal ligament fibroblasts (HPLF) were purchased from ScienCell (Cat. 2630) and cultured in DMEM/F12 ((Gibco, 31331-028) containing 20% Fetal bovine serum (FBS) (Sigma, F7524), 1% penicillin-streptomycin (Hyclone, SV30079.01). Human gingival epithelial cells (HGEPp, CELLnTEC, Cat. HGEPp) were cultured in CnT-57 (CELLnTEC, CnT-57). For both cell lines, passage 3–6 cells were used.

### Lentiviral infection

Plasmids for SARS-CoV-2 structural proteins were purchased from Addgene (Appendix Table 2). Lentiviruses were prepared using JetOPTIMUS (Polyplus) mediated transfection of HEK293T cells. Lentiviral supernatant was collected according to the manual using 293FT cells. HPLFs were infected with lentiviruses carrying the target sequences above with 10 μg/ml polybrene (Merck, TR-1003). After 2 h, dishes were topped up with fresh culture medium. Samples were collected at 6 h or 48 h. For overexpressing ACE2 (Addgene, Appendix Table 2) in HPLFs, the cells were incubated with lentiviral particles carrying target sequences above with 10 μg/ml polybrene (Merck). After 2 h, dishes were topped up with fresh normal culture medium. Samples were collected according to different time points. Infected cells were selected with 1 μg/ml puromycin (Thermo Scientific, 10781691) for 7–10 days. Samples were collected either 6 hours or 48 h after infection.

### Recombinant SARS-CoV-2 spike protein treatment

Recombinant SARS-CoV-2 spike protein with His-tag (Biotechne, 10549-CV) was diluted with culture medium to 500 ng/ml or 5 μg/ml and added into the cell culture medium. Samples were collected at 6 h or 48 h.

### Mitochondria β-oxidation inhibition assay

Etomoxir (Sigma, E1905) was diluted with culture medium to the defined concentration then added into cell culture medium. Samples were collected at 6 h or 48 h.

### Human periodontal tissue 3D equivalents

In all, 5 × 10^4^ of HPLFs were mixed with 150 μl gel mixture of rat tail collagen (Fisher, 11519816), DMEM (Fisher, 21969-035) and FBS (Ratio of volume 9:1:1) homogenously on ice. 2.3 μl 1 M Sodium hydroxide solution (NaOH, Sigma 71687) was added into the mixture for neutralization, 150 μl gel was pipetted into a 0.4 μm culture insert (Greiner, 662641) incubated at 37 °C for 1 h then fresh culture medium was added into the insert. Culture medium was replaced by CnT-57 before seeding 5 × 10^5^ of HGEPp on top of the gel. HGEPp was cultured for 72 h before airlifting. Culture medium inside the insert was removed every day and the medium outside was changed every 2 days. Samples were frozen directly in OCT (Agar Scientific, AGR1180) at day 14 and sectioned at 20 μm for further analysis.

### Hydrogel-based 3D matrix production assay

In all, 1 × 10^6^ of HPLFs were mixed with 100 μl bioink (CELLINK, CELLINK SKIN + ) slowly and gently. Gels were pipetted into six-well plate then 1.5 ml crosslinking agent (CELLINK) was added to cover the gel at RT for 5 min. Crosslinking agent was removed and 1.5 ml fresh culture media was added which was replaced every 2 days. Samples were frozen directly in OCT at day 7 and sectioned at 30 μm for further analysis.

### Immunohistochemistry

For the details of immunostaining, BrdU incorporation assay, Terminal deoxynucleotidyl transferase dUTP nick end labeling (TUNEL) assay and Senescence assay details please see Appendix materials and methods.

### Flow cytometry analysis

HPLFs and ACE2 overexpressed HPLFs were harvested and fixed in 2% PFA solution in 10 mM PBS for 10 min then washed with FACS buffer (1% BSA in PBS). In all, 1 × 10^6^ cells were resuspended in 500 μl flow cytometry permeabilization buffer (0.1% Tween-20 in PBS) for 15 min then washed with FACS buffer again. Cells were resuspended in 100 μl FACS buffer and APC His-Tag conjugated antibody was added, incubated for 2 h at room temperature then kept in dark at 4 °C degree overnight. Samples were analyzed using the BD FACSAria™ II (BD Biosciences). Data was acquired using red laser (633–640 nm) for APC signal. Results were analyzed using the FlowJo software (Tree Star Inc., Version 10.8.1). Gates and regions were placed around populations of cells with common characteristics based on SSC and APC.

### Western blotting

A NuPage® Electrophoresis System (Thermo Fisher Scientific), 4-12% Bis-Tris gradient gel (Thermo Fisher Scientific, NP0335BOX), and MOPS buffer (Thermo Fisher Scientific, NP0001). In all, 25–40 µg protein were used for protein separation on a 4–12% Bis-Tris gradient gel (Thermo Fisher Scientific, NP0335BOX) with MOPS buffer (Thermo Fisher Scientific, NP0001) of NuPage® Electrophoresis System (Thermo Fisher Scientific). Transfer of protein samples onto a 0.45 µm PVDF membrane (Thermo Fisher Scientific, LC2005) was carried out using a NuPage® XCell II Blot Module, and transfer buffer (Thermo Fisher Scientific, NP0006) with 10% methanol (Sigma, 322415). The iBind™ Western System (Thermo Fisher Scientific) was used for blocking, primary and secondary antibody incubations which details could be found above. A C-Digit scanner (LI-COR) was used for band detection with Image studio software (LI-COR, Version 3.1).

### Proteomic analysis

Sample preparation, in-gel digestion, sample cleanup and mass spectrometric analysis was carried out as described in the Appendix material and methods. The mass spectrometry proteomics data have been deposited to the ProteomeXchange Consortium via the PRIDE [32] partner repository with the dataset identifier PXD041281.

### Real-time PCR and data analysis

Real-time RT-PCR analysis was performed on a LightCycler 480 Real-Time system (Roche) for 45 cycles, using a SYBR Green I MasterMix (Roche, 04887352001) and primers. 36β4 gene was used as housekeeping gene. Analyses were performed using three technical replicates using the 2^−ΔΔCt^ method.

### Seahorse mito stress test

In all, 3 × 10^3^ of HPLFs per well were seeded into Seahorse XFe96 well plate (Agilent Technologies, 200941) for overnight. After 6 h and 48 h of viral infection, the medium was removed but a nominal 20 μl per well was left. Each well was washed twice with pre-warmed assay medium (Agilent Technologies, 103680). In all, 80 μl assay medium were added into each well, and cells were then incubated for 1 h at 37 °C. Meanwhile, effector working solutions were prepared and loaded into ports of the XFe96 cartridge which was already rehydrated in XF buffer at 37 °C overnight. The cartridge and the utility plate were inserted into XFe96 instrument to calibrate probes. Once the calibration was finished, the utility plate was replaced by cell plate and continued with the assay as indicated. When the progress was completed, cell plate was removed from the instrument. Medium was aspirated slowly from wells and all wells were gently washed with warmed assay buffer, Plates were stored at −20 °C or continued with DNA assay for further normalization. Data were analyzed by Seahorse Analytics website and Wave software (Agilent Technologies, Version 2.6.3).

### Statistics

Statistical analyses were performed using Prism software (GraphPad software, Version 9.4.1). Unpaired *t*-test was applied to all measurements. Data from Seahorse mito stress assay was analyzed by Seahorse Analytics website and Wave software (Agilent Technologies, Version 2.6.3). All quantification and real-time RT-PCR results were presented using style of mean and standard deviation (error bars). Observed differences were calculated for *p*-values: *p < 0.05; **p < 0.01; ***p < 0.001; ****p < 0.0001.

## Supplementary information

Appendix

Appendix Figure 1

Appendix Figure 2

Appendix Figure 3

Appendix Figure 4

Appendix Figure 5

Appendix Figure 6

Appendix Figure 7

Appendix Table 1

Appendix Table 2

## Data Availability

The materials used and datasets generated during and/or analyzed during the current study are available from Professor Bing Hu on reasonable request.
