# Supplementary material for: SARS-CoV-2 infection causes periodontal fibrotic pathogenesis through deregulating mitochondrial beta-oxidation
Source: Cell Death Discov. 2023 May 26;9:175. doi: 10.1038/s41420-023-01474-2 (PMC10214333; doi:10.1038/s41420-023-01474-2)
Supplement: Supplementary file 1 — Appendix [file 41420_2023_1474_MOESM1_ESM.docx]

***SARS-CoV-2* infection causes periodontal fibrotic pathogenesis through deregulating mitochondrial beta-oxidation**

Yan Gao^1^, Wai Ling Kok^1^, Vikram Sharma^2^, Charlotte Sara Illsley^1^, Sally Hanks^1^, Christopher Tredwin^1^, Bing Hu^1*^

1. Stem Cells & Regenerative Medicine Laboratory, Peninsula Dental School, Faculty of Health, University of Plymouth, 16 Research Way, Plymouth, PL6 8BU, UK

2. School of Biomedical Sciences, Faculty of Health, University of Plymouth, 16 Research Way, Plymouth, PL6 8BU, UK

* Corresponding author

Professor Bing Hu

Email: bing.hu@plymouth.ac.uk

**Appendix Materials & Methods**

**Appendix Figure 1 to 7**

**Appendix Table 1 and 2**

**Appendix Materials & Methods**

**Immunostaining**

The human oral cavity cancer tissue array containing normal human gingiva tissues, HnTMA108 was purchased from Creative Bioarray. Slides were heated to 60°C for 20 min before being twice washed in xylenes (Sigma Aldrich 534056) for 10 mins. rehydrated with 100% industrial methylated spirit (IMS) (VWR, 23684.360) for 5 min, before being washed for 2 min in 95% IMS and then 70% IMS. Antigen retrieval was performed in a 95°C water bath, using pre-warmed 0.01 M citrate buffer solution (citric acid (Sigma Aldrich, C2404) & 0.05% Tween-20 (Sigma Aldrich, P9416)), pH 8.0 for 20 min. Slides were washed briefly in tap water before washed 3 times in phosphate buffered saline (PBS, Sigma, P4417) containing 0.1% Triton-X100 (Sigma, X100) (PBST) for 5 min per wash. Non-Specific binding was blocked by incubation for 60 min with PBST containing 5% Donkey Serum (Sigma, D9663). Primary antibodies were incubated overnight at 4°C. Slides were washed 3 times in PBST before incubation with secondary antibodies for 2 h at room temperature. Nuclei were counterstained with 2 μg/ml DAPI (Sigma Aldrich, D9542) for 10 min, after which slides were mounted with Dako fluorescent mounting medium (Dako North America Inc., S3023).

For frozen section, the slides were air-dried for 1 h at RT then fixed with 4% paraformaldehyde (PFA; Sigma in PBS) for 30 min, then washed twice with PBST 5 min each. Primary antibodies were incubated overnight at 4°C. Slides were washed three times in PBST before incubation with secondary antibodies for 2 h at RT. Nuclei were counterstained with 2 μg/ml DAPI for 10 min. Slides were mounted with Dako fluorescent mounting medium.

Immunofluoresence images were captured using a Leica DMI6000 confocal microscope with a Leica TCS SP8 attachment at a scanning thickness of 1 μm per section. The microscope is equipped with LAS X software (Leica, Version 3.7.3.23245). Images for comparison were taken using the same settings and post imaging processing was conducted using Adobe Photoshop (Adobe, Version 24.0.1). Images of 3D equivalents were visualized by Imaris software (Bitplane, Version 9.0.2).

**BrdU incorporation assay**

HPLFs were seeded in a black 96 well plate (GBO, 655090) at a confluency of 5x10^3^ cells per well. Following HPLF treatment with the spike protein or lentiviral infection, BrdU cell prolife labelling reagent (Amersham-GE, RPN201, 1:1000) was diluted in complete cell culture medium then added into dishes for 2-3 h at 37°C in a humidified incubator. Following incubation with BrdU, HPLF cells were washed 3 times with PBS for 2 min each, then fixed in 4% PFA for 30 min, followed by washing twice in PBS. Cells were treated with 100 μl 2N hydrochloric acid (HCl) (Sigma, H1758-100ml) for 30 min before they were stained with anti-BrdU antibody (Abcam, ab6326, 1:500). The antibody staining procedure followed our immunofluorescence staining protocol, with the exception that the blocking buffer was prepared with 2.5% bovine serum albumin (BSA) (Sigma. A2153) in PBST. Images were processed in Image J software (National Institutes of Health, Bethesda, MD, USA, Version 1.53k). BrdU-positive cells and total cell number were quantified using ‘Analyze Particles’ function.

**Terminal deoxynucleotidyl transferase dUTP nick end labelling (TUNEL) assay**

HPLFs were seeded in a black 96 well plate at a confluency of 5x10^3^ cells per well. Following HPLF treatment with the spike protein or lentiviral infection, cells were washed twice in PBS for 5 sec, fixed using 4% PFA for 30 min and then washed twice in PBS for 5 min. Cells were permeabilized on ice using PBST for 2 min, then incubated in 50 μl TUNEL mixture (In Situ Cell Death Detection Kit, Fluorescein, version 17, Roche) for 2 h at 37°C in a humidified incubator.

**Senescence assay**

HPLFs were seeded in a 24 well plate at a confluency of 5x10^4^ cells per well. After the spike protein treatment or lentiviral infection, cells were washed briefly with PBS, cells were fixed for 15 min at RT using the senescence kit fixative solution (Senescence b-galactosidase kit, Cell Signaling, 9860S). Cells were washed twice using PBS, then the β-Galactosidase staining solution, pH 6.0, was added into each well. β-Galactosidase staining solution at pH 4.0 was used as a positive control. The plate was incubated at 37°C overnight in a dry incubator without CO_2_. After incubation, cells were washed twice with PBS, then stained with nuclear fast red solution (Sigma, N8002) for 20 min, then washed with distilled H_2_O. All wells were mounted with 70% glycerol (Sigma, G2025) and images were taken with Leica IM8, image processing and quantification was conducted using Adobe Photoshop.

**Proteomic analysis**

HPLFs were seeded in 6 cm dishes for viral infection and protein treatment. Total protein was collected from cells 6 h and 48 h after treatment. Total protein was collected from cells by removing cell culture medium, washed twice in ice-cold HBSS. HBSS was removed and replaced with ice-cold RIPA buffer (ThermoFisher, 89901) supplemented with Halt™ Protease and Phosphatase Inhibitor Cocktail (ThermoFisher, 78440) at 1:100. The cells were detached from the dish using a cell scraper and then collected into an Eppendorf tube on ice, then incubated for 30 min with frequent agitation for efficient cell lysis and solubilisation of proteins. Tubes were spun down at 15,000 rpm for 15 min at 4°C so the supernatant containing the protein could be collected and stored at -80°C until ready to load onto a gel. 15 µg protein samples were run on a NuPAGE 4-12% Bis-Tris protein gel at 200V for 45 min. Gel was rinsed with water before fixing in 40% ethanol and 10% acetic acid for 15 min with gentle agitation. After washing the gel twice in water, the gel was stained overnight at RT in QC colloidal Coomassie Blue G-250 (Biorad, 161-0803). The gel was destained for 1 h with changes of water every 15 min until protein bands were visible. Every sample lane was cut into 4 fractions and each fraction further cut into 1-2 mm cubes for equilibration. In-gel digestion, sample cleanup and mass spectrometric analysis was carried out as described previously (1). All samples were stored at -20°C or analysed directly using mass spectrometry.

**Reference**

1. Dunn J, Ferluga S, Sharma V, Futschik M, Hilton DA, Adams CL, et al. Proteomic analysis discovers the differential expression of novel proteins and phosphoproteins in meningioma including NEK9, HK2 and SET and deregulation of RNA metabolism. EBioMedicine. 2019;40:77-91.

**Appendix Figure legend**

**Appendix Figure 1. Illustration of human gingiva equivalent culture and spike protein binding assay.**

A. For human gingiva equivalent culture, HPLF were seeded into collagen gel supported by a cell culture insert cylinder. 4 days later human gingival epithelial cells were seeded on top of the culture. After a further 4 days, the culture were lifted to air-liquid interface to allow stratification for 14 days before further analysis.

B. To test the binding of spike protein to HPLF cells, the cells were treated with His Tag conjugated spike protein first then traced using anti-His Tag APC conjugated antibodies. For control, the spike proteins were omitted.

**Appendix Figure 2.** **SARS-CoV-2 envelope and membrane protein could induce PDL fibroblast proliferation.**

Representative field images of BrdU incorporation analysis in the indicated conditions. Quantitative analysis can be found from Figure 2 A-D.

Bars: 100μm

**Appendix Figure 3.** **SARS-CoV-2 envelope and membrane protein could induce PDL fibroblast apoptosis.**

Representative field images of TUNEL analysis in the indicated conditions. Quantitative analysis can be found from Figure 2 E-H.

Bars: 100μm

**Appendix Figure 4.** **SARS-CoV-2 envelope and membrane protein could induce PDL fibroblast senescence.**

Representative field images of senescence analysis in the indicated conditions. The cells were countered stained with nuclear fast red. Note increased blue staining (marking senescent cells) in the envelope and membrane groups. Quantitative analysis can be found from Figure 2 I-L. White arrows indicate representative positive cells.

Bars: 100um

**Appendix Figure 5.** **Illustration of Seahorse mito stress test, for which drugs were added sequentially into the cell culture and what have been measured by the machine.**

**Appendix Figure 6. Mitochondrial fatty acid β-oxidation inhibition mirrored fibrotic degeneration phenotypes in PDL fibroblasts.**

Representative field images of BrdU incorporation and TUNEL analysis in the indicated conditions. Quantitative analysis can be found from Figure 5 B and C.

**Appendix Figure 7. Full blot images for the Western Blotting experiments used in this study.**

All the membranes were first stained with Ponceau S then blotted with the corresponding antibodies sequentially (from left to right)

**A.** Corresponding to Figure 1E;

**B.** Corresponding to Figure 3 A and D (which are on the same membrane);

**C.** Corresponding to Figure 4B;

**D.** Corresponding to Figure 4C;

**E.** Corresponding to Figure 5F.

**Appendix Table 1**

The original proteomic analysis data of the effects of SARS-CoV-2 structural proteins, as showed in Figure 4A.

**Appendix Table 2**

The key materials and reagents used in the current study.
