# Supplementary material for: SARS-CoV-2 infection causes periodontal fibrotic pathogenesis through deregulating mitochondrial beta-oxidation
Source: Cell Death Discov. 2023 May 26;9:175. doi: 10.1038/s41420-023-01474-2 (PMC10214333; doi:10.1038/s41420-023-01474-2)
Supplement: Supplementary file 9 — Appendix Table 1 [file 41420_2023_1474_MOESM9_ESM.docx]

**Appendix Table 1**

The original proteomic analysis data of viruses infected HPLFs showed in Figure 4A.

| **Accession** | **Protein** | **LFQ intensity eGFP 6hrs** | **LFQ intensity Envelope 6hrs** | **LFQ intensity Membrane 6hrs** | **LFQ intensity Nucleocapsid 6hrs** |
| --- | --- | --- | --- | --- | --- |
| H3BTL1 | H3BTL1_HUMAN Microtubule-associated protein 1 light chain 3 beta, isoform CRA_f OS | 20.10239029 | 19.10513687 | 20.0364418 | 20.03939056 |
| Q99613-2 | EIF3C_HUMAN Isoform 2 of Eukaryotic translation initiation factor 3 subunit C OS | 18.629179 | 19.96310425 | 20.06723404 | 20.2131176 |
| F8VZJ2 | F8VZJ2_HUMAN Nascent polypeptide-associated complex subunit alpha OS | 22.0500946 | 21.60272598 | 18.8843956 | 21.94813538 |
| O00148 | DX39A_HUMAN ATP-dependent RNA helicase DDX39A OS | 20.24852562 | 19.92243576 | 20.19075012 | 20.36442184 |
| O00159-2 | MYO1C_HUMAN Isoform 2 of Unconventional myosin-Ic OS | 20.42316437 | 20.65970612 | 21.64368057 | 18.09388161 |
| O00231 | PSD11_HUMAN 26S proteasome non-ATPase regulatory subunit 11 OS | 21.50232315 | 20.77643585 | 18.8217926 | 21.38958359 |
| O00232-2 | PSD12_HUMAN Isoform 2 of 26S proteasome non-ATPase regulatory subunit 12 OS | 19.38958931 | 18.97323799 | 18.15907097 | 19.32212639 |
| O00299 | CLIC1_HUMAN Chloride intracellular channel protein 1 OS | 23.82948685 | 23.17922211 | 23.2844677 | 23.52719116 |
| O00303 | EIF3F_HUMAN Eukaryotic translation initiation factor 3 subunit F OS | 20.16717339 | 19.47286606 | 18.07906342 | 19.43161583 |
| O00410 | IPO5_HUMAN Importin-5 OS | 21.63754654 | 21.14910889 | 21.34261513 | 21.50586319 |
| A0A2R8Y5G6 | A0A2R8Y5G6_HUMAN RNA helicase OS | 20.2237606 | 20.15783119 | 20.38268661 | 20.18555832 |
| O14818 | PSA7_HUMAN Proteasome subunit alpha type-7 OS | 21.17542076 | 21.4470787 | 21.25014496 | 22.0280323 |
| P19105 | ML12A_HUMAN Myosin regulatory light chain 12A OS | 22.9598732 | 22.81103897 | 22.7803669 | 23.33674431 |
| O15143 | ARC1B_HUMAN Actin-related protein 2/3 complex subunit 1B OS | 21.7553997 | 21.41776848 | 20.67959976 | 21.02337074 |
| O15144 | ARPC2_HUMAN Actin-related protein 2/3 complex subunit 2 OS | 21.11825943 | 20.74419022 | 20.81472015 | 20.75881767 |
| A0A286YF22 | A0A286YF22_HUMAN D-3-phosphoglycerate dehydrogenase OS | 22.21483803 | 20.47070313 | 20.90087509 | 21.23543739 |
| O43242 | PSMD3_HUMAN 26S proteasome non-ATPase regulatory subunit 3 OS | 18.42886925 | 19.4923172 | 18.51243401 | 20.35412407 |
| O43707 | ACTN4_HUMAN Alpha-actinin-4 OS | 24.45920181 | 24.34854317 | 24.44373131 | 24.58663368 |
| O43795-2 | MYO1B_HUMAN Isoform 2 of Unconventional myosin-Ib OS | 19.75842857 | 19.13761139 | 19.4098053 | 17.52830315 |
| O43852 | CALU_HUMAN Calumenin OS | 23.00316048 | 23.20448685 | 23.1497612 | 22.97472 |
| O60506-4 | HNRPQ_HUMAN Isoform 4 of Heterogeneous nuclear ribonucleoprotein Q OS | 18.74744225 | 22.43349266 | 21.80931282 | 22.75505257 |
| O60664-4 | PLIN3_HUMAN Isoform 4 of Perilipin-3 OS | 23.39806557 | 22.11977959 | 21.64412117 | 22.92772293 |
| O60701 | UGDH_HUMAN UDP-glucose 6-dehydrogenase OS | 23.65567398 | 23.56685829 | 23.33824158 | 23.71785164 |
| O60763 | USO1_HUMAN General vesicular transport factor p115 OS | 19.5602951 | 20.07678986 | 20.93315506 | 19.38621902 |
| O75083 | WDR1_HUMAN WD repeat-containing protein 1 OS | 22.13138199 | 21.2696228 | 23.37650108 | 23.52037239 |
| E5RIW3 | E5RIW3_HUMAN Tubulin-specific chaperone A OS | 20.52305603 | 19.03895187 | 20.3042984 | 20.24493027 |
| O75369-2 | FLNB_HUMAN Isoform 2 of Filamin-B OS | 23.54580688 | 23.33183289 | 23.51544952 | 23.3334713 |
| B4DJV2 | B4DJV2_HUMAN Citrate synthase OS | 20.92810249 | 20.59532356 | 20.07365608 | 21.55524826 |
| O75396 | SC22B_HUMAN Vesicle-trafficking protein SEC22b OS | 21.74324417 | 21.7069149 | 21.60792542 | 20.76983452 |
| O76003 | GLRX3_HUMAN Glutaredoxin-3 OS | 20.27491951 | 19.94077301 | 18.5617981 | 20.08108902 |
| O94979-3 | SC31A_HUMAN Isoform 3 of Protein transport protein Sec31A OS | 20.96845818 | 21.05278206 | 20.82539749 | 20.87927246 |
| O95373 | IPO7_HUMAN Importin-7 OS | 20.65709114 | 20.65333557 | 21.17102814 | 20.54726791 |
| O95782-2 | AP2A1_HUMAN Isoform B of AP-2 complex subunit alpha-1 OS | 19.28873253 | 18.351408 | 18.48805237 | 19.44828987 |
| O95816 | BAG2_HUMAN BAG family molecular chaperone regulator 2 OS | 19.18461418 | 19.14443588 | 19.68760109 | 19.34550858 |
| P00338 | LDHA_HUMAN L-lactate dehydrogenase A chain OS | 25.44344711 | 25.19159508 | 25.05527687 | 25.45575714 |
| P00367 | DHE3_HUMAN Glutamate dehydrogenase 1, mitochondrial OS | 20.72550583 | 20.42521667 | 20.81932831 | 20.7519722 |
| P00387-2 | NB5R3_HUMAN Isoform 2 of NADH-cytochrome b5 reductase 3 OS | 21.65530205 | 21.77398491 | 21.40265274 | 22.45176506 |
| P00403 | COX2_HUMAN Cytochrome c oxidase subunit 2 OS | 20.24376869 | 18.19075394 | 20.23958015 | 18.82337379 |
| P00505-2 | AATM_HUMAN Isoform 2 of Aspartate aminotransferase, mitochondrial OS | 20.64368057 | 20.42624092 | 19.4458847 | 19.96225739 |
| P00558 | PGK1_HUMAN Phosphoglycerate kinase 1 OS | 24.08772278 | 23.93939209 | 23.55262375 | 23.79451561 |
| P00568 | KAD1_HUMAN Adenylate kinase isoenzyme 1 OS | 19.53301811 | 20.11629295 | 19.46778107 | 18.75499535 |
| P02452 | CO1A1_HUMAN Collagen alpha-1(I) chain OS | 23.09102058 | 23.28248978 | 23.32072258 | 22.52114105 |
| P02545 | LMNA_HUMAN Prelamin-A/C OS | 25.78877831 | 25.64861488 | 25.65376091 | 25.40311432 |
| P02751-5 | FINC_HUMAN Isoform 5 of Fibronectin OS | 19.21175766 | 20.52219391 | 21.38642883 | 19.76073647 |
| P02786 | TFR1_HUMAN Transferrin receptor protein 1 OS | 19.10517883 | 20.29356194 | 20.46383476 | 18.64682007 |
| P04075 | ALDOA_HUMAN Fructose-bisphosphate aldolase A OS | 25.22262001 | 25.09051132 | 25.17108154 | 25.27159691 |
| P04080 | CYTB_HUMAN Cystatin-B OS | 20.52611732 | 19.50829697 | 20.25234222 | 20.95091438 |
| P04083 | ANXA1_HUMAN Annexin A1 OS | 25.49655724 | 25.25198174 | 25.19745636 | 25.76468658 |
| P04181 | OAT_HUMAN Ornithine aminotransferase, mitochondrial OS | 19.78298378 | 18.27453041 | 19.64839745 | 19.92171097 |
| P04350 | TBB4A_HUMAN Tubulin beta-4A chain OS | 19.22675705 | 18.80141258 | 20.51595306 | 19.99050903 |
| P04406 | G3P_HUMAN Glyceraldehyde-3-phosphate dehydrogenase OS | 25.86968994 | 25.795681 | 25.68059349 | 26.01186371 |
| K7EM73 | K7EM73_HUMAN Calcium-activated neutral proteinase small subunit (Fragment) OS | 20.34028053 | 21.09706306 | 20.18728256 | 21.22464561 |
| A0A6Q8PFK8 | A0A6Q8PFK8_HUMAN Heat shock protein beta-1 OS | 22.90451813 | 23.41905403 | 22.59400177 | 24.08909798 |
| P04843 | RPN1_HUMAN Dolichyl-diphosphooligosaccharide--protein glycosyltransferase subunit 1 OS | 22.59996033 | 22.73931503 | 22.67497635 | 22.67910576 |
| P04844-2 | RPN2_HUMAN Isoform 2 of Dolichyl-diphosphooligosaccharide--protein glycosyltransferase subunit 2 OS | 21.00449753 | 21.7910614 | 21.34098625 | 21.54443932 |
| P05023-3 | AT1A1_HUMAN Isoform 3 of Sodium/potassium-transporting ATPase subunit alpha-1 OS | 21.17102814 | 18.53874207 | 20.76530838 | 19.07042313 |
| P05141 | ADT2_HUMAN ADP/ATP translocase 2 OS | 22.5421505 | 22.79127884 | 22.61236572 | 22.96227455 |
| P05387 | RLA2_HUMAN 60S acidic ribosomal protein P2 OS | 20.0263443 | 19.84896278 | 19.8745079 | 20.33527184 |
| P05388 | RLA0_HUMAN 60S acidic ribosomal protein P0 OS | 22.12786102 | 23.20111084 | 23.83614922 | 22.81395721 |
| P05455 | LA_HUMAN Lupus La protein OS | 20.18555832 | 20.52085304 | 18.54012489 | 20.43267632 |
| H7C4K3 | H7C4K3_HUMAN Integrin beta-1 OS | 22.45108604 | 22.80725098 | 23.01367569 | 22.93646622 |
| P06576 | ATPB_HUMAN ATP synthase subunit beta, mitochondrial OS | 22.97744751 | 23.98988533 | 23.63433647 | 23.94476318 |
| R4GN98 | R4GN98_HUMAN Protein S100 (Fragment) OS | 20.53221893 | 22.26570702 | 22.08384895 | 20.79827881 |
| P06733 | ENOA_HUMAN Alpha-enolase OS | 26.81433296 | 26.25585747 | 25.92379379 | 26.77341652 |
| P06744 | G6PI_HUMAN Glucose-6-phosphate isomerase OS | 21.22564507 | 21.50329399 | 22.13433075 | 21.45317268 |
| P06748-3 | NPM_HUMAN Isoform 3 of Nucleophosmin OS | 23.91488075 | 23.17753601 | 23.01529312 | 23.60625458 |
| P06753-2 | TPM3_HUMAN Isoform 2 of Tropomyosin alpha-3 chain OS | 24.84282875 | 25.24285316 | 24.69620132 | 24.69109726 |
| P07195 | LDHB_HUMAN L-lactate dehydrogenase B chain OS | 23.87483406 | 24.55174446 | 24.20015335 | 24.21489716 |
| P07237 | PDIA1_HUMAN Protein disulfide-isomerase OS | 23.87389565 | 24.38929367 | 24.5389576 | 24.61605072 |
| P07355 | ANXA2_HUMAN Annexin A2 OS | 26.3249855 | 26.16036797 | 26.40963364 | 26.59448624 |
| Q5JP53 | Q5JP53_HUMAN Tubulin beta chain OS | 25.56784439 | 25.24590302 | 24.97814751 | 24.51701164 |
| P07737 | PROF1_HUMAN Profilin-1 OS | 25.31572151 | 24.98984146 | 24.69280052 | 25.51267815 |
| P07741-2 | APT_HUMAN Isoform 2 of Adenine phosphoribosyltransferase OS | 19.49337769 | 19.55965996 | 19.41886902 | 19.26836014 |
| P07814 | SYEP_HUMAN Bifunctional glutamate/proline--tRNA ligase OS | 20.97729111 | 20.52209854 | 20.81158829 | 20.95496559 |
| A0A7I2V668 | A0A7I2V668_HUMAN Cathepsin B OS | 18.59151268 | 19.28309441 | 18.92534637 | 20.0256691 |
| P07900 | HS90A_HUMAN Heat shock protein HSP 90-alpha OS | 24.97701073 | 24.96798325 | 24.85262489 | 24.31568718 |
| P07954-2 | FUMH_HUMAN Isoform Cytoplasmic of Fumarate hydratase, mitochondrial OS | 21.47427177 | 19.83877563 | 18.75895691 | 20.33145142 |
| A0A087WTA8 | A0A087WTA8_HUMAN Collagen alpha-2(I) chain OS | 19.50655174 | 20.52200317 | 20.08925819 | 18.34784698 |
| P08133-2 | ANXA6_HUMAN Isoform 2 of Annexin A6 OS | 21.31440163 | 20.74706268 | 22.89498711 | 20.61063194 |
| P08195-2 | 4F2_HUMAN Isoform 2 of 4F2 cell-surface antigen heavy chain OS | 20.74369621 | 20.62631416 | 20.87252426 | 21.52616501 |
| P08238 | HS90B_HUMAN Heat shock protein HSP 90-beta OS | 26.66275215 | 26.74239731 | 26.63740921 | 26.3826313 |
| P08670 | VIME_HUMAN Vimentin OS | 28.09763145 | 28.45575714 | 28.18338203 | 28.07418633 |
| P08708 | RS17_HUMAN 40S ribosomal protein S17 OS | 20.42121506 | 20.89081955 | 19.0922699 | 20.2166748 |
| P08758 | ANXA5_HUMAN Annexin A5 OS | 26.03416443 | 25.64617157 | 25.64603424 | 26.01497269 |
| C9J9K3 | C9J9K3_HUMAN 40S ribosomal protein SA (Fragment) OS | 23.82997131 | 23.51689148 | 23.25066566 | 23.94556618 |
| P09211 | GSTP1_HUMAN Glutathione S-transferase P OS | 23.67152405 | 23.38508606 | 23.45989037 | 23.40210533 |
| P09382 | LEG1_HUMAN Galectin-1 OS | 25.39008141 | 24.85357666 | 24.82914734 | 25.43990135 |
| P09493-3 | TPM1_HUMAN Isoform 3 of Tropomyosin alpha-1 chain OS | 23.12140846 | 23.01855659 | 22.72708702 | 23.17525291 |
| P09651-3 | ROA1_HUMAN Isoform 2 of Heterogeneous nuclear ribonucleoprotein A1 OS | 23.24212646 | 23.56790352 | 23.55882454 | 23.16250992 |
| P09936 | UCHL1_HUMAN Ubiquitin carboxyl-terminal hydrolase isozyme L1 OS | 21.78048706 | 21.51016998 | 21.51527977 | 21.37081909 |
| J3QS39 | J3QS39_HUMAN Polyubiquitin-B (Fragment) OS | 25.05804634 | 24.82701683 | 24.93737221 | 25.38742256 |
| P0DMV8-2 | HS71A_HUMAN Isoform 2 of Heat shock 70 kDa protein 1A OS | 22.42190742 | 22.63862991 | 22.50331879 | 22.5267849 |
| P0DP25 | CALM3_HUMAN Calmodulin-3 OS | 21.64552879 | 21.67882729 | 21.98693848 | 21.82629013 |
| P10599 | THIO_HUMAN Thioredoxin OS | 23.13769531 | 22.60306549 | 22.52657127 | 22.82549286 |
| A0A7I2V599 | A0A7I2V599_HUMAN 60 kDa heat shock protein, mitochondrial OS | 23.39479828 | 23.44733238 | 24.19151878 | 24.08172226 |
| P11021 | BIP_HUMAN Endoplasmic reticulum chaperone BiP OS | 25.55800819 | 25.57282639 | 25.71832085 | 25.92592239 |
| P11142 | HSP7C_HUMAN Heat shock cognate 71 kDa protein OS | 26.54789734 | 26.72455406 | 26.61568642 | 26.60724258 |
| P11279 | LAMP1_HUMAN Lysosome-associated membrane glycoprotein 1 OS | 17.32139587 | 19.31709862 | 19.2150135 | 20.2681942 |
| P11413 | G6PD_HUMAN Glucose-6-phosphate 1-dehydrogenase OS | 23.88892555 | 23.74745178 | 23.55168533 | 23.64735222 |
| V9GYY3 | V9GYY3_HUMAN C-1-tetrahydrofolate synthase, cytoplasmic OS | 19.85201454 | 20.60909843 | 17.56593513 | 20.80475235 |
| P11940-2 | PABP1_HUMAN Isoform 2 of Polyadenylate-binding protein 1 OS | 21.51590538 | 22.08718872 | 22.50006294 | 22.46543121 |
| P12004 | PCNA_HUMAN Proliferating cell nuclear antigen OS | 20.3467865 | 18.33042145 | 19.56888008 | 20.10712433 |
| A0A087X0S5 | A0A087X0S5_HUMAN Collagen alpha-1(VI) chain OS | 18.71038246 | 20.07195473 | 20.02998734 | 19.10149193 |
| P12110-3 | CO6A2_HUMAN Isoform 2C2A of Collagen alpha-2(VI) chain OS | 18.38016319 | 18.76461792 | 19.70730209 | 18.65681839 |
| P12111-2 | CO6A3_HUMAN Isoform 2 of Collagen alpha-3(VI) chain OS | 20.01154327 | 18.73948097 | 18.68630219 | 19.30571556 |
| P12236 | ADT3_HUMAN ADP/ATP translocase 3 OS | 21.83865166 | 21.45498085 | 21.2160244 | 21.11667442 |
| E7ETK5 | E7ETK5_HUMAN Inosine-5-monophosphate dehydrogenase 2 OS | 20.26739502 | 19.94535255 | 20.17840195 | 20.36463547 |
| P12814 | ACTN1_HUMAN Alpha-actinin-1 OS | 24.16867256 | 23.90655708 | 24.06923676 | 24.06767845 |
| P12956 | XRCC6_HUMAN X-ray repair cross-complementing protein 6 OS | 22.48537064 | 22.75951004 | 21.96677017 | 22.26014709 |
| P13010 | XRCC5_HUMAN X-ray repair cross-complementing protein 5 OS | 20.98161697 | 21.04693413 | 18.7273922 | 21.02418137 |
| P13489 | RINI_HUMAN Ribonuclease inhibitor OS | 19.07761192 | 19.36923981 | 20.75564384 | 21.56532478 |
| P13639 | EF2_HUMAN Elongation factor 2 OS | 25.42521667 | 25.36131859 | 25.49347496 | 25.31458092 |
| P13667 | PDIA4_HUMAN Protein disulfide-isomerase A4 OS | 22.20329285 | 21.50198364 | 22.4616394 | 22.20401001 |
| P13693 | TCTP_HUMAN Translationally-controlled tumor protein OS | 22.94494057 | 23.02570343 | 22.74299812 | 21.68440247 |
| P13797-3 | PLST_HUMAN Isoform 3 of Plastin-3 OS | 20.80325508 | 21.41012573 | 20.93984032 | 21.11914635 |
| P14314-2 | GLU2B_HUMAN Isoform 2 of Glucosidase 2 subunit beta OS | 20.49462891 | 20.55646515 | 20.94670868 | 20.54952431 |
| P14618 | KPYM_HUMAN Pyruvate kinase PKM OS | 26.33880234 | 26.30679893 | 26.95038033 | 26.92150307 |
| P14625 | ENPL_HUMAN Endoplasmin OS | 25.14616013 | 24.98215675 | 25.34106064 | 25.01962471 |
| P14868-2 | SYDC_HUMAN Isoform 2 of Aspartate--tRNA ligase, cytoplasmic OS | 20.75808716 | 20.91320229 | 20.63944626 | 20.86052704 |
| P15121 | ALDR_HUMAN Aldo-keto reductase family 1 member B1 OS | 19.60351372 | 20.02106667 | 20.2934494 | 19.91615105 |
| P15170-2 | ERF3A_HUMAN Isoform 2 of Eukaryotic peptide chain release factor GTP-binding subunit ERF3A OS | 18.81366158 | 18.34658432 | 19.01454353 | 19.65240097 |
| E7EQR4 | E7EQR4_HUMAN Ezrin OS | 21.33494568 | 20.75148201 | 21.29288864 | 21.32827568 |
| P15531 | NDKA_HUMAN Nucleoside diphosphate kinase A OS | 20.29949951 | 21.05039406 | 21.42157364 | 20.32256699 |
| B4DLR8 | B4DLR8_HUMAN NAD(P)H dehydrogenase [quinone] 1 OS | 21.96476173 | 22.27486229 | 21.60747528 | 22.14973068 |
| P15880 | RS2_HUMAN 40S ribosomal protein S2 OS | 21.47847557 | 21.86067772 | 21.50489426 | 20.85094643 |
| H0YD13 | H0YD13_HUMAN CD44 antigen OS | 21.84796906 | 21.62055397 | 22.34597397 | 21.94706535 |
| P16152 | CBR1_HUMAN Carbonyl reductase [NADPH] 1 OS | 20.68893242 | 20.65210915 | 20.29670143 | 21.14008522 |
| P16401 | H15_HUMAN Histone H1.5 OS | 20.50445747 | 21.03650856 | 19.98509789 | 20.09106827 |
| P16403 | H12_HUMAN Histone H1.2 OS | 22.56239319 | 23.6404171 | 22.6115551 | 22.15610313 |
| A2A2D0 | A2A2D0_HUMAN Stathmin (Fragment) OS | 22.06766129 | 22.17063141 | 21.99043846 | 22.18546677 |
| P17066 | HSP76_HUMAN Heat shock 70 kDa protein 6 OS | 23.97018051 | 23.79114151 | 24.3663311 | 18.36454964 |
| P17301 | ITA2_HUMAN Integrin alpha-2 OS | 20.62533379 | 18.01978874 | 20.27264404 | 19.34575462 |
| P17844-2 | DDX5_HUMAN Isoform 2 of Probable ATP-dependent RNA helicase DDX5 OS | 21.91779709 | 21.96204567 | 22.16063309 | 22.15893936 |
| P17931 | LEG3_HUMAN Galectin-3 OS | 22.04069519 | 21.6466713 | 21.1992836 | 21.85870743 |
| P17987 | TCPA_HUMAN T-complex protein 1 subunit alpha OS | 21.94663811 | 20.91830635 | 22.14678192 | 22.09316635 |
| P18077 | RL35A_HUMAN 60S ribosomal protein L35a OS | 21.23876381 | 21.20012283 | 20.94927597 | 20.95183945 |
| P18085 | ARF4_HUMAN ADP-ribosylation factor 4 OS | 22.76761436 | 23.49240494 | 22.76050568 | 22.95882797 |
| P18124 | RL7_HUMAN 60S ribosomal protein L7 OS | 22.55255318 | 22.32608223 | 22.11359406 | 22.20711136 |
| P18206-2 | VINC_HUMAN Isoform 1 of Vinculin OS | 25.51270866 | 25.16748619 | 25.25767517 | 25.19121742 |
| A0A087WXM6 | A0A087WXM6_HUMAN 60S ribosomal protein L17 (Fragment) OS | 22.04683495 | 21.91356659 | 21.91914368 | 21.42792892 |
| P18669 | PGAM1_HUMAN Phosphoglycerate mutase 1 OS | 21.50450706 | 22.3384037 | 18.76493454 | 22.02894402 |
| A0A7I2V5M5 | A0A7I2V5M5_HUMAN Nucleolin OS | 23.5639534 | 23.83402824 | 24.17607498 | 23.00643349 |
| P20340-2 | RAB6A_HUMAN Isoform 2 of Ras-related protein Rab-6A OS | 20.68260384 | 20.27082062 | 19.90782928 | 18.33300209 |
| P20618 | PSB1_HUMAN Proteasome subunit beta type-1 OS | 21.38637543 | 20.9909935 | 20.79558563 | 20.46821785 |
| E9PP21 | E9PP21_HUMAN Cysteine and glycine-rich protein 1 OS | 22.33764267 | 22.17621231 | 21.712677 | 22.27121925 |
| P21333-2 | FLNA_HUMAN Isoform 2 of Filamin-A OS | 27.50074005 | 27.26731682 | 27.49845886 | 27.23908806 |
| P21796 | VDAC1_HUMAN Voltage-dependent anion-selective channel protein 1 OS | 20.80317497 | 21.00257683 | 19.18766403 | 21.32289696 |
| P21980 | TGM2_HUMAN Protein-glutamine gamma-glutamyltransferase 2 OS | 21.63706017 | 20.52859879 | 21.82080841 | 21.45973969 |
| P22234 | PUR6_HUMAN Multifunctional protein ADE2 OS | 20.32586288 | 18.53541374 | 18.37940216 | 19.8512516 |
| P22307-6 | SCP2_HUMAN Isoform 6 of Sterol carrier protein 2 OS | 19.93051529 | 18.27853394 | 19.50288582 | 20.16041756 |
| P22314-2 | UBA1_HUMAN Isoform 2 of Ubiquitin-like modifier-activating enzyme 1 OS | 23.46874046 | 23.45148849 | 23.38613892 | 23.43224335 |
| P22392-2 | NDKB_HUMAN Isoform 3 of Nucleoside diphosphate kinase B OS | 24.95557785 | 24.43020248 | 24.24285316 | 24.82561111 |
| A0A7I2V4I6 | A0A7I2V4I6_HUMAN Heterogeneous nuclear ribonucleoproteins A2/B1 OS | 22.79590416 | 22.79405975 | 22.6634903 | 22.38616562 |
| A0A2R8YDM0 | A0A2R8YDM0_HUMAN Prostaglandin-endoperoxide synthase (Fragment) OS | 19.32447243 | 19.92104149 | 20.32586288 | 20.453022 |
| P23246 | SFPQ_HUMAN Splicing factor, proline- and glutamine-rich OS | 21.78572464 | 22.33884048 | 22.07407951 | 19.62455559 |
| P23284 | PPIB_HUMAN Peptidyl-prolyl cis-trans isomerase B OS | 24.42136765 | 23.90334129 | 23.9958477 | 23.69242859 |
| P23381-2 | SYWC_HUMAN Isoform 2 of Tryptophan--tRNA ligase, cytoplasmic OS | 18.65694618 | 18.98865128 | 18.03157043 | 18.45745659 |
| P23396 | RS3_HUMAN 40S ribosomal protein S3 OS | 23.78096771 | 23.62287903 | 23.62053299 | 23.94708443 |
| P23526 | SAHH_HUMAN Adenosylhomocysteinase OS | 21.50300217 | 21.42700768 | 21.16337013 | 21.25948524 |
| P23528 | COF1_HUMAN Cofilin-1 OS | 24.93597984 | 24.95938683 | 24.94060135 | 25.63765717 |
| P23634-7 | AT2B4_HUMAN Isoform ZB of Plasma membrane calcium-transporting ATPase 4 OS | 20.08498573 | 20.69200325 | 20.50794601 | 18.06524467 |
| P24534 | EF1B_HUMAN Elongation factor 1-beta OS | 22.30502129 | 21.72263336 | 22.14027405 | 22.05857468 |
| Q5QNZ2 | Q5QNZ2_HUMAN ATP synthase F(0) complex subunit B1, mitochondrial OS | 19.32291794 | 20.23958015 | 19.95787239 | 19.57420921 |
| P25398 | RS12_HUMAN 40S ribosomal protein S12 OS | 22.16864204 | 21.38205338 | 21.39037132 | 20.97330284 |
| P25705 | ATPA_HUMAN ATP synthase subunit alpha, mitochondrial OS | 24.05150604 | 24.12273598 | 24.0110321 | 23.74488831 |
| F5GX11 | F5GX11_HUMAN Proteasome subunit alpha type-1 OS | 21.36121178 | 20.17657661 | 20.6032238 | 20.54387474 |
| A0A7I2V2H3 | A0A7I2V2H3_HUMAN UPF0415 protein C7orf25 OS | 20.29299927 | 20.44949913 | 18.5714798 | 17.94278336 |
| P25788-2 | PSA3_HUMAN Isoform 2 of Proteasome subunit alpha type-3 OS | 19.95400429 | 19.35004044 | 18.62101173 | 20.80829239 |
| H0YMZ1 | H0YMZ1_HUMAN Proteasome subunit alpha type (Fragment) OS | 20.44818878 | 18.83518982 | 20.33745193 | 20.11667442 |
| P26038 | MOES_HUMAN Moesin OS | 24.08448219 | 24.15236282 | 24.07667542 | 23.99576187 |
| P26373 | RL13_HUMAN 60S ribosomal protein L13 OS | 21.72088051 | 22.07162666 | 21.93329811 | 22.05596352 |
| A6NLN1 | A6NLN1_HUMAN Polypyrimidine tract-binding protein 1 OS | 21.35498619 | 22.10261536 | 21.91195869 | 22.54333115 |
| P26639 | SYTC_HUMAN Threonine--tRNA ligase 1, cytoplasmic OS | 18.68282509 | 18.14849663 | 20.6827755 | 20.56978226 |
| P26640 | SYVC_HUMAN Valine--tRNA ligase OS | 20.64491272 | 20.36900902 | 19.86188889 | 20.05152321 |
| P26641 | EF1G_HUMAN Elongation factor 1-gamma OS | 24.44909668 | 23.74570847 | 23.42226791 | 23.98589706 |
| P27348 | 1433T_HUMAN 14-3-3 protein theta OS | 21.95311928 | 21.91830635 | 22.69824791 | 21.8007679 |
| F8W7C6 | F8W7C6_HUMAN 60S ribosomal protein L10 OS | 19.95446968 | 20.85041237 | 20.13808632 | 20.4403019 |
| P27797 | CALR_HUMAN Calreticulin OS | 24.27582932 | 24.20321846 | 24.16361618 | 24.06488609 |
| P27816-6 | MAP4_HUMAN Isoform 6 of Microtubule-associated protein 4 OS | 22.30866432 | 22.13295174 | 21.96705246 | 22.24091911 |
| P27824 | CALX_HUMAN Calnexin OS | 22.58545494 | 22.43690109 | 22.57588577 | 22.74349213 |
| P28066 | PSA5_HUMAN Proteasome subunit alpha type-5 OS | 19.54440308 | 21.35697365 | 18.90184212 | 22.0509243 |
| P28072 | PSB6_HUMAN Proteasome subunit beta type-6 OS | 20.35412407 | 20.73537445 | 20.70480537 | 20.0171299 |
| P28074 | PSB5_HUMAN Proteasome subunit beta type-5 OS | 20.54820824 | 20.92918587 | 21.03838539 | 18.24308014 |
| P29373 | RABP2_HUMAN Cellular retinoic acid-binding protein 2 OS | 21.69132042 | 21.58641624 | 21.46218872 | 21.75605202 |
| P29401 | TKT_HUMAN Transketolase OS | 23.89309883 | 23.9358902 | 23.79431725 | 24.02934837 |
| E9PK01 | E9PK01_HUMAN Elongation factor 1-delta (Fragment) OS | 23.84028625 | 24.22140312 | 23.92243195 | 23.40859795 |
| P29966 | MARCS_HUMAN Myristoylated alanine-rich C-kinase substrate OS | 21.43135071 | 21.26441956 | 21.41369438 | 20.4724884 |
| P30040 | ERP29_HUMAN Endoplasmic reticulum resident protein 29 OS | 20.24875641 | 19.04772758 | 19.87669754 | 20.63280678 |
| P30041 | PRDX6_HUMAN Peroxiredoxin-6 OS | 22.81677246 | 22.49831009 | 21.80408287 | 23.03259659 |
| P30044-2 | PRDX5_HUMAN Isoform Cytoplasmic+peroxisomal of Peroxiredoxin-5, mitochondrial OS | 20.55496788 | 19.9026432 | 20.01576805 | 20.35175514 |
| P30048-2 | PRDX3_HUMAN Isoform 2 of Thioredoxin-dependent peroxide reductase, mitochondrial OS | 20.88323021 | 20.84053421 | 20.80207062 | 21.22311401 |
| P30050 | RL12_HUMAN 60S ribosomal protein L12 OS | 22.99602127 | 22.84628677 | 22.77017784 | 23.10616684 |
| P30086 | PEBP1_HUMAN Phosphatidylethanolamine-binding protein 1 OS | 21.17937469 | 20.88226128 | 21.31118965 | 20.51691628 |
| P30101 | PDIA3_HUMAN Protein disulfide-isomerase A3 OS | 24.33892059 | 24.46351051 | 24.36833 | 24.09595299 |
| P30153 | 2AAA_HUMAN Serine/threonine-protein phosphatase 2A 65 kDa regulatory subunit A alpha isoform OS | 20.17438507 | 17.96764565 | 20.35347939 | 20.97889709 |
| P31153-2 | METK2_HUMAN Isoform 2 of S-adenosylmethionine synthase isoform type-2 OS | 21.7015934 | 21.30752563 | 18.02231789 | 19.35296822 |
| P31939 | PUR9_HUMAN Bifunctional purine biosynthesis protein ATIC OS | 19.96395111 | 20.33712578 | 19.75433922 | 19.82289505 |
| P31943 | HNRH1_HUMAN Heterogeneous nuclear ribonucleoprotein H OS | 22.09732056 | 21.65294075 | 21.94141769 | 21.81796265 |
| P31946-2 | 1433B_HUMAN Isoform Short of 14-3-3 protein beta/alpha OS | 21.12395287 | 22.44609451 | 22.75191116 | 21.69510841 |
| P31948 | STIP1_HUMAN Stress-induced-phosphoprotein 1 OS | 21.68101692 | 21.71586227 | 21.76862335 | 21.52042198 |
| P32119 | PRDX2_HUMAN Peroxiredoxin-2 OS | 21.18083191 | 21.54491234 | 21.79018593 | 21.64059448 |
| D6RAN4 | D6RAN4_HUMAN 60S ribosomal protein L9 (Fragment) OS | 21.75902176 | 21.75474739 | 21.77499008 | 21.77527046 |
| P33176 | KINH_HUMAN Kinesin-1 heavy chain OS | 21.85064125 | 21.33472633 | 21.40135002 | 18.63724709 |
| H0YMM5 | H0YMM5_HUMAN Deoxyuridine 5-triphosphate nucleotidohydrolase OS | 17.98854065 | 19.09668159 | 18.96603775 | 18.76483917 |
| P34932 | HSP74_HUMAN Heat shock 70 kDa protein 4 OS | 20.94005585 | 21.18058968 | 20.53164864 | 20.7768383 |
| C9J0J7 | C9J0J7_HUMAN Profilin OS | 20.86014748 | 20.92998123 | 21.36666298 | 18.95683861 |
| G3XAM7 | G3XAM7_HUMAN Catenin (Cadherin-associated protein), alpha 1, 102kDa, isoform CRA_a OS | 18.23778915 | 19.61126328 | 18.54493904 | 20.20490456 |
| P35232 | PHB_HUMAN Prohibitin OS | 21.47288513 | 21.34759712 | 21.18289375 | 21.02654839 |
| P35241 | RADI_HUMAN Radixin OS | 18.7151165 | 18.15534973 | 19.38460732 | 18.39462662 |
| K7EP65 | K7EP65_HUMAN 60S ribosomal protein L22 (Fragment) OS | 21.8465538 | 22.31024742 | 21.9591465 | 22.57124138 |
| P35579 | MYH9_HUMAN Myosin-9 OS | 27.21946526 | 27.34975815 | 27.44679451 | 27.2035923 |
| P35580 | MYH10_HUMAN Myosin-10 OS | 19.46911812 | 18.07261276 | 19.93718338 | 19.44395065 |
| P35637-2 | FUS_HUMAN Isoform Short of RNA-binding protein FUS OS | 20.26109314 | 20.34527016 | 20.40010071 | 19.83422089 |
| C9JX88 | C9JX88_HUMAN 26S proteasome regulatory subunit 7 OS | 20.30295944 | 18.80281448 | 19.17874146 | 20.59313583 |
| P36578 | RL4_HUMAN 60S ribosomal protein L4 OS | 23.30841446 | 23.01481628 | 22.90056229 | 22.87282562 |
| P37802 | TAGL2_HUMAN Transgelin-2 OS | 22.95617104 | 23.49093819 | 23.52838516 | 22.38687515 |
| P37837 | TALDO_HUMAN Transaldolase OS | 21.21501541 | 21.01120186 | 20.64121246 | 19.90301132 |
| A0A7I2V2G2 | A0A7I2V2G2_HUMAN Stress-70 protein, mitochondrial OS | 22.65066528 | 23.09741592 | 22.98363686 | 23.06202126 |
| P39019 | RS19_HUMAN 40S ribosomal protein S19 OS | 22.94916916 | 23.27696419 | 23.08090973 | 23.17503929 |
| P39023 | RL3_HUMAN 60S ribosomal protein L3 OS | 22.28551292 | 21.49066925 | 21.54707909 | 21.69935036 |
| P39656-3 | OST48_HUMAN Isoform 3 of Dolichyl-diphosphooligosaccharide--protein glycosyltransferase 48 kDa subunit OS | 21.70602798 | 20.82772446 | 21.10014725 | 21.47649956 |
| P40227 | TCPZ_HUMAN T-complex protein 1 subunit zeta OS | 22.43456078 | 22.74435425 | 22.52492142 | 22.80258369 |
| A0A7I2V2L9 | A0A7I2V2L9_HUMAN 60S ribosomal protein L13a OS | 22.31454086 | 22.17834091 | 22.05029297 | 22.66062164 |
| P40925 | MDHC_HUMAN Malate dehydrogenase, cytoplasmic OS | 18.32060432 | 19.06594086 | 20.32597351 | 20.0188961 |
| P40926 | MDHM_HUMAN Malate dehydrogenase, mitochondrial OS | 22.7376442 | 22.84955406 | 22.19084167 | 23.05339622 |
| P40939 | ECHA_HUMAN Trifunctional enzyme subunit alpha, mitochondrial OS | 20.05629349 | 18.65982819 | 18.91164589 | 20.71280289 |
| P41091 | IF2G_HUMAN Eukaryotic translation initiation factor 2 subunit 3 OS | 19.02732849 | 20.92303085 | 20.30374146 | 20.31052399 |
| A0A6Q8PGW4 | A0A6Q8PGW4_HUMAN Diadenosine tetraphosphate synthetase OS | 22.65839958 | 20.50096321 | 22.15035057 | 22.33134079 |
| A0A0A0MSX9 | A0A0A0MSX9_HUMAN Isoleucyl-tRNA synthetase OS | 20.78803635 | 20.23233604 | 18.30582237 | 19.70235252 |
| P42677 | RS27_HUMAN 40S ribosomal protein S27 OS | 21.05291557 | 21.36292458 | 21.74365616 | 21.24103546 |
| P42704 | LPPRC_HUMAN Leucine-rich PPR motif-containing protein, mitochondrial OS | 20.07887459 | 20.15930939 | 19.95375824 | 19.05296326 |
| P42766 | RL35_HUMAN 60S ribosomal protein L35 OS | 20.98530579 | 21.26436234 | 20.98300934 | 20.2877121 |
| D6REM6 | D6REM6_HUMAN Matrin-3 OS | 18.12245369 | 20.94327927 | 20.88807106 | 18.25110435 |
| C9J3L8 | C9J3L8_HUMAN Signal sequence receptor subunit alpha OS | 20.83352661 | 20.42552376 | 19.78462219 | 20.82826614 |
| P43487-2 | RANG_HUMAN Isoform 2 of Ran-specific GTPase-activating protein OS | 19.92619133 | 21.06137848 | 18.36230278 | 20.40851974 |
| A0A0A0MR02 | A0A0A0MR02_HUMAN Outer mitochondrial membrane protein porin 2 (Fragment) OS | 18.93975449 | 18.82051849 | 18.63635254 | 20.91597557 |
| P46060 | RAGP1_HUMAN Ran GTPase-activating protein 1 OS | 20.7369442 | 20.84919167 | 20.60918999 | 20.90771294 |
| E9PLL6 | E9PLL6_HUMAN 60S ribosomal protein L27a OS | 20.7542572 | 19.02553749 | 18.59032059 | 20.97994423 |
| A0A2R8Y6J3 | A0A2R8Y6J3_HUMAN 60S ribosomal protein L5 (Fragment) OS | 21.16484261 | 20.87919807 | 21.56648636 | 20.66553116 |
| P46779 | RL28_HUMAN 60S ribosomal protein L28 OS | 20.38247681 | 21.68071747 | 22.26384735 | 20.03965759 |
| P46781 | RS9_HUMAN 40S ribosomal protein S9 OS | 22.76727104 | 23.10210228 | 22.92795753 | 22.23549461 |
| P46783 | RS10_HUMAN 40S ribosomal protein S10 OS | 22.1238575 | 21.65560722 | 21.62880898 | 22.79249191 |
| P46821 | MAP1B_HUMAN Microtubule-associated protein 1B OS | 23.13908577 | 22.94111252 | 23.00273132 | 23.0926342 |
| P46940 | IQGA1_HUMAN Ras GTPase-activating-like protein IQGAP1 OS | 24.00821304 | 23.67281914 | 23.76910782 | 23.76708794 |
| P47756-2 | CAPZB_HUMAN Isoform 2 of F-actin-capping protein subunit beta OS | 22.01294136 | 21.34342957 | 21.38758659 | 21.88550377 |
| P47914 | RL29_HUMAN 60S ribosomal protein L29 OS | 19.93747139 | 21.24417496 | 18.78913689 | 19.66221237 |
| P48047 | ATPO_HUMAN ATP synthase subunit O, mitochondrial OS | 20.76700783 | 20.39362144 | 20.49101067 | 20.20179939 |
| P48444 | COPD_HUMAN Coatomer subunit delta OS | 19.20639038 | 18.78091049 | 19.65928841 | 20.10495186 |
| P48643 | TCPE_HUMAN T-complex protein 1 subunit epsilon OS | 19.14135933 | 19.8029232 | 21.50741386 | 21.48978806 |
| P49207 | RL34_HUMAN 60S ribosomal protein L34 OS | 21.11050606 | 21.28133011 | 20.90763855 | 21.49770164 |
| P49257 | LMAN1_HUMAN Protein ERGIC-53 OS | 20.85946465 | 20.31052399 | 18.4568367 | 20.73015785 |
| P49327 | FAS_HUMAN Fatty acid synthase OS | 22.85325241 | 22.78761864 | 22.85325241 | 22.35124207 |
| P49368-2 | TCPG_HUMAN Isoform 2 of T-complex protein 1 subunit gamma OS | 20.64790154 | 22.1076355 | 22.11603928 | 22.52676201 |
| P49411 | EFTU_HUMAN Elongation factor Tu, mitochondrial OS | 20.77209282 | 21.30996895 | 21.29148293 | 21.49389648 |
| A0A6Q8PGR9 | A0A6Q8PGR9_HUMAN Alanine--tRNA ligase OS | 18.80595398 | 20.223526 | 20.52410889 | 20.24435043 |
| P49721 | PSB2_HUMAN Proteasome subunit beta type-2 OS | 20.19002724 | 18.74065781 | 19.61156845 | 18.52177048 |
| P49748-2 | ACADV_HUMAN Isoform 2 of Very long-chain specific acyl-CoA dehydrogenase, mitochondrial OS | 20.55730629 | 20.48492813 | 18.15488434 | 20.70100212 |
| P49755 | TMEDA_HUMAN Transmembrane emp24 domain-containing protein 10 OS | 21.40457535 | 20.70201683 | 20.59322739 | 21.44536209 |
| P50395 | GDIB_HUMAN Rab GDP dissociation inhibitor beta OS | 23.22912598 | 22.87820625 | 22.23777199 | 23.04550171 |
| P50454 | SERPH_HUMAN Serpin H1 OS | 25.6435585 | 25.03980064 | 24.48965263 | 25.09281158 |
| P50502 | F10A1_HUMAN Hsc70-interacting protein OS | 22.07234764 | 21.534832 | 20.55421829 | 21.27537346 |
| P50990 | TCPQ_HUMAN T-complex protein 1 subunit theta OS | 22.16060257 | 23.07182312 | 22.83300591 | 22.91832542 |
| P50991 | TCPD_HUMAN T-complex protein 1 subunit delta OS | 21.31318474 | 21.25112915 | 22.47221565 | 21.98971176 |
| P51148 | RAB5C_HUMAN Ras-related protein Rab-5C OS | 21.26080704 | 21.59514046 | 20.97379303 | 21.83047295 |
| P51149 | RAB7A_HUMAN Ras-related protein Rab-7a OS | 22.71793556 | 22.20158958 | 22.18401527 | 22.25715637 |
| P51571 | SSRD_HUMAN Translocon-associated protein subunit delta OS | 20.67676353 | 19.64981461 | 19.71739578 | 20.84383583 |
| P51572 | BAP31_HUMAN B-cell receptor-associated protein 31 OS | 17.92259598 | 18.25635529 | 19.50223923 | 19.21449471 |
| P52209-2 | 6PGD_HUMAN Isoform 2 of 6-phosphogluconate dehydrogenase, decarboxylating OS | 23.3372879 | 22.33946419 | 21.9394455 | 22.58217239 |
| P52272-2 | HNRPM_HUMAN Isoform 2 of Heterogeneous nuclear ribonucleoprotein M OS | 21.64072609 | 21.08628082 | 22.13599014 | 21.81710434 |
| J3KTF8 | J3KTF8_HUMAN Rho GDP-dissociation inhibitor 1 (Fragment) OS | 23.52133179 | 23.53872108 | 22.55695534 | 23.88874054 |
| P52907 | CAZA1_HUMAN F-actin-capping protein subunit alpha-1 OS | 22.35748482 | 21.97973442 | 22.09413338 | 22.56036568 |
| P53396-2 | ACLY_HUMAN Isoform 2 of ATP-citrate synthase OS | 21.96105766 | 21.92288589 | 22.14724731 | 22.02553368 |
| P53618 | COPB_HUMAN Coatomer subunit beta OS | 19.32070923 | 20.1977253 | 19.00530243 | 20.54434586 |
| P53621 | COPA_HUMAN Coatomer subunit alpha OS | 22.68382454 | 22.54750252 | 22.55025291 | 22.44899559 |
| P54136 | SYRC_HUMAN Arginine--tRNA ligase, cytoplasmic OS | 20.83776665 | 20.80009651 | 20.1692543 | 20.34559631 |
| P55060-4 | XPO2_HUMAN Isoform 4 of Exportin-2 OS | 19.82200813 | 18.66786385 | 17.65454102 | 19.97154999 |
| P55072 | TERA_HUMAN Transitional endoplasmic reticulum ATPase OS | 24.05705452 | 23.39139366 | 23.98771858 | 22.93711281 |
| P55084-2 | ECHB_HUMAN Isoform 2 of Trifunctional enzyme subunit beta, mitochondrial OS | 20.35067558 | 18.85640335 | 19.36937141 | 19.67572975 |
| H0YHC3 | H0YHC3_HUMAN Nucleosome assembly protein 1-like 1 (Fragment) OS | 20.18833733 | 20.7776413 | 20.59623337 | 17.94430542 |
| P55884 | EIF3B_HUMAN Eukaryotic translation initiation factor 3 subunit B OS | 18.57753944 | 19.83369637 | 19.02816963 | 20.20848274 |
| P59998 | ARPC4_HUMAN Actin-related protein 2/3 complex subunit 4 OS | 22.11470604 | 22.09377861 | 22.12316322 | 22.69166183 |
| P60174 | TPIS_HUMAN Triosephosphate isomerase OS | 23.27639771 | 23.84584618 | 24.09055138 | 24.59718895 |
| B3KW56 | B3KW56_HUMAN Eukaryotic translation initiation factor 3 subunit E OS | 19.91165161 | 19.76900673 | 18.54272652 | 20.17791557 |
| F8W1R7 | F8W1R7_HUMAN Myosin light polypeptide 6 OS | 23.86702919 | 24.01563263 | 23.8969841 | 24.06899071 |
| P60709 | ACTB_HUMAN Actin, cytoplasmic 1 OS | 29.5298996 | 29.50846863 | 29.72000885 | 29.53129578 |
| P60842 | IF4A1_HUMAN Eukaryotic initiation factor 4A-I OS | 25.29862595 | 24.65485382 | 24.5323143 | 24.97631264 |
| P60866 | RS20_HUMAN 40S ribosomal protein S20 OS | 22.93490028 | 23.04278374 | 22.17056847 | 22.09206963 |
| B1ALA9 | B1ALA9_HUMAN Ribose-phosphate pyrophosphokinase 1 OS | 19.31720924 | 18.96899223 | 19.94649506 | 19.62693977 |
| P60900 | PSA6_HUMAN Proteasome subunit alpha type-6 OS | 20.35820961 | 20.8303566 | 20.40883064 | 21.42152214 |
| P60953 | CDC42_HUMAN Cell division control protein 42 homolog OS | 20.50930023 | 20.43806839 | 20.9557457 | 20.54717255 |
| P60981 | DEST_HUMAN Destrin OS | 21.46418381 | 21.45563316 | 21.47516251 | 21.40431595 |
| P61019 | RAB2A_HUMAN Ras-related protein Rab-2A OS | 21.1293087 | 20.28093338 | 20.36324501 | 19.8890667 |
| P61088 | UBE2N_HUMAN Ubiquitin-conjugating enzyme E2 N OS | 20.9978981 | 21.51595306 | 21.42306328 | 21.61162376 |
| P61106 | RAB14_HUMAN Ras-related protein Rab-14 OS | 20.51431656 | 20.53525925 | 20.12325668 | 17.97779083 |
| B4DXW1 | B4DXW1_HUMAN Actin-like protein 3 OS | 22.71753693 | 21.74419022 | 21.8473568 | 22.20660591 |
| P61160 | ARP2_HUMAN Actin-related protein 2 OS | 22.2937851 | 21.52812195 | 22.10354424 | 21.49755478 |
| R4GMT0 | R4GMT0_HUMAN Alpha-centractin OS | 19.0608387 | 19.81391144 | 21.02890968 | 20.02242279 |
| P84077 | ARF1_HUMAN ADP-ribosylation factor 1 OS | 22.76844215 | 22.53793907 | 22.59048843 | 23.07713127 |
| P61224-2 | RAP1B_HUMAN Isoform 2 of Ras-related protein Rap-1b OS | 21.05834389 | 21.05940056 | 21.01678848 | 20.85574532 |
| P61247 | RS3A_HUMAN 40S ribosomal protein S3a OS | 21.88405037 | 22.07309914 | 22.11946297 | 21.52606964 |
| J3QRI7 | J3QRI7_HUMAN 60S ribosomal protein L26 (Fragment) OS | 21.34256172 | 21.26984978 | 21.22552681 | 21.58659935 |
| P61313 | RL15_HUMAN 60S ribosomal protein L15 OS | 19.74180603 | 21.89163589 | 21.21720695 | 19.95475388 |
| P61353 | RL27_HUMAN 60S ribosomal protein L27 OS | 22.93097305 | 22.72698212 | 22.68527985 | 22.77822304 |
| P61586 | RHOA_HUMAN Transforming protein RhoA OS | 20.54736137 | 20.57144928 | 20.46283722 | 18.35413361 |
| P61604 | CH10_HUMAN 10 kDa heat shock protein, mitochondrial OS | 22.73770714 | 22.60387993 | 22.33467293 | 22.72719002 |
| P61619 | S61A1_HUMAN Protein transport protein Sec61 subunit alpha isoform 1 OS | 18.47943306 | 20.18628311 | 20.03442764 | 19.72177315 |
| P61923 | COPZ1_HUMAN Coatomer subunit zeta-1 OS | 18.60433006 | 19.91628265 | 19.40555191 | 17.92468262 |
| P61970 | NTF2_HUMAN Nuclear transport factor 2 OS | 20.49891853 | 20.18531609 | 20.22811317 | 20.33330917 |
| P61978-3 | HNRPK_HUMAN Isoform 3 of Heterogeneous nuclear ribonucleoprotein K OS | 23.09923172 | 23.44037819 | 23.84373856 | 23.63234138 |
| P61981 | 1433G_HUMAN 14-3-3 protein gamma OS | 18.42302132 | 22.27104759 | 17.98696136 | 20.9958992 |
| P62081 | RS7_HUMAN 40S ribosomal protein S7 OS | 22.66921234 | 22.55978203 | 22.36847687 | 22.01127052 |
| E9PMD7 | E9PMD7_HUMAN Serine/threonine-protein phosphatase OS | 20.06539536 | 19.72168922 | 18.28643608 | 20.40395164 |
| P62191-2 | PRS4_HUMAN Isoform 2 of 26S proteasome regulatory subunit 4 OS | 19.5610466 | 20.35487747 | 18.47707939 | 17.67256355 |
| Q5JR95 | Q5JR95_HUMAN 40S ribosomal protein S8 OS | 21.89999199 | 22.16026497 | 22.126791 | 22.73442268 |
| P62244 | RS15A_HUMAN 40S ribosomal protein S15a OS | 22.8121357 | 22.11825943 | 22.24895859 | 22.83686256 |
| P62249 | RS16_HUMAN 40S ribosomal protein S16 OS | 23.02240562 | 23.15893936 | 23.09021187 | 23.08371925 |
| P62258 | 1433E_HUMAN 14-3-3 protein epsilon OS | 24.48179245 | 24.88599777 | 25.01069069 | 24.21385956 |
| A0A2R8Y811 | A0A2R8Y811_HUMAN 40S ribosomal protein S14 (Fragment) OS | 22.64425278 | 22.63916016 | 22.63143158 | 23.099617 |
| P62266 | RS23_HUMAN 40S ribosomal protein S23 OS | 19.49882126 | 20.69515038 | 20.42141914 | 20.35928154 |
| P62269 | RS18_HUMAN 40S ribosomal protein S18 OS | 22.16429138 | 22.85625839 | 22.96545029 | 22.22022247 |
| P62277 | RS13_HUMAN 40S ribosomal protein S13 OS | 22.54196167 | 23.03071213 | 22.88016891 | 23.04660225 |
| P62280 | RS11_HUMAN 40S ribosomal protein S11 OS | 21.46956062 | 22.00963211 | 22.20203781 | 21.24150085 |
| P62424 | RL7A_HUMAN 60S ribosomal protein L7a OS | 22.38474464 | 22.31705475 | 22.33772469 | 22.15927887 |
| P62495-2 | ERF1_HUMAN Isoform 2 of Eukaryotic peptide chain release factor subunit 1 OS | 19.9813385 | 19.69171333 | 18.8434906 | 19.57411385 |
| P62701 | RS4X_HUMAN 40S ribosomal protein S4, X isoform OS | 22.05480385 | 22.29698181 | 22.86287308 | 21.86619759 |
| P62750 | RL23A_HUMAN 60S ribosomal protein L23a OS | 22.6637516 | 22.13868141 | 22.56320763 | 22.01767349 |
| P62805 | H4_HUMAN Histone H4 OS | 20.7542572 | 19.5152607 | 18.8754425 | 19.59492302 |
| P62826 | RAN_HUMAN GTP-binding nuclear protein Ran OS | 23.31575584 | 23.00792313 | 23.3066082 | 24.03263092 |
| P62829 | RL23_HUMAN 60S ribosomal protein L23 OS | 21.55032349 | 22.20275497 | 19.74606705 | 21.30991364 |
| S4R456 | S4R456_HUMAN 40S ribosomal protein S15 (Fragment) OS | 18.76415825 | 18.87331963 | 18.88997269 | 18.95011711 |
| P62847-2 | RS24_HUMAN Isoform 2 of 40S ribosomal protein S24 OS | 18.87836456 | 20.34256172 | 20.33690834 | 21.84049606 |
| P62851 | RS25_HUMAN 40S ribosomal protein S25 OS | 22.20263481 | 22.06822014 | 21.82018471 | 22.31688881 |
| P62857 | RS28_HUMAN 40S ribosomal protein S28 OS | 19.75593758 | 20.7923336 | 20.42613792 | 20.1043129 |
| E5RI99 | E5RI99_HUMAN 60S ribosomal protein L30 (Fragment) OS | 19.77177048 | 20.74336815 | 20.14532089 | 20.04473495 |
| P62899 | RL31_HUMAN 60S ribosomal protein L31 OS | 21.02472305 | 21.78508568 | 21.93844032 | 21.34321213 |
| P62906 | RL10A_HUMAN 60S ribosomal protein L10a OS | 22.63526726 | 22.59279442 | 21.74996948 | 22.84684181 |
| D3YTB1 | D3YTB1_HUMAN 60S ribosomal protein L32 (Fragment) OS | 19.16825104 | 19.68024254 | 20.77531052 | 19.93445015 |
| P62913 | RL11_HUMAN 60S ribosomal protein L11 OS | 22.14099121 | 22.10126877 | 22.33279037 | 21.17907143 |
| P62917 | RL8_HUMAN 60S ribosomal protein L8 OS | 20.62220955 | 20.93660927 | 21.90142822 | 21.3046875 |
| P62937 | PPIA_HUMAN Peptidyl-prolyl cis-trans isomerase A OS | 25.39584541 | 25.18899345 | 25.30002213 | 25.47966576 |
| P63000 | RAC1_HUMAN Ras-related C3 botulinum toxin substrate 1 OS | 22.02259064 | 22.02323532 | 21.77213287 | 21.61445618 |
| P63104 | 1433Z_HUMAN 14-3-3 protein zeta/delta OS | 23.11859131 | 24.02478981 | 24.46967125 | 23.54427528 |
| P63173 | RL38_HUMAN 60S ribosomal protein L38 OS | 19.12953377 | 19.08990479 | 18.94283485 | 19.35309029 |
| I3L397 | I3L397_HUMAN Eukaryotic translation initiation factor 5A (Fragment) OS | 23.20315933 | 22.88628578 | 22.66001129 | 22.99548531 |
| P63244 | RACK1_HUMAN Receptor of activated protein C kinase 1 OS | 22.68769455 | 22.40223503 | 22.33057594 | 22.37904167 |
| P67809 | YBOX1_HUMAN Y-box-binding protein 1 OS | 22.74765587 | 22.75920486 | 22.52561569 | 22.25600433 |
| H0YNG3 | H0YNG3_HUMAN Signal peptidase complex catalytic subunit SEC11 OS | 18.22343063 | 19.51091194 | 19.41544914 | 19.53732872 |
| P67936 | TPM4_HUMAN Tropomyosin alpha-4 chain OS | 24.36084938 | 24.38007355 | 23.59434319 | 24.00547409 |
| P68032 | ACTC_HUMAN Actin, alpha cardiac muscle 1 OS | 24.74088097 | 25.19869232 | 24.25659561 | 25.02643776 |
| P68036-2 | UB2L3_HUMAN Isoform 2 of Ubiquitin-conjugating enzyme E2 L3 OS | 20.16619301 | 20.69557571 | 20.87395096 | 20.99196243 |
| A0A7I2V659 | A0A7I2V659_HUMAN Elongation factor 1-alpha 1 OS | 28.44517708 | 28.03574753 | 27.33125305 | 28.28034019 |
| P68363 | TBA1B_HUMAN Tubulin alpha-1B chain OS | 27.3512764 | 26.94826508 | 27.13359451 | 27.01393127 |
| P68371 | TBB4B_HUMAN Tubulin beta-4B chain OS | 27.17560005 | 26.76268578 | 26.85697556 | 26.43130302 |
| P78371 | TCPB_HUMAN T-complex protein 1 subunit beta OS | 21.93524361 | 22.83682251 | 22.53661156 | 22.74796295 |
| P78417 | GSTO1_HUMAN Glutathione S-transferase omega-1 OS | 22.47114944 | 21.69782448 | 21.38331985 | 22.29299927 |
| P78527 | PRKDC_HUMAN DNA-dependent protein kinase catalytic subunit OS | 21.67137337 | 21.07391739 | 19.96395111 | 19.34712219 |
| C9JXB8 | C9JXB8_HUMAN 60S ribosomal protein L24 OS | 21.07110405 | 20.63979912 | 20.12830162 | 19.10333824 |
| J3QR09 | J3QR09_HUMAN Ribosomal protein L19 OS | 18.86495018 | 17.8894558 | 18.10998154 | 19.38549232 |
| P84103-2 | SRSF3_HUMAN Isoform 2 of Serine/arginine-rich splicing factor 3 OS | 19.94692421 | 18.28826523 | 20.18180275 | 20.56010818 |
| P98179 | RBM3_HUMAN RNA-binding protein 3 OS | 20.84375763 | 20.35810089 | 20.57800865 | 21.36719704 |
| C9JFR7 | C9JFR7_HUMAN Cytochrome c (Fragment) OS | 20.96613693 | 21.44475555 | 21.244524 | 21.09841347 |
| F8VVM2 | F8VVM2_HUMAN Phosphate carrier protein, mitochondrial OS | 17.60062599 | 18.42212296 | 21.98811722 | 22.12107658 |
| A0A024R4E5 | A0A024R4E5_HUMAN High density lipoprotein binding protein (Vigilin), isoform CRA_a OS | 22.63045311 | 21.8818512 | 22.13062859 | 21.87927246 |
| Q00610-2 | CLH1_HUMAN Isoform 2 of Clathrin heavy chain 1 OS | 24.28333855 | 24.4599514 | 24.52348709 | 24.4379673 |
| A0A1W2PPS1 | A0A1W2PPS1_HUMAN Heterogeneous nuclear ribonucleoprotein U OS | 22.81362534 | 22.8461132 | 22.21252441 | 22.80616951 |
| Q01082 | SPTB2_HUMAN Spectrin beta chain, non-erythrocytic 1 OS | 20.09377861 | 20.3208046 | 20.39110565 | 19.40790939 |
| Q01105-3 | SET_HUMAN Isoform 3 of Protein SET OS | 22.15761375 | 21.89371109 | 21.63688278 | 21.83174896 |
| Q01469 | FABP5_HUMAN Fatty acid-binding protein 5 OS | 19.76960945 | 19.52793121 | 19.37285995 | 19.24328041 |
| Q01518-2 | CAP1_HUMAN Isoform 2 of Adenylyl cyclase-associated protein 1 OS | 23.38811111 | 22.81166649 | 22.88028145 | 22.88689995 |
| Q01813 | PFKAP_HUMAN ATP-dependent 6-phosphofructokinase, platelet type OS | 21.26161003 | 20.82593918 | 20.44929695 | 20.34667778 |
| Q01995 | TAGL_HUMAN Transgelin OS | 25.09583282 | 24.96128654 | 25.1590786 | 24.31264496 |
| M0R3D6 | M0R3D6_HUMAN 60S ribosomal protein L18a (Fragment) OS | 19.75468254 | 20.95659828 | 21.43043137 | 19.5927887 |
| Q02878 | RL6_HUMAN 60S ribosomal protein L6 OS | 22.6342926 | 22.74763489 | 22.59573364 | 22.70256615 |
| Q03135 | CAV1_HUMAN Caveolin-1 OS | 22.41717529 | 22.91661263 | 22.47375298 | 21.99565887 |
| Q04637-6 | IF4G1_HUMAN Isoform E of Eukaryotic translation initiation factor 4 gamma 1 OS | 18.87566185 | 21.64891052 | 21.73309898 | 21.32668686 |
| Q04917 | 1433F_HUMAN 14-3-3 protein eta OS | 22.33043861 | 21.76672554 | 21.66422844 | 21.3857975 |
| Q05682-5 | CALD1_HUMAN Isoform 5 of Caldesmon OS | 23.44695282 | 23.52324867 | 23.57957458 | 23.55145073 |
| A0A6I8PTT9 | A0A6I8PTT9_HUMAN Glutamine--fructose-6-phosphate transaminase (isomerizing) OS | 19.03189087 | 18.82517242 | 19.52383232 | 20.12578201 |
| Q06323 | PSME1_HUMAN Proteasome activator complex subunit 1 OS | 20.07313156 | 18.79440498 | 18.25384521 | 19.6867466 |
| Q06830 | PRDX1_HUMAN Peroxiredoxin-1 OS | 24.96035957 | 24.15398598 | 24.34448624 | 24.11791039 |
| Q07020 | RL18_HUMAN 60S ribosomal protein L18 OS | 23.02531433 | 23.17790031 | 22.8281498 | 21.97819901 |
| Q07065 | CKAP4_HUMAN Cytoskeleton-associated protein 4 OS | 25.25666618 | 25.28957748 | 25.32717896 | 25.23553276 |
| Q08211 | DHX9_HUMAN ATP-dependent RNA helicase A OS | 20.98454094 | 20.9061718 | 21.01065636 | 21.02614212 |
| Q09666 | AHNK_HUMAN Neuroblast differentiation-associated protein AHNAK OS | 26.62100792 | 26.41356087 | 26.47032356 | 25.94518661 |
| Q12792 | TWF1_HUMAN Twinfilin-1 OS | 19.92853546 | 19.87456703 | 19.09229469 | 19.71100426 |
| Q13162 | PRDX4_HUMAN Peroxiredoxin-4 OS | 18.68662453 | 20.78700066 | 18.63160133 | 21.92972755 |
| Q13200 | PSMD2_HUMAN 26S proteasome non-ATPase regulatory subunit 2 OS | 20.86831093 | 20.59623337 | 20.59614182 | 21.10174942 |
| A0A7I2YQU2 | A0A7I2YQU2_HUMAN Eukaryotic translation initiation factor 3 subunit I OS | 18.56655884 | 19.83174896 | 18.65626717 | 19.56917 |
| Q13404 | UB2V1_HUMAN Ubiquitin-conjugating enzyme E2 variant 1 OS | 24.62405205 | 24.62823105 | 24.45481682 | 24.98215675 |
| Q13509 | TBB3_HUMAN Tubulin beta-3 chain OS | 20.71665955 | 20.27344131 | 20.58306885 | 19.21914864 |
| Q13813-3 | SPTN1_HUMAN Isoform 3 of Spectrin alpha chain, non-erythrocytic 1 OS | 20.70902061 | 21.31744194 | 20.69905472 | 20.48424149 |
| Q13885 | TBB2A_HUMAN Tubulin beta-2A chain OS | 21.21365166 | 20.09377861 | 20.43857574 | 19.739748 |
| Q14019 | COTL1_HUMAN Coactosin-like protein OS | 21.77386284 | 21.90142822 | 22.01573563 | 21.13652229 |
| H0YA96 | H0YA96_HUMAN Heterogeneous nuclear ribonucleoprotein D0 (Fragment) OS | 22.32750893 | 21.78656197 | 21.28799438 | 22.0921669 |
| Q14152 | EIF3A_HUMAN Eukaryotic translation initiation factor 3 subunit A OS | 20.89245033 | 21.60462761 | 20.39508629 | 20.68970108 |
| Q14203-5 | DCTN1_HUMAN Isoform 5 of Dynactin subunit 1 OS | 17.61199188 | 19.21425819 | 17.94719696 | 18.45355988 |
| Q14204 | DYHC1_HUMAN Cytoplasmic dynein 1 heavy chain 1 OS | 23.90113258 | 23.65403366 | 23.85908508 | 23.16855049 |
| Q14315 | FLNC_HUMAN Filamin-C OS | 27.09194374 | 26.97421265 | 26.98185349 | 26.87244034 |
| Q14697 | GANAB_HUMAN Neutral alpha-glucosidase AB OS | 22.99762344 | 23.51941299 | 23.18717575 | 23.43529892 |
| Q14764 | MVP_HUMAN Major vault protein OS | 20.39058113 | 22.77344131 | 19.33424759 | 21.94510269 |
| Q14847 | LASP1_HUMAN LIM and SH3 domain protein 1 OS | 21.31290817 | 21.60688782 | 22.53036308 | 21.82325935 |
| Q14974 | IMB1_HUMAN Importin subunit beta-1 OS | 22.30866432 | 21.90517998 | 22.1890316 | 21.7320652 |
| Q15019 | SEPT2_HUMAN Septin-2 OS | 22.23861885 | 21.11362457 | 21.44985199 | 21.60765457 |
| Q15056-2 | IF4H_HUMAN Isoform Short of Eukaryotic translation initiation factor 4H OS | 20.77305794 | 20.79827881 | 18.79834366 | 21.32558823 |
| Q15084-3 | PDIA6_HUMAN Isoform 3 of Protein disulfide-isomerase A6 OS | 22.83953667 | 22.79237175 | 22.26224136 | 23.43338966 |
| Q15149-4 | PLEC_HUMAN Isoform 4 of Plectin OS | 24.71711922 | 24.969347 | 24.830019 | 24.10181427 |
| Q15293 | RCN1_HUMAN Reticulocalbin-1 OS | 21.11977959 | 21.70150948 | 21.04353523 | 20.97784996 |
| Q15365 | PCBP1_HUMAN Poly(rC)-binding protein 1 OS | 21.63732529 | 22.02799988 | 22.22914124 | 22.05135727 |
| Q15366-6 | PCBP2_HUMAN Isoform 6 of Poly(rC)-binding protein 2 OS | 20.50057411 | 18.698452 | 20.46483231 | 20.68294716 |
| B8ZZU8 | B8ZZU8_HUMAN Elongin-B OS | 19.77781677 | 19.90050697 | 19.78297615 | 20.01876068 |
| F5H365 | F5H365_HUMAN Protein transport protein SEC23 OS | 17.96944618 | 18.96937752 | 18.12153816 | 21.32542419 |
| Q15691 | MARE1_HUMAN Microtubule-associated protein RP/EB family member 1 OS | 17.83308029 | 20.34884071 | 19.53636169 | 20.24504662 |
| Q15758 | AAAT_HUMAN Neutral amino acid transporter B(0) OS | 20.70379257 | 20.43705368 | 20.63856316 | 20.75971222 |
| Q15907 | RB11B_HUMAN Ras-related protein Rab-11B OS | 22.26252937 | 22.26258659 | 22.11524582 | 22.28587914 |
| Q15942 | ZYX_HUMAN Zyxin OS | 22.23613739 | 22.16287804 | 22.59201813 | 22.23567009 |
| E7ES33 | E7ES33_HUMAN Septin OS | 21.20794678 | 21.85269928 | 20.24562645 | 21.16195679 |
| Q16222-3 | UAP1_HUMAN Isoform 3 of UDP-N-acetylhexosamine pyrophosphorylase OS | 19.39533806 | 19.98273277 | 20.26670837 | 20.1983242 |
| Q16527 | CSRP2_HUMAN Cysteine and glycine-rich protein 2 OS | 19.78471756 | 20.29165268 | 19.84460068 | 18.88469315 |
| Q16555-2 | DPYL2_HUMAN Isoform 2 of Dihydropyrimidinase-related protein 2 OS | 22.42393494 | 22.52125931 | 22.51662636 | 22.7605648 |
| Q16658 | FSCN1_HUMAN Fascin OS | 23.51905441 | 23.55461693 | 23.22679138 | 23.33197021 |
| A0A7I2YQ74 | A0A7I2YQ74_HUMAN UTP--glucose-1-phosphate uridylyltransferase OS | 20.23350716 | 20.25118637 | 20.38004684 | 20.29949951 |
| E9PIR7 | E9PIR7_HUMAN Thioredoxin-disulfide reductase OS | 20.91590309 | 20.79867363 | 18.58206558 | 19.95005989 |
| Q6NZI2 | CAVN1_HUMAN Caveolae-associated protein 1 OS | 22.70372963 | 23.40080261 | 23.39309692 | 23.35272408 |
| Q6UVK1 | CSPG4_HUMAN Chondroitin sulfate proteoglycan 4 OS | 19.39962006 | 19.93574715 | 20.68671227 | 19.50047684 |
| Q70UQ0-4 | IKIP_HUMAN Isoform 4 of Inhibitor of nuclear factor kappa-B kinase-interacting protein OS | 19.40643692 | 18.23005867 | 19.04209328 | 20.60503387 |
| Q7KZF4 | SND1_HUMAN Staphylococcal nuclease domain-containing protein 1 OS | 22.73789215 | 22.74517441 | 23.06307602 | 22.70459366 |
| Q86VP6 | CAND1_HUMAN Cullin-associated NEDD8-dissociated protein 1 OS | 20.53782082 | 20.93026924 | 20.72050476 | 20.99444962 |
| Q8NBS9-2 | TXND5_HUMAN Isoform 2 of Thioredoxin domain-containing protein 5 OS | 21.11273384 | 20.36879539 | 20.0941658 | 20.95496559 |
| Q8TED1 | GPX8_HUMAN Probable glutathione peroxidase 8 OS | 20.08485603 | 19.09341431 | 19.99507141 | 19.04289818 |
| Q8WUM4 | PDC6I_HUMAN Programmed cell death 6-interacting protein OS | 21.28455353 | 19.92161369 | 18.27128601 | 20.72533989 |
| Q92499-3 | DDX1_HUMAN Isoform 3 of ATP-dependent RNA helicase DDX1 OS | 18.12132645 | 20.40613365 | 18.52245331 | 18.84397888 |
| Q92598-2 | HS105_HUMAN Isoform Beta of Heat shock protein 105 kDa OS | 18.6023674 | 20.34797668 | 18.33521271 | 20.21643829 |
| Q92616 | GCN1_HUMAN eIF-2-alpha kinase activator GCN1 OS | 18.45020676 | 19.38721085 | 20.10098076 | 18.54070282 |
| A0A087WTP3 | A0A087WTP3_HUMAN Far upstream element-binding protein 2 OS | 21.17169952 | 20.74197006 | 20.79891205 | 20.47704315 |
| Q92973-2 | TNPO1_HUMAN Isoform 2 of Transportin-1 OS | 18.61182785 | 20.31959343 | 18.71065712 | 18.6649456 |
| Q969G5 | CAVN3_HUMAN Caveolae-associated protein 3 OS | 21.30969238 | 21.17846298 | 20.51595306 | 21.67878342 |
| Q969H8 | MYDGF_HUMAN Myeloid-derived growth factor OS | 21.56365013 | 21.38337326 | 21.09847641 | 21.35143089 |
| Q96AE4 | FUBP1_HUMAN Far upstream element-binding protein 1 OS | 20.86883736 | 20.4691124 | 20.22646904 | 19.21940613 |
| Q96AG4 | LRC59_HUMAN Leucine-rich repeat-containing protein 59 OS | 21.9192524 | 22.20353127 | 22.36327171 | 22.0500946 |
| Q96AY3 | FKB10_HUMAN Peptidyl-prolyl cis-trans isomerase FKBP10 OS | 20.18410492 | 19.36252594 | 20.24933624 | 19.78151894 |
| Q96HC4 | PDLI5_HUMAN PDZ and LIM domain protein 5 OS | 18.70390511 | 19.27682877 | 19.87273407 | 19.69335556 |
| Q96QK1 | VPS35_HUMAN Vacuolar protein sorting-associated protein 35 OS | 18.32221222 | 19.24896812 | 19.19289398 | 20.00990486 |
| Q96TA1-2 | NIBA2_HUMAN Isoform 2 of Protein Niban 2 OS | 19.14989853 | 20.91086197 | 20.85376549 | 20.61387253 |
| A0A087X271 | A0A087X271_HUMAN Calponin (Fragment) OS | 23.69699669 | 23.3259449 | 21.96183395 | 22.62075615 |
| A0A7I2V641 | A0A7I2V641_HUMAN 26S proteasome non-ATPase regulatory subunit 1 OS | 18.28941727 | 19.67624664 | 19.57581902 | 18.32927704 |
| Q99497 | PARK7_HUMAN Parkinson disease protein 7 OS | 22.21092033 | 22.30401993 | 22.20490456 | 21.77193069 |
| Q99536 | VAT1_HUMAN Synaptic vesicle membrane protein VAT-1 homolog OS | 22.63396072 | 22.03795052 | 19.75020599 | 21.72317505 |
| F5GY37 | F5GY37_HUMAN Prohibitin OS | 21.92788506 | 21.71962738 | 20.81550217 | 21.71895981 |
| Q99715-4 | COCA1_HUMAN Isoform 4 of Collagen alpha-1(XII) chain OS | 19.68501854 | 19.96253967 | 19.78284836 | 18.75119781 |
| Q99832 | TCPH_HUMAN T-complex protein 1 subunit eta OS | 21.34884071 | 20.57800865 | 21.46856689 | 21.84352875 |
| Q9BRA2 | TXD17_HUMAN Thioredoxin domain-containing protein 17 OS | 19.84862518 | 19.68291283 | 20.00675964 | 20.44717979 |
| Q9BSJ8 | ESYT1_HUMAN Extended synaptotagmin-1 OS | 20.17803764 | 20.97673225 | 20.32223701 | 20.76773453 |
| Q9BUF5 | TBB6_HUMAN Tubulin beta-6 chain OS | 23.14765167 | 22.41423607 | 22.6908741 | 22.16459846 |
| Q9BVK6 | TMED9_HUMAN Transmembrane emp24 domain-containing protein 9 OS | 19.98036194 | 20.52008629 | 19.68292999 | 20.33712578 |
| Q9H0U4 | RAB1B_HUMAN Ras-related protein Rab-1B OS | 22.75906181 | 22.75963211 | 22.9252243 | 22.09326363 |
| Q5T123 | Q5T123_HUMAN SH3 domain-binding glutamic acid-rich-like protein 3 OS | 21.15189934 | 18.7892971 | 18.71455956 | 20.36292458 |
| Q9H3N1 | TMX1_HUMAN Thioredoxin-related transmembrane protein 1 OS | 18.19048309 | 20.78668213 | 19.18562889 | 17.42985535 |
| Q9H4M9 | EHD1_HUMAN EH domain-containing protein 1 OS | 18.41928482 | 19.01069069 | 20.92672729 | 21.23502731 |
| Q9HB71 | CYBP_HUMAN Calcyclin-binding protein OS | 18.70917511 | 20.5643959 | 18.91725349 | 18.86560249 |
| A0A087X163 | A0A087X163_HUMAN Ras-related protein Rab-18 OS | 18.61141777 | 19.37511063 | 18.93945313 | 18.47066879 |
| Q9NQC3-2 | RTN4_HUMAN Isoform B of Reticulon-4 OS | 23.34272385 | 22.8412838 | 22.71078682 | 23.19751549 |
| Q9NR12 | PDLI7_HUMAN PDZ and LIM domain protein 7 OS | 18.79019356 | 19.67226219 | 19.06404877 | 19.15857887 |
| Q9NRV9 | HEBP1_HUMAN Heme-binding protein 1 OS | 18.67448997 | 18.21212578 | 19.93890762 | 18.86046219 |
| D6RGI3 | D6RGI3_HUMAN Septin OS | 22.12164688 | 21.77996635 | 21.67998695 | 21.34651566 |
| Q9NZM1-6 | MYOF_HUMAN Isoform 6 of Myoferlin OS | 23.84957314 | 23.01350403 | 23.11109543 | 22.56048203 |
| Q9NZN4 | EHD2_HUMAN EH domain-containing protein 2 OS | 22.09329414 | 21.28421402 | 21.56644058 | 21.16373825 |
| Q9P0L0 | VAPA_HUMAN Vesicle-associated membrane protein-associated protein A OS | 19.88254547 | 19.76885033 | 19.23502541 | 18.76872063 |
| Q9P2E9 | RRBP1_HUMAN Ribosome-binding protein 1 OS | 23.50178909 | 23.40911674 | 23.15705872 | 23.19231606 |
| F8VQE1 | F8VQE1_HUMAN LIM domain and actin-binding protein 1 OS | 21.16974449 | 20.71749496 | 18.20223618 | 20.70480537 |
| Q9UHD8-7 | SEPT9_HUMAN Isoform 7 of Septin-9 OS | 22.38955688 | 22.09026146 | 22.11565781 | 22.17273712 |
| Q9ULV4 | COR1C_HUMAN Coronin-1C OS | 20.39267731 | 20.36591721 | 21.1221199 | 20.94320679 |
| Q9UQ80-2 | PA2G4_HUMAN Isoform 2 of Proliferation-associated protein 2G4 OS | 21.8764267 | 21.51931763 | 21.58664513 | 21.97620773 |
| Q9Y230 | RUVB2_HUMAN RuvB-like 2 OS | 18.30500984 | 18.33820915 | 20.23128128 | 19.30720139 |
| Q9Y265 | RUVB1_HUMAN RuvB-like 1 OS | 18.55114746 | 20.03335381 | 20.45100975 | 20.28557014 |
| Q9Y266 | NUDC_HUMAN Nuclear migration protein nudC OS | 19.0090847 | 19.68888283 | 17.75411606 | 18.9045372 |
| Q9Y3U8 | RL36_HUMAN 60S ribosomal protein L36 OS | 20.48197746 | 21.4355793 | 20.8459034 | 20.64570427 |
| Q9Y490 | TLN1_HUMAN Talin-1 OS | 24.84388351 | 24.7702179 | 24.88920403 | 24.74180603 |
| A0A087X054 | A0A087X054_HUMAN Hypoxia up-regulated protein 1 OS | 20.05642509 | 20.02485847 | 21.03106499 | 19.64225197 |
| Q9Y617-2 | SERC_HUMAN Isoform 2 of Phosphoserine aminotransferase OS | 21.74176407 | 21.5323143 | 21.55982971 | 21.35681343 |
| Q9Y678 | COPG1_HUMAN Coatomer subunit gamma-1 OS | 21.22640991 | 21.85551643 | 21.95521355 | 21.34705734 |
| Q9Y696 | CLIC4_HUMAN Chloride intracellular channel protein 4 OS | 22.36956787 | 22.27673721 | 21.01277161 | 21.85365295 |

| **Accession** | **Protein** | **LFQ intensity eGFP 48hrs** | **LFQ intensity Envelope 48hrs** | **LFQ intensity Membrane 48hrs** | **LFQ intensity Nucleocapsid 48hrs** |
| --- | --- | --- | --- | --- | --- |
| H3BTL1 | H3BTL1_HUMAN Microtubule-associated protein 1 light chain 3 beta, isoform CRA_f OS | 20.61602783 | 19.31570053 | 20.51749229 | 20.4514122 |
| Q99613-2 | EIF3C_HUMAN Isoform 2 of Eukaryotic translation initiation factor 3 subunit C OS | 20.29052734 | 20.32695961 | 20.18664551 | 20.28263092 |
| F8VZJ2 | F8VZJ2_HUMAN Nascent polypeptide-associated complex subunit alpha OS | 21.19904518 | 18.63215828 | 21.84230042 | 22.28022575 |
| O00148 | DX39A_HUMAN ATP-dependent RNA helicase DDX39A OS | 20.31118965 | 20.01835251 | 20.09571075 | 18.59388924 |
| O00159-2 | MYO1C_HUMAN Isoform 2 of Unconventional myosin-Ic OS | 22.21106911 | 21.78320694 | 21.85733986 | 22.21380043 |
| O00231 | PSD11_HUMAN 26S proteasome non-ATPase regulatory subunit 11 OS | 21.21406746 | 20.9754734 | 21.56899261 | 21.15480614 |
| O00232-2 | PSD12_HUMAN Isoform 2 of 26S proteasome non-ATPase regulatory subunit 12 OS | 20.46513176 | 19.75865555 | 19.89496803 | 18.77387047 |
| O00299 | CLIC1_HUMAN Chloride intracellular channel protein 1 OS | 24.23066711 | 24.3347683 | 24.61071014 | 23.91988945 |
| O00303 | EIF3F_HUMAN Eukaryotic translation initiation factor 3 subunit F OS | 19.38295746 | 18.56738091 | 20.39571381 | 18.66461372 |
| O00410 | IPO5_HUMAN Importin-5 OS | 21.32849503 | 21.32350159 | 21.36890221 | 21.21204948 |
| A0A2R8Y5G6 | A0A2R8Y5G6_HUMAN RNA helicase OS | 21.15684319 | 21.00915337 | 20.47040558 | 20.73859406 |
| O14818 | PSA7_HUMAN Proteasome subunit alpha type-7 OS | 22.06785774 | 22.11581802 | 22.22925949 | 22.20669556 |
| P19105 | ML12A_HUMAN Myosin regulatory light chain 12A OS | 23.62399483 | 23.00559425 | 23.69508553 | 23.48000526 |
| O15143 | ARC1B_HUMAN Actin-related protein 2/3 complex subunit 1B OS | 21.59700775 | 20.73809814 | 21.18755341 | 21.15455818 |
| O15144 | ARPC2_HUMAN Actin-related protein 2/3 complex subunit 2 OS | 20.68628502 | 18.87779427 | 21.54858398 | 21.10232735 |
| A0A286YF22 | A0A286YF22_HUMAN D-3-phosphoglycerate dehydrogenase OS | 22.08229065 | 21.99603844 | 21.67499733 | 21.21092033 |
| O43242 | PSMD3_HUMAN 26S proteasome non-ATPase regulatory subunit 3 OS | 20.24411774 | 20.6088295 | 20.23899651 | 20.32794762 |
| O43707 | ACTN4_HUMAN Alpha-actinin-4 OS | 23.97561264 | 23.64603424 | 23.7552166 | 24.17119598 |
| O43795-2 | MYO1B_HUMAN Isoform 2 of Unconventional myosin-Ib OS | 20.40114212 | 19.9607029 | 19.70079803 | 20.30529976 |
| O43852 | CALU_HUMAN Calumenin OS | 23.17360687 | 23.74375916 | 23.61862946 | 22.98340988 |
| O60506-4 | HNRPQ_HUMAN Isoform 4 of Heterogeneous nuclear ribonucleoprotein Q OS | 23.01831818 | 21.88136482 | 21.81979561 | 22.45603371 |
| O60664-4 | PLIN3_HUMAN Isoform 4 of Perilipin-3 OS | 22.88912964 | 22.62209702 | 22.99765778 | 22.50491905 |
| O60701 | UGDH_HUMAN UDP-glucose 6-dehydrogenase OS | 23.14577103 | 23.81723976 | 23.6198616 | 23.17949677 |
| O60763 | USO1_HUMAN General vesicular transport factor p115 OS | 20.40062141 | 19.94377899 | 19.95503616 | 20.12338448 |
| O75083 | WDR1_HUMAN WD repeat-containing protein 1 OS | 23.52169228 | 23.40483665 | 23.53575897 | 23.6003685 |
| E5RIW3 | E5RIW3_HUMAN Tubulin-specific chaperone A OS | 20.54745483 | 20.4355278 | 20.63129807 | 18.83438873 |
| O75369-2 | FLNB_HUMAN Isoform 2 of Filamin-B OS | 23.62878609 | 23.7702179 | 23.60614204 | 23.62667084 |
| B4DJV2 | B4DJV2_HUMAN Citrate synthase OS | 21.43903351 | 20.50106049 | 21.43786621 | 21.09029388 |
| O75396 | SC22B_HUMAN Vesicle-trafficking protein SEC22b OS | 22.06887627 | 21.81323242 | 21.65285301 | 22.25245667 |
| O76003 | GLRX3_HUMAN Glutaredoxin-3 OS | 18.77362633 | 19.87885284 | 18.01993942 | 18.97174072 |
| O94979-3 | SC31A_HUMAN Isoform 3 of Protein transport protein Sec31A OS | 21.21501541 | 20.64640808 | 21.30123138 | 21.05867386 |
| O95373 | IPO7_HUMAN Importin-7 OS | 21.0726738 | 20.87882233 | 20.47050476 | 20.38479614 |
| O95782-2 | AP2A1_HUMAN Isoform B of AP-2 complex subunit alpha-1 OS | 19.65579987 | 19.41734505 | 19.11441422 | 18.37331772 |
| O95816 | BAG2_HUMAN BAG family molecular chaperone regulator 2 OS | 20.03778267 | 19.60074043 | 19.6768322 | 19.50629997 |
| P00338 | LDHA_HUMAN L-lactate dehydrogenase A chain OS | 25.59931946 | 24.21178246 | 25.64121819 | 25.35194969 |
| P00367 | DHE3_HUMAN Glutamate dehydrogenase 1, mitochondrial OS | 20.1382122 | 21.23257065 | 20.90771294 | 21.21199036 |
| P00387-2 | NB5R3_HUMAN Isoform 2 of NADH-cytochrome b5 reductase 3 OS | 22.25161934 | 20.54113388 | 22.09635544 | 22.2870369 |
| P00403 | COX2_HUMAN Cytochrome c oxidase subunit 2 OS | 20.18519402 | 20.31030273 | 20.06276321 | 17.92194557 |
| P00505-2 | AATM_HUMAN Isoform 2 of Aspartate aminotransferase, mitochondrial OS | 20.38711357 | 19.84133339 | 19.64602089 | 20.5334549 |
| P00558 | PGK1_HUMAN Phosphoglycerate kinase 1 OS | 23.70038795 | 24.01077652 | 23.98085022 | 23.66231728 |
| P00568 | KAD1_HUMAN Adenylate kinase isoenzyme 1 OS | 20.12691689 | 19.76556778 | 19.90935326 | 20.07156181 |
| P02452 | CO1A1_HUMAN Collagen alpha-1(I) chain OS | 25.26054955 | 25.17157745 | 25.11787033 | 25.20407677 |
| P02545 | LMNA_HUMAN Prelamin-A/C OS | 25.40291786 | 25.47512627 | 25.71319008 | 25.26456261 |
| P02751-5 | FINC_HUMAN Isoform 5 of Fibronectin OS | 21.25902557 | 21.49921227 | 20.68551445 | 21.11426163 |
| P02786 | TFR1_HUMAN Transferrin receptor protein 1 OS | 21.27685165 | 20.27161789 | 20.21714783 | 21.07365608 |
| P04075 | ALDOA_HUMAN Fructose-bisphosphate aldolase A OS | 25.17352295 | 25.48793602 | 24.71093369 | 25.2035923 |
| P04080 | CYTB_HUMAN Cystatin-B OS | 21.09596825 | 20.51152039 | 20.36099625 | 21.31986809 |
| P04083 | ANXA1_HUMAN Annexin A1 OS | 25.85984421 | 25.46245003 | 25.31451225 | 25.27191734 |
| P04181 | OAT_HUMAN Ornithine aminotransferase, mitochondrial OS | 19.77516747 | 20.05576515 | 20.26899338 | 18.02760124 |
| P04350 | TBB4A_HUMAN Tubulin beta-4A chain OS | 21.51821327 | 20.98974609 | 21.78831482 | 20.72966003 |
| P04406 | G3P_HUMAN Glyceraldehyde-3-phosphate dehydrogenase OS | 26.29617691 | 25.36649704 | 25.45741844 | 25.5820179 |
| K7EM73 | K7EM73_HUMAN Calcium-activated neutral proteinase small subunit (Fragment) OS | 21.22205162 | 21.29473877 | 21.43822098 | 21.59363747 |
| A0A6Q8PFK8 | A0A6Q8PFK8_HUMAN Heat shock protein beta-1 OS | 24.37245369 | 24.6660614 | 24.20254517 | 24.95628738 |
| P04843 | RPN1_HUMAN Dolichyl-diphosphooligosaccharide--protein glycosyltransferase subunit 1 OS | 23.33046532 | 23.2104454 | 22.76013947 | 23.20088577 |
| P04844-2 | RPN2_HUMAN Isoform 2 of Dolichyl-diphosphooligosaccharide--protein glycosyltransferase subunit 2 OS | 22.18365097 | 21.64403152 | 21.6032238 | 21.49658012 |
| P05023-3 | AT1A1_HUMAN Isoform 3 of Sodium/potassium-transporting ATPase subunit alpha-1 OS | 21.11057091 | 21.35525513 | 20.99113083 | 21.16263199 |
| P05141 | ADT2_HUMAN ADP/ATP translocase 2 OS | 21.48365021 | 22.39949989 | 22.11069679 | 22.44609451 |
| P05387 | RLA2_HUMAN 60S acidic ribosomal protein P2 OS | 20.08770561 | 20.49589729 | 19.80579185 | 20.27514648 |
| P05388 | RLA0_HUMAN 60S acidic ribosomal protein P0 OS | 22.57147217 | 22.75007057 | 22.37530708 | 22.33992767 |
| P05455 | LA_HUMAN Lupus La protein OS | 20.00429153 | 21.74291611 | 20.257761 | 20.50193405 |
| H7C4K3 | H7C4K3_HUMAN Integrin beta-1 OS | 22.8581562 | 23.02396202 | 23.01133919 | 22.98018837 |
| P06576 | ATPB_HUMAN ATP synthase subunit beta, mitochondrial OS | 24.12344742 | 23.96410942 | 23.83740044 | 24.01996422 |
| R4GN98 | R4GN98_HUMAN Protein S100 (Fragment) OS | 20.72208977 | 21.599823 | 20.35906792 | 20.35767174 |
| P06733 | ENOA_HUMAN Alpha-enolase OS | 26.74111176 | 26.54545403 | 26.79426765 | 26.75684547 |
| P06744 | G6PI_HUMAN Glucose-6-phosphate isomerase OS | 22.42864418 | 22.34499931 | 22.41763878 | 22.10261536 |
| P06748-3 | NPM_HUMAN Isoform 3 of Nucleophosmin OS | 22.79336548 | 22.7108078 | 22.87464523 | 23.0128746 |
| P06753-2 | TPM3_HUMAN Isoform 2 of Tropomyosin alpha-3 chain OS | 24.54250526 | 25.21337891 | 24.96886253 | 24.77700806 |
| P07195 | LDHB_HUMAN L-lactate dehydrogenase B chain OS | 23.85880089 | 23.40002251 | 23.93409157 | 23.63289642 |
| P07237 | PDIA1_HUMAN Protein disulfide-isomerase OS | 24.70054626 | 24.93111801 | 24.70677757 | 24.89254379 |
| P07355 | ANXA2_HUMAN Annexin A2 OS | 26.59733009 | 26.1086483 | 26.18290901 | 26.60865402 |
| Q5JP53 | Q5JP53_HUMAN Tubulin beta chain OS | 25.12904167 | 25.62220955 | 25.58840752 | 25.11521339 |
| P07737 | PROF1_HUMAN Profilin-1 OS | 25.58626175 | 25.19636726 | 25.59391594 | 25.46323013 |
| P07741-2 | APT_HUMAN Isoform 2 of Adenine phosphoribosyltransferase OS | 19.62508392 | 19.3804493 | 19.40645027 | 18.29244232 |
| P07814 | SYEP_HUMAN Bifunctional glutamate/proline--tRNA ligase OS | 21.41245461 | 21.20251656 | 21.11711884 | 20.71498299 |
| A0A7I2V668 | A0A7I2V668_HUMAN Cathepsin B OS | 21.01352119 | 21.11222649 | 18.72297287 | 21.22864342 |
| P07900 | HS90A_HUMAN Heat shock protein HSP 90-alpha OS | 25.17866135 | 25.23066711 | 25.19490433 | 25.04485321 |
| P07954-2 | FUMH_HUMAN Isoform Cytoplasmic of Fumarate hydratase, mitochondrial OS | 20.41069603 | 20.77940559 | 20.80970573 | 18.50860214 |
| A0A087WTA8 | A0A087WTA8_HUMAN Collagen alpha-2(I) chain OS | 24.04009247 | 24.18971062 | 23.89050484 | 23.835186 |
| P08133-2 | ANXA6_HUMAN Isoform 2 of Annexin A6 OS | 23.14861298 | 22.47397614 | 22.0764637 | 23.00011826 |
| P08195-2 | 4F2_HUMAN Isoform 2 of 4F2 cell-surface antigen heavy chain OS | 21.80278206 | 21.16545677 | 18.55701637 | 21.57846832 |
| P08238 | HS90B_HUMAN Heat shock protein HSP 90-beta OS | 26.72103691 | 27.03601074 | 26.61259651 | 26.97278976 |
| P08670 | VIME_HUMAN Vimentin OS | 27.92643166 | 27.78144264 | 27.94642639 | 27.59967422 |
| P08708 | RS17_HUMAN 40S ribosomal protein S17 OS | 19.34197617 | 20.06999016 | 20.12401581 | 20.2693367 |
| P08758 | ANXA5_HUMAN Annexin A5 OS | 25.90728951 | 24.90370941 | 26.21661949 | 25.90274429 |
| C9J9K3 | C9J9K3_HUMAN 40S ribosomal protein SA (Fragment) OS | 22.93883514 | 22.66955757 | 22.58297539 | 23.36932945 |
| P09211 | GSTP1_HUMAN Glutathione S-transferase P OS | 23.5114727 | 24.15506744 | 24.5366478 | 24.07879448 |
| P09382 | LEG1_HUMAN Galectin-1 OS | 24.75139046 | 25.20910645 | 25.52966499 | 25.35910797 |
| P09493-3 | TPM1_HUMAN Isoform 3 of Tropomyosin alpha-1 chain OS | 23.28686714 | 22.82801437 | 23.23216057 | 23.02414894 |
| P09651-3 | ROA1_HUMAN Isoform 2 of Heterogeneous nuclear ribonucleoprotein A1 OS | 22.3688221 | 23.61031723 | 22.52075768 | 22.28940201 |
| P09936 | UCHL1_HUMAN Ubiquitin carboxyl-terminal hydrolase isozyme L1 OS | 22.0267849 | 21.69947815 | 21.65302849 | 22.27463531 |
| J3QS39 | J3QS39_HUMAN Polyubiquitin-B (Fragment) OS | 25.43083954 | 25.14230728 | 25.24793243 | 25.28866386 |
| P0DMV8-2 | HS71A_HUMAN Isoform 2 of Heat shock 70 kDa protein 1A OS | 23.30480003 | 22.37289238 | 22.56043625 | 23.17430878 |
| P0DP25 | CALM3_HUMAN Calmodulin-3 OS | 22.3113842 | 21.35563087 | 22.31705475 | 21.82609558 |
| P10599 | THIO_HUMAN Thioredoxin OS | 23.29516029 | 22.97388077 | 22.84155273 | 22.73783112 |
| A0A7I2V599 | A0A7I2V599_HUMAN 60 kDa heat shock protein, mitochondrial OS | 23.77655602 | 24.07373619 | 24.05191994 | 24.16629982 |
| P11021 | BIP_HUMAN Endoplasmic reticulum chaperone BiP OS | 26.22101593 | 26.02892685 | 25.72832298 | 26.22131157 |
| P11142 | HSP7C_HUMAN Heat shock cognate 71 kDa protein OS | 26.54633713 | 26.52459335 | 26.42298889 | 26.63339424 |
| P11279 | LAMP1_HUMAN Lysosome-associated membrane glycoprotein 1 OS | 21.02884293 | 21.24504662 | 18.07195663 | 18.31446075 |
| P11413 | G6PD_HUMAN Glucose-6-phosphate 1-dehydrogenase OS | 23.51159286 | 23.81303787 | 23.84249306 | 23.78954887 |
| V9GYY3 | V9GYY3_HUMAN C-1-tetrahydrofolate synthase, cytoplasmic OS | 20.76902771 | 20.7466526 | 20.46463394 | 20.99133873 |
| P11940-2 | PABP1_HUMAN Isoform 2 of Polyadenylate-binding protein 1 OS | 22.49689674 | 22.47951126 | 21.75678444 | 22.63325119 |
| P12004 | PCNA_HUMAN Proliferating cell nuclear antigen OS | 19.69995117 | 19.57966614 | 19.51034355 | 19.98189545 |
| A0A087X0S5 | A0A087X0S5_HUMAN Collagen alpha-1(VI) chain OS | 20.94756508 | 20.67796707 | 20.38131523 | 20.73900604 |
| P12110-3 | CO6A2_HUMAN Isoform 2C2A of Collagen alpha-2(VI) chain OS | 20.41379738 | 19.88619614 | 19.87937737 | 19.74571609 |
| P12111-2 | CO6A3_HUMAN Isoform 2 of Collagen alpha-3(VI) chain OS | 21.92321205 | 22.11158943 | 22.16177177 | 21.7786026 |
| P12236 | ADT3_HUMAN ADP/ATP translocase 3 OS | 20.45402527 | 20.4596405 | 21.09699821 | 21.72953606 |
| E7ETK5 | E7ETK5_HUMAN Inosine-5-monophosphate dehydrogenase 2 OS | 19.78273582 | 20.56504631 | 20.41751099 | 20.21204948 |
| P12814 | ACTN1_HUMAN Alpha-actinin-1 OS | 23.76698685 | 23.12697983 | 23.49191666 | 23.29516029 |
| P12956 | XRCC6_HUMAN X-ray repair cross-complementing protein 6 OS | 22.56695175 | 22.32465553 | 21.75319672 | 22.34519005 |
| P13010 | XRCC5_HUMAN X-ray repair cross-complementing protein 5 OS | 21.67856979 | 21.08582687 | 20.87199783 | 21.24736595 |
| P13489 | RINI_HUMAN Ribonuclease inhibitor OS | 21.70446777 | 18.20135307 | 21.84804535 | 21.52387047 |
| P13639 | EF2_HUMAN Elongation factor 2 OS | 25.30218697 | 25.73259735 | 26.16109848 | 25.58972359 |
| P13667 | PDIA4_HUMAN Protein disulfide-isomerase A4 OS | 22.57482529 | 22.61261368 | 22.5365181 | 22.94567299 |
| P13693 | TCTP_HUMAN Translationally-controlled tumor protein OS | 22.15591812 | 21.87428856 | 21.91148376 | 22.06292725 |
| P13797-3 | PLST_HUMAN Isoform 3 of Plastin-3 OS | 21.77096367 | 21.26080704 | 20.74098396 | 21.45347404 |
| P14314-2 | GLU2B_HUMAN Isoform 2 of Glucosidase 2 subunit beta OS | 21.38047028 | 21.60086823 | 22.08255005 | 21.5177803 |
| P14618 | KPYM_HUMAN Pyruvate kinase PKM OS | 27.30426025 | 27.35951042 | 26.99269676 | 27.45615005 |
| P14625 | ENPL_HUMAN Endoplasmin OS | 25.62522316 | 25.66901207 | 25.58588982 | 25.55218506 |
| P14868-2 | SYDC_HUMAN Isoform 2 of Aspartate--tRNA ligase, cytoplasmic OS | 20.97568321 | 21.10290337 | 20.97098732 | 21.48084259 |
| P15121 | ALDR_HUMAN Aldo-keto reductase family 1 member B1 OS | 20.20012283 | 19.66695213 | 20.00072098 | 20.58710289 |
| P15170-2 | ERF3A_HUMAN Isoform 2 of Eukaryotic peptide chain release factor GTP-binding subunit ERF3A OS | 19.17871857 | 18.8816967 | 18.65241432 | 18.97840881 |
| E7EQR4 | E7EQR4_HUMAN Ezrin OS | 21.62408447 | 21.44637299 | 21.66353416 | 21.60648155 |
| P15531 | NDKA_HUMAN Nucleoside diphosphate kinase A OS | 20.1347065 | 20.28353691 | 19.48254776 | 20.32454491 |
| B4DLR8 | B4DLR8_HUMAN NAD(P)H dehydrogenase [quinone] 1 OS | 22.44753265 | 21.86578369 | 22.30376816 | 22.73442268 |
| P15880 | RS2_HUMAN 40S ribosomal protein S2 OS | 21.24411774 | 18.87893677 | 21.9693718 | 21.77503014 |
| H0YD13 | H0YD13_HUMAN CD44 antigen OS | 22.43895721 | 23.2060833 | 23.46063995 | 22.74071503 |
| P16152 | CBR1_HUMAN Carbonyl reductase [NADPH] 1 OS | 20.7833271 | 20.15400314 | 20.06276321 | 21.21957207 |
| P16401 | H15_HUMAN Histone H1.5 OS | 18.7462368 | 19.19828224 | 20.49921227 | 18.50682259 |
| P16403 | H12_HUMAN Histone H1.2 OS | 22.38640213 | 18.74824715 | 22.90209007 | 22.68478775 |
| A2A2D0 | A2A2D0_HUMAN Stathmin (Fragment) OS | 22.34226227 | 22.94529915 | 22.71406174 | 22.72735596 |
| P17066 | HSP76_HUMAN Heat shock 70 kDa protein 6 OS | 24.61397362 | 18.26551056 | 24.45437813 | 23.79897118 |
| P17301 | ITA2_HUMAN Integrin alpha-2 OS | 20.31406975 | 20.44505882 | 21.03624153 | 19.52389908 |
| P17844-2 | DDX5_HUMAN Isoform 2 of Probable ATP-dependent RNA helicase DDX5 OS | 21.87075806 | 21.69782448 | 21.48718834 | 22.00747681 |
| P17931 | LEG3_HUMAN Galectin-3 OS | 21.06651306 | 20.39863968 | 20.80388451 | 21.38120842 |
| P17987 | TCPA_HUMAN T-complex protein 1 subunit alpha OS | 22.82805443 | 22.23224831 | 22.20341301 | 21.87271309 |
| P18077 | RL35A_HUMAN 60S ribosomal protein L35a OS | 21.2151947 | 20.62952042 | 20.89533806 | 18.39235115 |
| P18085 | ARF4_HUMAN ADP-ribosylation factor 4 OS | 23.32635689 | 23.1848774 | 23.47691917 | 23.37491035 |
| P18124 | RL7_HUMAN 60S ribosomal protein L7 OS | 22.77858353 | 22.86116982 | 22.92049026 | 21.71276093 |
| P18206-2 | VINC_HUMAN Isoform 1 of Vinculin OS | 25.69399643 | 25.5762043 | 25.60995102 | 25.6798687 |
| A0A087WXM6 | A0A087WXM6_HUMAN 60S ribosomal protein L17 (Fragment) OS | 21.83194351 | 22.09371376 | 22.00915337 | 22.17660713 |
| P18669 | PGAM1_HUMAN Phosphoglycerate mutase 1 OS | 22.57996559 | 22.89827538 | 22.50629997 | 22.94472694 |
| A0A7I2V5M5 | A0A7I2V5M5_HUMAN Nucleolin OS | 24.28376198 | 24.15305901 | 23.8727684 | 24.26492119 |
| P20340-2 | RAB6A_HUMAN Isoform 2 of Ras-related protein Rab-6A OS | 20.35035133 | 20.64728546 | 19.22706413 | 20.53772736 |
| P20618 | PSB1_HUMAN Proteasome subunit beta type-1 OS | 21.58879662 | 21.0796566 | 21.55314064 | 21.32756424 |
| E9PP21 | E9PP21_HUMAN Cysteine and glycine-rich protein 1 OS | 22.84471703 | 22.3922596 | 22.31274033 | 23.36546326 |
| P21333-2 | FLNA_HUMAN Isoform 2 of Filamin-A OS | 27.62086868 | 27.82468796 | 27.72916603 | 27.72227478 |
| P21796 | VDAC1_HUMAN Voltage-dependent anion-selective channel protein 1 OS | 20.6687355 | 18.72044563 | 21.33848572 | 21.56583595 |
| P21980 | TGM2_HUMAN Protein-glutamine gamma-glutamyltransferase 2 OS | 21.30513382 | 21.54787827 | 20.48079491 | 21.22193336 |
| P22234 | PUR6_HUMAN Multifunctional protein ADE2 OS | 19.85329437 | 20.30039215 | 20.32926178 | 19.82730484 |
| P22307-6 | SCP2_HUMAN Isoform 6 of Sterol carrier protein 2 OS | 20.27605629 | 20.05999374 | 19.90847397 | 19.79895973 |
| P22314-2 | UBA1_HUMAN Isoform 2 of Ubiquitin-like modifier-activating enzyme 1 OS | 23.89458084 | 23.74201202 | 23.80262375 | 23.73923302 |
| P22392-2 | NDKB_HUMAN Isoform 3 of Nucleoside diphosphate kinase B OS | 24.87084198 | 24.58703423 | 25.027071 | 24.5736351 |
| A0A7I2V4I6 | A0A7I2V4I6_HUMAN Heterogeneous nuclear ribonucleoproteins A2/B1 OS | 22.01185036 | 21.45728683 | 22.39076424 | 21.95822716 |
| A0A2R8YDM0 | A0A2R8YDM0_HUMAN Prostaglandin-endoperoxide synthase (Fragment) OS | 20.32586288 | 20.18046761 | 18.31494141 | 20.08160782 |
| P23246 | SFPQ_HUMAN Splicing factor, proline- and glutamine-rich OS | 21.93700409 | 22.39204979 | 22.44929695 | 22.170784 |
| P23284 | PPIB_HUMAN Peptidyl-prolyl cis-trans isomerase B OS | 24.49307823 | 24.43083954 | 24.33203888 | 24.61801338 |
| P23381-2 | SYWC_HUMAN Isoform 2 of Tryptophan--tRNA ligase, cytoplasmic OS | 19.58361816 | 19.61547089 | 19.94206238 | 19.09093857 |
| P23396 | RS3_HUMAN 40S ribosomal protein S3 OS | 24.05589676 | 23.84661102 | 24.10717392 | 23.0134201 |
| P23526 | SAHH_HUMAN Adenosylhomocysteinase OS | 21.42639351 | 21.24341965 | 21.40374374 | 21.49896812 |
| P23528 | COF1_HUMAN Cofilin-1 OS | 25.63425446 | 25.41902351 | 25.51023483 | 25.02356529 |
| P23634-7 | AT2B4_HUMAN Isoform ZB of Plasma membrane calcium-transporting ATPase 4 OS | 19.09305573 | 18.99697495 | 19.97070694 | 19.05999374 |
| P24534 | EF1B_HUMAN Elongation factor 1-beta OS | 22.53945541 | 22.28398895 | 23.46699905 | 18.71809959 |
| Q5QNZ2 | Q5QNZ2_HUMAN ATP synthase F(0) complex subunit B1, mitochondrial OS | 19.74175644 | 19.88176727 | 19.82468224 | 20.20717239 |
| P25398 | RS12_HUMAN 40S ribosomal protein S12 OS | 21.24010468 | 21.19111252 | 20.75702858 | 21.18960571 |
| P25705 | ATPA_HUMAN ATP synthase subunit alpha, mitochondrial OS | 24.06899071 | 24.1180687 | 24.17554283 | 23.322649 |
| F5GX11 | F5GX11_HUMAN Proteasome subunit alpha type-1 OS | 20.89326668 | 21.15776825 | 21.75991631 | 20.15721321 |
| A0A7I2V2H3 | A0A7I2V2H3_HUMAN UPF0415 protein C7orf25 OS | 18.28251839 | 20.25395775 | 20.9078598 | 21.27560234 |
| P25788-2 | PSA3_HUMAN Isoform 2 of Proteasome subunit alpha type-3 OS | 21.40504456 | 21.23473549 | 20.93437958 | 18.14051628 |
| H0YMZ1 | H0YMZ1_HUMAN Proteasome subunit alpha type (Fragment) OS | 20.68918991 | 20.87342644 | 20.73355484 | 20.08511543 |
| P26038 | MOES_HUMAN Moesin OS | 24.19355011 | 24.49405479 | 24.2624855 | 24.43338966 |
| P26373 | RL13_HUMAN 60S ribosomal protein L13 OS | 22.53971481 | 22.312603 | 22.59839249 | 22.6654644 |
| A6NLN1 | A6NLN1_HUMAN Polypyrimidine tract-binding protein 1 OS | 21.72538185 | 22.15412521 | 21.51383591 | 22.02242279 |
| P26639 | SYTC_HUMAN Threonine--tRNA ligase 1, cytoplasmic OS | 20.62551308 | 20.51951027 | 20.79177666 | 21.08634567 |
| P26640 | SYVC_HUMAN Valine--tRNA ligase OS | 20.20836258 | 20.69752693 | 20.3126297 | 20.14307976 |
| P26641 | EF1G_HUMAN Elongation factor 1-gamma OS | 23.89993668 | 23.68474579 | 24.16851997 | 23.83026123 |
| P27348 | 1433T_HUMAN 14-3-3 protein theta OS | 22.66890907 | 22.60295296 | 22.68429565 | 22.8053627 |
| F8W7C6 | F8W7C6_HUMAN 60S ribosomal protein L10 OS | 20.09777069 | 20.208601 | 20.27491951 | 20.58737755 |
| P27797 | CALR_HUMAN Calreticulin OS | 24.5749054 | 23.92524338 | 23.95287132 | 24.04518509 |
| P27816-6 | MAP4_HUMAN Isoform 6 of Microtubule-associated protein 4 OS | 22.35584641 | 22.31890297 | 21.99334526 | 22.26524925 |
| P27824 | CALX_HUMAN Calnexin OS | 23.38640213 | 22.6495018 | 22.55510712 | 22.92092514 |
| P28066 | PSA5_HUMAN Proteasome subunit alpha type-5 OS | 22.57500839 | 22.02958298 | 21.85919952 | 22.3742733 |
| P28072 | PSB6_HUMAN Proteasome subunit beta type-6 OS | 21.40836525 | 21.35380173 | 21.3002243 | 21.63878441 |
| P28074 | PSB5_HUMAN Proteasome subunit beta type-5 OS | 20.89548683 | 20.46093941 | 20.28973961 | 20.46303749 |
| P29373 | RABP2_HUMAN Cellular retinoic acid-binding protein 2 OS | 19.99479485 | 21.08102226 | 18.70096016 | 18.61837006 |
| P29401 | TKT_HUMAN Transketolase OS | 24.20776749 | 24.15282631 | 24.09884644 | 24.58141327 |
| E9PK01 | E9PK01_HUMAN Elongation factor 1-delta (Fragment) OS | 23.50324631 | 23.40379715 | 23.96895027 | 23.96956635 |
| P29966 | MARCS_HUMAN Myristoylated alanine-rich C-kinase substrate OS | 21.71082878 | 21.66674423 | 22.72317505 | 21.75747681 |
| P30040 | ERP29_HUMAN Endoplasmic reticulum resident protein 29 OS | 20.35423279 | 20.56987381 | 20.70716667 | 20.00030899 |
| P30041 | PRDX6_HUMAN Peroxiredoxin-6 OS | 22.99870872 | 22.94952583 | 22.97891426 | 23.0866375 |
| P30044-2 | PRDX5_HUMAN Isoform Cytoplasmic+peroxisomal of Peroxiredoxin-5, mitochondrial OS | 20.39424896 | 20.23911285 | 20.7653904 | 20.62871933 |
| P30048-2 | PRDX3_HUMAN Isoform 2 of Thioredoxin-dependent peroxide reductase, mitochondrial OS | 21.37225533 | 21.13570976 | 21.50533104 | 20.64755058 |
| P30050 | RL12_HUMAN 60S ribosomal protein L12 OS | 23.00829887 | 22.51156998 | 23.46687508 | 22.59689331 |
| P30086 | PEBP1_HUMAN Phosphatidylethanolamine-binding protein 1 OS | 21.32652092 | 21.54429817 | 21.36442184 | 21.65983772 |
| P30101 | PDIA3_HUMAN Protein disulfide-isomerase A3 OS | 24.80548096 | 24.68051338 | 24.6496563 | 24.82914734 |
| P30153 | 2AAA_HUMAN Serine/threonine-protein phosphatase 2A 65 kDa regulatory subunit A alpha isoform OS | 21.11946297 | 21.01950645 | 20.6721077 | 20.49355507 |
| P31153-2 | METK2_HUMAN Isoform 2 of S-adenosylmethionine synthase isoform type-2 OS | 20.46223831 | 18.66385651 | 20.77442741 | 20.27013588 |
| P31939 | PUR9_HUMAN Bifunctional purine biosynthesis protein ATIC OS | 20.63023186 | 20.10674095 | 18.97669983 | 20.52878952 |
| P31943 | HNRH1_HUMAN Heterogeneous nuclear ribonucleoprotein H OS | 22.08086014 | 21.91002083 | 21.93419838 | 21.38184357 |
| P31946-2 | 1433B_HUMAN Isoform Short of 14-3-3 protein beta/alpha OS | 22.59208679 | 22.45312119 | 22.50772858 | 22.56241608 |
| P31948 | STIP1_HUMAN Stress-induced-phosphoprotein 1 OS | 21.73326492 | 21.64601135 | 21.98728561 | 21.84712791 |
| P32119 | PRDX2_HUMAN Peroxiredoxin-2 OS | 21.55093384 | 21.07515717 | 21.79970169 | 21.80872345 |
| D6RAN4 | D6RAN4_HUMAN 60S ribosomal protein L9 (Fragment) OS | 21.66657066 | 21.72671127 | 22.05543327 | 21.66882324 |
| P33176 | KINH_HUMAN Kinesin-1 heavy chain OS | 21.65136528 | 21.56090164 | 21.58627892 | 21.76987457 |
| H0YMM5 | H0YMM5_HUMAN Deoxyuridine 5-triphosphate nucleotidohydrolase OS | 19.21546173 | 19.30972672 | 18.38822746 | 19.26233101 |
| P34932 | HSP74_HUMAN Heat shock 70 kDa protein 4 OS | 20.9091053 | 20.69845963 | 21.35186195 | 21.7148571 |
| C9J0J7 | C9J0J7_HUMAN Profilin OS | 21.2540741 | 20.90793228 | 21.47990608 | 20.59194946 |
| G3XAM7 | G3XAM7_HUMAN Catenin (Cadherin-associated protein), alpha 1, 102kDa, isoform CRA_a OS | 19.64450836 | 19.77747917 | 18.35654449 | 19.63878632 |
| P35232 | PHB_HUMAN Prohibitin OS | 20.84597969 | 21.70281982 | 22.01991463 | 21.68589973 |
| P35241 | RADI_HUMAN Radixin OS | 19.45977974 | 18.48234367 | 19.02935028 | 19.17589569 |
| K7EP65 | K7EP65_HUMAN 60S ribosomal protein L22 (Fragment) OS | 21.96232796 | 22.31664085 | 21.82442665 | 21.80778122 |
| P35579 | MYH9_HUMAN Myosin-9 OS | 27.69298744 | 27.68772507 | 27.64972496 | 27.52260399 |
| P35580 | MYH10_HUMAN Myosin-10 OS | 19.84689903 | 20.07169342 | 20.23467636 | 19.03425407 |
| P35637-2 | FUS_HUMAN Isoform Short of RNA-binding protein FUS OS | 20.17621231 | 19.37597466 | 20.66005516 | 18.87025833 |
| C9JX88 | C9JX88_HUMAN 26S proteasome regulatory subunit 7 OS | 20.50494385 | 20.25925636 | 20.85916138 | 20.70708275 |
| P36578 | RL4_HUMAN 60S ribosomal protein L4 OS | 23.41066933 | 22.97846031 | 23.07975388 | 23.05467224 |
| P37802 | TAGL2_HUMAN Transgelin-2 OS | 23.55894279 | 23.16295433 | 23.46737289 | 23.71931458 |
| P37837 | TALDO_HUMAN Transaldolase OS | 21.08679962 | 19.02413177 | 20.32860565 | 20.90719795 |
| A0A7I2V2G2 | A0A7I2V2G2_HUMAN Stress-70 protein, mitochondrial OS | 23.15665817 | 22.81215668 | 22.90218353 | 23.21714783 |
| P39019 | RS19_HUMAN 40S ribosomal protein S19 OS | 23.29810143 | 22.96102142 | 23.22679138 | 23.2208271 |
| P39023 | RL3_HUMAN 60S ribosomal protein L3 OS | 21.65757179 | 21.20717239 | 21.90216446 | 21.84892464 |
| P39656-3 | OST48_HUMAN Isoform 3 of Dolichyl-diphosphooligosaccharide--protein glycosyltransferase 48 kDa subunit OS | 21.79780388 | 21.1774292 | 21.31942749 | 22.05870628 |
| P40227 | TCPZ_HUMAN T-complex protein 1 subunit zeta OS | 23.08307076 | 22.75735474 | 23.01816559 | 23.13899231 |
| A0A7I2V2L9 | A0A7I2V2L9_HUMAN 60S ribosomal protein L13a OS | 22.39610672 | 22.25571632 | 22.15625763 | 21.81225395 |
| P40925 | MDHC_HUMAN Malate dehydrogenase, cytoplasmic OS | 20.48000526 | 19.65826797 | 20.6313858 | 17.91763306 |
| P40926 | MDHM_HUMAN Malate dehydrogenase, mitochondrial OS | 22.90459251 | 21.84318352 | 22.86039352 | 22.60844612 |
| P40939 | ECHA_HUMAN Trifunctional enzyme subunit alpha, mitochondrial OS | 20.74032402 | 18.74769402 | 17.91740227 | 20.55496788 |
| P41091 | IF2G_HUMAN Eukaryotic translation initiation factor 2 subunit 3 OS | 20.78268814 | 20.32816696 | 19.31460571 | 18.8465004 |
| A0A6Q8PGW4 | A0A6Q8PGW4_HUMAN Diadenosine tetraphosphate synthetase OS | 22.81164742 | 22.85264397 | 22.54077911 | 22.37007523 |
| A0A0A0MSX9 | A0A0A0MSX9_HUMAN Isoleucyl-tRNA synthetase OS | 20.7786026 | 20.84766388 | 20.14631462 | 20.0578804 |
| P42677 | RS27_HUMAN 40S ribosomal protein S27 OS | 21.11457825 | 20.45813751 | 20.52238655 | 20.73297501 |
| P42704 | LPPRC_HUMAN Leucine-rich PPR motif-containing protein, mitochondrial OS | 19.63912964 | 19.58732414 | 19.86545753 | 20.08692932 |
| P42766 | RL35_HUMAN 60S ribosomal protein L35 OS | 21.38621902 | 21.34992027 | 21.19502449 | 19.02222824 |
| D6REM6 | D6REM6_HUMAN Matrin-3 OS | 20.65132141 | 20.34353638 | 20.30016899 | 20.57837677 |
| C9J3L8 | C9J3L8_HUMAN Signal sequence receptor subunit alpha OS | 20.50037956 | 18.08462524 | 19.95730591 | 18.94451141 |
| P43487-2 | RANG_HUMAN Isoform 2 of Ran-specific GTPase-activating protein OS | 20.61486053 | 21.50828362 | 19.3814888 | 19.36341476 |
| A0A0A0MR02 | A0A0A0MR02_HUMAN Outer mitochondrial membrane protein porin 2 (Fragment) OS | 21.10053062 | 18.61115074 | 21.37655449 | 20.70682907 |
| P46060 | RAGP1_HUMAN Ran GTPase-activating protein 1 OS | 21.03113365 | 21.17292023 | 21.28551292 | 21.48074532 |
| E9PLL6 | E9PLL6_HUMAN 60S ribosomal protein L27a OS | 20.7218399 | 20.5857296 | 18.10736084 | 20.9452095 |
| A0A2R8Y6J3 | A0A2R8Y6J3_HUMAN 60S ribosomal protein L5 (Fragment) OS | 21.12704277 | 20.5799427 | 21.10437584 | 21.60580254 |
| P46779 | RL28_HUMAN 60S ribosomal protein L28 OS | 19.70502472 | 20.13583374 | 19.93286705 | 20.12376213 |
| P46781 | RS9_HUMAN 40S ribosomal protein S9 OS | 22.77623558 | 22.41799927 | 22.1380558 | 22.41975021 |
| P46783 | RS10_HUMAN 40S ribosomal protein S10 OS | 22.18255997 | 22.0206604 | 22.16977501 | 22.31843376 |
| P46821 | MAP1B_HUMAN Microtubule-associated protein 1B OS | 22.97861862 | 23.17653084 | 23.35649109 | 22.91104507 |
| P46940 | IQGA1_HUMAN Ras GTPase-activating-like protein IQGAP1 OS | 24.22956657 | 24.04301643 | 24.16415215 | 24.13124084 |
| P47756-2 | CAPZB_HUMAN Isoform 2 of F-actin-capping protein subunit beta OS | 21.83491516 | 18.75108147 | 21.58765221 | 21.50455475 |
| P47914 | RL29_HUMAN 60S ribosomal protein L29 OS | 18.63410187 | 19.77980232 | 20.04072762 | 19.09104729 |
| P48047 | ATPO_HUMAN ATP synthase subunit O, mitochondrial OS | 21.42290878 | 20.24458313 | 20.49823761 | 21.00819588 |
| P48444 | COPD_HUMAN Coatomer subunit delta OS | 19.85680771 | 19.67188263 | 20.21608353 | 20.0283699 |
| P48643 | TCPE_HUMAN T-complex protein 1 subunit epsilon OS | 22.21299934 | 21.5017395 | 21.18095398 | 22.19628525 |
| P49207 | RL34_HUMAN 60S ribosomal protein L34 OS | 21.33707047 | 21.1965847 | 20.83622551 | 21.05979538 |
| P49257 | LMAN1_HUMAN Protein ERGIC-53 OS | 20.11120605 | 20.76571274 | 20.66908264 | 20.18131638 |
| P49327 | FAS_HUMAN Fatty acid synthase OS | 23.05225182 | 22.79459572 | 22.67712784 | 22.94050407 |
| P49368-2 | TCPG_HUMAN Isoform 2 of T-complex protein 1 subunit gamma OS | 23.14796066 | 22.38255501 | 22.19117165 | 22.48458481 |
| P49411 | EFTU_HUMAN Elongation factor Tu, mitochondrial OS | 21.58476639 | 21.36959457 | 21.82578468 | 21.39503479 |
| A0A6Q8PGR9 | A0A6Q8PGR9_HUMAN Alanine--tRNA ligase OS | 22.0797863 | 22.1624794 | 21.7981987 | 22.03415871 |
| P49721 | PSB2_HUMAN Proteasome subunit beta type-2 OS | 20.43440819 | 18.70707512 | 20.25418854 | 20.5731144 |
| P49748-2 | ACADV_HUMAN Isoform 2 of Very long-chain specific acyl-CoA dehydrogenase, mitochondrial OS | 21.2420826 | 20.63174248 | 17.81671143 | 20.72284126 |
| P49755 | TMEDA_HUMAN Transmembrane emp24 domain-containing protein 10 OS | 21.50949287 | 21.12905693 | 20.75066376 | 20.81464195 |
| P50395 | GDIB_HUMAN Rab GDP dissociation inhibitor beta OS | 23.33483696 | 23.04206467 | 23.48775291 | 23.14231682 |
| P50454 | SERPH_HUMAN Serpin H1 OS | 25.20258331 | 25.42825508 | 25.41969872 | 25.27831268 |
| P50502 | F10A1_HUMAN Hsc70-interacting protein OS | 21.56267357 | 20.98516655 | 21.51200294 | 20.1685524 |
| P50990 | TCPQ_HUMAN T-complex protein 1 subunit theta OS | 23.54557228 | 23.41209221 | 23.56115913 | 23.53990555 |
| P50991 | TCPD_HUMAN T-complex protein 1 subunit delta OS | 23.07407951 | 22.80142021 | 22.39876938 | 22.98285294 |
| P51148 | RAB5C_HUMAN Ras-related protein Rab-5C OS | 21.3372879 | 21.36693001 | 21.72121429 | 21.43119812 |
| P51149 | RAB7A_HUMAN Ras-related protein Rab-7a OS | 22.55817032 | 22.36644936 | 22.36946297 | 22.44447899 |
| P51571 | SSRD_HUMAN Translocon-associated protein subunit delta OS | 18.66031456 | 20.03684425 | 20.51422119 | 19.06433678 |
| P51572 | BAP31_HUMAN B-cell receptor-associated protein 31 OS | 19.22668076 | 19.28956032 | 19.14118385 | 19.75802231 |
| P52209-2 | 6PGD_HUMAN Isoform 2 of 6-phosphogluconate dehydrogenase, decarboxylating OS | 22.70541763 | 22.59386444 | 22.85699844 | 22.75509453 |
| P52272-2 | HNRPM_HUMAN Isoform 2 of Heterogeneous nuclear ribonucleoprotein M OS | 21.94966888 | 22.02789879 | 21.52257729 | 21.77916527 |
| J3KTF8 | J3KTF8_HUMAN Rho GDP-dissociation inhibitor 1 (Fragment) OS | 23.87201691 | 24.0578804 | 24.23118019 | 23.78835487 |
| P52907 | CAZA1_HUMAN F-actin-capping protein subunit alpha-1 OS | 22.37268066 | 21.3631382 | 22.77495003 | 22.74015999 |
| P53396-2 | ACLY_HUMAN Isoform 2 of ATP-citrate synthase OS | 22.4673233 | 22.21912956 | 22.16695786 | 22.45645905 |
| P53618 | COPB_HUMAN Coatomer subunit beta OS | 21.08290863 | 18.38897133 | 17.43130112 | 20.36303139 |
| P53621 | COPA_HUMAN Coatomer subunit alpha OS | 22.91932678 | 22.40603065 | 22.80846786 | 22.60731697 |
| P54136 | SYRC_HUMAN Arginine--tRNA ligase, cytoplasmic OS | 20.94877815 | 20.63156509 | 20.39100075 | 20.99748611 |
| P55060-4 | XPO2_HUMAN Isoform 4 of Exportin-2 OS | 20.3780365 | 20.3042984 | 20.11895561 | 20.2455101 |
| P55072 | TERA_HUMAN Transitional endoplasmic reticulum ATPase OS | 24.27362633 | 24.4832077 | 24.09361649 | 24.43224335 |
| P55084-2 | ECHB_HUMAN Isoform 2 of Trifunctional enzyme subunit beta, mitochondrial OS | 19.71219826 | 19.80769539 | 20.84176445 | 18.28129005 |
| H0YHC3 | H0YHC3_HUMAN Nucleosome assembly protein 1-like 1 (Fragment) OS | 20.54425049 | 20.63147545 | 20.22387886 | 21.2160244 |
| P55884 | EIF3B_HUMAN Eukaryotic translation initiation factor 3 subunit B OS | 20.8956337 | 19.67891312 | 18.57518959 | 19.43682861 |
| P59998 | ARPC4_HUMAN Actin-related protein 2/3 complex subunit 4 OS | 22.28813553 | 22.78680038 | 22.48726273 | 22.39286232 |
| P60174 | TPIS_HUMAN Triosephosphate isomerase OS | 24.44556427 | 24.71088028 | 24.43942642 | 24.62934303 |
| B3KW56 | B3KW56_HUMAN Eukaryotic translation initiation factor 3 subunit E OS | 19.85682297 | 20.29221344 | 19.07779694 | 18.93789291 |
| F8W1R7 | F8W1R7_HUMAN Myosin light polypeptide 6 OS | 24.57894135 | 24.75409508 | 24.40210533 | 24.60438919 |
| P60709 | ACTB_HUMAN Actin, cytoplasmic 1 OS | 29.33001328 | 29.86727905 | 28.66478729 | 29.2647686 |
| P60842 | IF4A1_HUMAN Eukaryotic initiation factor 4A-I OS | 24.70872498 | 24.78875351 | 25.12892342 | 24.72108841 |
| P60866 | RS20_HUMAN 40S ribosomal protein S20 OS | 22.42526627 | 22.77039909 | 22.66459656 | 22.66938591 |
| B1ALA9 | B1ALA9_HUMAN Ribose-phosphate pyrophosphokinase 1 OS | 18.88178253 | 19.33992577 | 19.43888092 | 18.15581894 |
| P60900 | PSA6_HUMAN Proteasome subunit alpha type-6 OS | 21.58536148 | 21.45156288 | 21.56369781 | 21.6724968 |
| P60953 | CDC42_HUMAN Cell division control protein 42 homolog OS | 20.10200691 | 20.73908806 | 20.56941032 | 20.01848793 |
| P60981 | DEST_HUMAN Destrin OS | 21.72313309 | 21.87286186 | 21.45256996 | 21.26384735 |
| P61019 | RAB2A_HUMAN Ras-related protein Rab-2A OS | 20.43135071 | 20.34992027 | 20.78612328 | 21.72454834 |
| P61088 | UBE2N_HUMAN Ubiquitin-conjugating enzyme E2 N OS | 21.16637611 | 21.61108208 | 21.29916382 | 21.65403366 |
| P61106 | RAB14_HUMAN Ras-related protein Rab-14 OS | 21.25638008 | 20.82010651 | 20.78420639 | 20.41565514 |
| B4DXW1 | B4DXW1_HUMAN Actin-like protein 3 OS | 22.37387466 | 21.79665565 | 22.07910347 | 22.25179291 |
| P61160 | ARP2_HUMAN Actin-related protein 2 OS | 21.78783798 | 22.01410103 | 18.80467796 | 21.74394417 |
| R4GMT0 | R4GMT0_HUMAN Alpha-centractin OS | 20.42224121 | 20.82314301 | 19.00419807 | 20.38426971 |
| P84077 | ARF1_HUMAN ADP-ribosylation factor 1 OS | 22.8700428 | 22.76237106 | 22.99137306 | 22.64961243 |
| P61224-2 | RAP1B_HUMAN Isoform 2 of Ras-related protein Rap-1b OS | 21.52367783 | 21.00374222 | 20.86944008 | 20.72275734 |
| P61247 | RS3A_HUMAN 40S ribosomal protein S3a OS | 21.48301125 | 21.54382706 | 21.85064125 | 21.55669785 |
| J3QRI7 | J3QRI7_HUMAN 60S ribosomal protein L26 (Fragment) OS | 21.39634132 | 21.09384346 | 20.63927078 | 21.47867203 |
| P61313 | RL15_HUMAN 60S ribosomal protein L15 OS | 20.59541321 | 19.92959023 | 20.48709106 | 20.49394608 |
| P61353 | RL27_HUMAN 60S ribosomal protein L27 OS | 22.97445869 | 22.77056122 | 22.63060951 | 22.8403244 |
| P61586 | RHOA_HUMAN Transforming protein RhoA OS | 19.98759651 | 20.52305603 | 20.76449966 | 20.54283524 |
| P61604 | CH10_HUMAN 10 kDa heat shock protein, mitochondrial OS | 22.4226265 | 22.30744171 | 22.5920639 | 22.50717163 |
| P61619 | S61A1_HUMAN Protein transport protein Sec61 subunit alpha isoform 1 OS | 19.67051888 | 19.80440521 | 19.67796707 | 19.7062397 |
| P61923 | COPZ1_HUMAN Coatomer subunit zeta-1 OS | 20.11540413 | 20.36559677 | 20.05814552 | 19.71450615 |
| P61970 | NTF2_HUMAN Nuclear transport factor 2 OS | 21.28427124 | 20.87530327 | 20.36581039 | 20.86135864 |
| P61978-3 | HNRPK_HUMAN Isoform 3 of Heterogeneous nuclear ribonucleoprotein K OS | 23.69338608 | 24.02622604 | 23.7654705 | 23.6595974 |
| P61981 | 1433G_HUMAN 14-3-3 protein gamma OS | 22.70366478 | 21.37623596 | 22.02516174 | 18.85562325 |
| P62081 | RS7_HUMAN 40S ribosomal protein S7 OS | 22.80146027 | 21.96789551 | 21.70977783 | 22.33535385 |
| E9PMD7 | E9PMD7_HUMAN Serine/threonine-protein phosphatase OS | 20.32025337 | 18.63227081 | 20.4649334 | 20.40405655 |
| P62191-2 | PRS4_HUMAN Isoform 2 of 26S proteasome regulatory subunit 4 OS | 19.86011696 | 20.93409157 | 20.52707291 | 20.58113861 |
| Q5JR95 | Q5JR95_HUMAN 40S ribosomal protein S8 OS | 22.29476738 | 22.55027771 | 22.76634026 | 23.05617714 |
| P62244 | RS15A_HUMAN 40S ribosomal protein S15a OS | 22.37276077 | 22.1238575 | 22.46480751 | 22.78208733 |
| P62249 | RS16_HUMAN 40S ribosomal protein S16 OS | 23.22833443 | 23.08633041 | 23.2766819 | 22.66839027 |
| P62258 | 1433E_HUMAN 14-3-3 protein epsilon OS | 24.36452866 | 24.86258888 | 25.07246971 | 24.43898201 |
| A0A2R8Y811 | A0A2R8Y811_HUMAN 40S ribosomal protein S14 (Fragment) OS | 22.77832222 | 22.81025505 | 22.53226662 | 22.7710247 |
| P62266 | RS23_HUMAN 40S ribosomal protein S23 OS | 19.91680717 | 19.80626297 | 19.22837257 | 18.28453445 |
| P62269 | RS18_HUMAN 40S ribosomal protein S18 OS | 22.22270012 | 22.41444206 | 22.35568428 | 22.2277317 |
| P62277 | RS13_HUMAN 40S ribosomal protein S13 OS | 22.7966156 | 23.0280323 | 22.61820412 | 22.66405487 |
| P62280 | RS11_HUMAN 40S ribosomal protein S11 OS | 20.74246407 | 21.16796875 | 20.88106537 | 21.46313667 |
| P62424 | RL7A_HUMAN 60S ribosomal protein L7a OS | 22.42378044 | 18.94994545 | 22.51169014 | 22.22290611 |
| P62495-2 | ERF1_HUMAN Isoform 2 of Eukaryotic peptide chain release factor subunit 1 OS | 19.04307556 | 19.20320129 | 20.02512932 | 20.0318737 |
| P62701 | RS4X_HUMAN 40S ribosomal protein S4, X isoform OS | 22.58244705 | 22.62417412 | 22.77918434 | 21.74505234 |
| P62750 | RL23A_HUMAN 60S ribosomal protein L23a OS | 22.83425903 | 22.25822067 | 22.62305832 | 22.96326447 |
| P62805 | H4_HUMAN Histone H4 OS | 18.8910141 | 18.83523941 | 18.29583359 | 18.90744781 |
| P62826 | RAN_HUMAN GTP-binding nuclear protein Ran OS | 24.46176338 | 24.08675194 | 24.00204468 | 24.21393394 |
| P62829 | RL23_HUMAN 60S ribosomal protein L23 OS | 21.83140182 | 21.14948082 | 21.43623924 | 21.33276176 |
| S4R456 | S4R456_HUMAN 40S ribosomal protein S15 (Fragment) OS | 18.87330055 | 18.98317909 | 18.99887657 | 19.39975548 |
| P62847-2 | RS24_HUMAN Isoform 2 of 40S ribosomal protein S24 OS | 20.687397 | 20.51902962 | 20.27775955 | 20.34212685 |
| P62851 | RS25_HUMAN 40S ribosomal protein S25 OS | 22.21341515 | 22.18392372 | 22.04143143 | 22.04383469 |
| P62857 | RS28_HUMAN 40S ribosomal protein S28 OS | 19.09625053 | 19.2377243 | 20.52764511 | 18.82063293 |
| E5RI99 | E5RI99_HUMAN 60S ribosomal protein L30 (Fragment) OS | 19.09628296 | 19.47548103 | 19.1880722 | 18.9431839 |
| P62899 | RL31_HUMAN 60S ribosomal protein L31 OS | 21.51287079 | 21.52960014 | 21.51566505 | 21.56034279 |
| P62906 | RL10A_HUMAN 60S ribosomal protein L10a OS | 23.07394981 | 22.79193497 | 23.26742363 | 23.41557693 |
| D3YTB1 | D3YTB1_HUMAN 60S ribosomal protein L32 (Fragment) OS | 19.64353943 | 19.51476097 | 19.31690025 | 20.50988007 |
| P62913 | RL11_HUMAN 60S ribosomal protein L11 OS | 21.7864418 | 22.02482414 | 21.53957367 | 22.34472847 |
| P62917 | RL8_HUMAN 60S ribosomal protein L8 OS | 21.62894249 | 21.89788818 | 21.69459724 | 21.11114311 |
| P62937 | PPIA_HUMAN Peptidyl-prolyl cis-trans isomerase A OS | 24.97683716 | 25.02812576 | 25.21404457 | 25.09904671 |
| P63000 | RAC1_HUMAN Ras-related C3 botulinum toxin substrate 1 OS | 21.93102837 | 21.89285851 | 21.86351585 | 22.38924217 |
| P63104 | 1433Z_HUMAN 14-3-3 protein zeta/delta OS | 24.56778717 | 24.90577698 | 24.856287 | 24.80626678 |
| P63173 | RL38_HUMAN 60S ribosomal protein L38 OS | 18.49188423 | 18.88479614 | 19.83377266 | 17.66793251 |
| I3L397 | I3L397_HUMAN Eukaryotic translation initiation factor 5A (Fragment) OS | 22.95351028 | 22.89330292 | 22.57346153 | 22.53803444 |
| P63244 | RACK1_HUMAN Receptor of activated protein C kinase 1 OS | 21.35024452 | 21.170784 | 22.76941109 | 22.2841301 |
| P67809 | YBOX1_HUMAN Y-box-binding protein 1 OS | 22.65237236 | 22.907547 | 22.40080261 | 22.9051609 |
| H0YNG3 | H0YNG3_HUMAN Signal peptidase complex catalytic subunit SEC11 OS | 19.40270424 | 19.7603302 | 19.68708801 | 19.88902283 |
| P67936 | TPM4_HUMAN Tropomyosin alpha-4 chain OS | 24.1532917 | 24.04901505 | 24.21252441 | 24.52007294 |
| P68032 | ACTC_HUMAN Actin, alpha cardiac muscle 1 OS | 25.18899345 | 24.83465576 | 24.26076317 | 25.29858971 |
| P68036-2 | UB2L3_HUMAN Isoform 2 of Ubiquitin-conjugating enzyme E2 L3 OS | 20.33745193 | 20.60421944 | 20.35863876 | 20.6382103 |
| A0A7I2V659 | A0A7I2V659_HUMAN Elongation factor 1-alpha 1 OS | 28.03396416 | 27.76287651 | 28.18669128 | 28.00961304 |
| P68363 | TBA1B_HUMAN Tubulin alpha-1B chain OS | 27.35766602 | 27.515028 | 27.62247467 | 27.29640388 |
| P68371 | TBB4B_HUMAN Tubulin beta-4B chain OS | 27.19788742 | 26.8773632 | 27.41264153 | 27.12129402 |
| P78371 | TCPB_HUMAN T-complex protein 1 subunit beta OS | 22.8313427 | 23.25364113 | 23.14559937 | 23.05617714 |
| P78417 | GSTO1_HUMAN Glutathione S-transferase omega-1 OS | 22.32882309 | 22.48089218 | 22.40794945 | 22.57431602 |
| P78527 | PRKDC_HUMAN DNA-dependent protein kinase catalytic subunit OS | 21.72587967 | 21.63214111 | 21.56630135 | 21.7286644 |
| C9JXB8 | C9JXB8_HUMAN 60S ribosomal protein L24 OS | 21.09680557 | 21.11190796 | 20.88524437 | 21.43806839 |
| J3QR09 | J3QR09_HUMAN Ribosomal protein L19 OS | 19.01748276 | 19.72578812 | 19.72573853 | 20.3035183 |
| P84103-2 | SRSF3_HUMAN Isoform 2 of Serine/arginine-rich splicing factor 3 OS | 18.7894001 | 20.36474228 | 20.44384575 | 20.59705162 |
| P98179 | RBM3_HUMAN RNA-binding protein 3 OS | 20.38131523 | 20.97477341 | 19.7901535 | 20.52735901 |
| C9JFR7 | C9JFR7_HUMAN Cytochrome c (Fragment) OS | 21.03999329 | 21.10935974 | 20.71129036 | 21.25257301 |
| F8VVM2 | F8VVM2_HUMAN Phosphate carrier protein, mitochondrial OS | 21.65123367 | 22.38389969 | 22.223526 | 21.95840454 |
| A0A024R4E5 | A0A024R4E5_HUMAN High density lipoprotein binding protein (Vigilin), isoform CRA_a OS | 22.42157364 | 22.21563911 | 21.96490288 | 22.32011604 |
| Q00610-2 | CLH1_HUMAN Isoform 2 of Clathrin heavy chain 1 OS | 24.97928238 | 24.67298126 | 24.81972694 | 24.59576607 |
| A0A1W2PPS1 | A0A1W2PPS1_HUMAN Heterogeneous nuclear ribonucleoprotein U OS | 22.69313049 | 22.85212898 | 23.09529305 | 22.65429688 |
| Q01082 | SPTB2_HUMAN Spectrin beta chain, non-erythrocytic 1 OS | 21.80770302 | 21.04853249 | 21.25919914 | 20.98607063 |
| Q01105-3 | SET_HUMAN Isoform 3 of Protein SET OS | 18.95907784 | 19.06279373 | 21.68461609 | 22.34600258 |
| Q01469 | FABP5_HUMAN Fatty acid-binding protein 5 OS | 19.00317574 | 18.85256577 | 18.67992592 | 18.67603683 |
| Q01518-2 | CAP1_HUMAN Isoform 2 of Adenylyl cyclase-associated protein 1 OS | 22.56090164 | 23.20096016 | 23.59479904 | 22.99465752 |
| Q01813 | PFKAP_HUMAN ATP-dependent 6-phosphofructokinase, platelet type OS | 22.05208588 | 21.34212685 | 21.14033508 | 21.37607574 |
| Q01995 | TAGL_HUMAN Transgelin OS | 24.7683506 | 24.65518188 | 24.50609398 | 24.88539314 |
| M0R3D6 | M0R3D6_HUMAN 60S ribosomal protein L18a (Fragment) OS | 19.92990875 | 19.86867142 | 20.1021347 | 20.40062141 |
| Q02878 | RL6_HUMAN 60S ribosomal protein L6 OS | 22.42675209 | 22.08929062 | 22.28757095 | 22.91342163 |
| Q03135 | CAV1_HUMAN Caveolin-1 OS | 22.72075462 | 22.38677025 | 22.3856926 | 22.21545982 |
| Q04637-6 | IF4G1_HUMAN Isoform E of Eukaryotic translation initiation factor 4 gamma 1 OS | 21.82915688 | 21.39131546 | 21.84586525 | 21.52061272 |
| Q04917 | 1433F_HUMAN 14-3-3 protein eta OS | 22.6102047 | 22.48047447 | 22.50006294 | 22.64180756 |
| Q05682-5 | CALD1_HUMAN Isoform 5 of Caldesmon OS | 22.96497345 | 23.71408272 | 23.85367203 | 23.31340599 |
| A0A6I8PTT9 | A0A6I8PTT9_HUMAN Glutamine--fructose-6-phosphate transaminase (isomerizing) OS | 20.48404312 | 20.22470284 | 20.57653236 | 20.77828217 |
| Q06323 | PSME1_HUMAN Proteasome activator complex subunit 1 OS | 18.18373871 | 19.07732773 | 19.4559536 | 20.34460831 |
| Q06830 | PRDX1_HUMAN Peroxiredoxin-1 OS | 25.09470558 | 24.84536743 | 24.84856987 | 25.20018959 |
| Q07020 | RL18_HUMAN 60S ribosomal protein L18 OS | 22.49071693 | 22.67646217 | 22.55506134 | 22.72008705 |
| Q07065 | CKAP4_HUMAN Cytoskeleton-associated protein 4 OS | 25.88105774 | 25.56537819 | 25.3656292 | 25.76638031 |
| Q08211 | DHX9_HUMAN ATP-dependent RNA helicase A OS | 20.88554192 | 20.41586113 | 20.59841537 | 20.43298149 |
| Q09666 | AHNK_HUMAN Neuroblast differentiation-associated protein AHNAK OS | 26.80919075 | 26.82784081 | 26.60752487 | 26.89585114 |
| Q12792 | TWF1_HUMAN Twinfilin-1 OS | 19.77402496 | 19.71676636 | 19.71513367 | 18.12091637 |
| Q13162 | PRDX4_HUMAN Peroxiredoxin-4 OS | 21.9103508 | 21.42147064 | 21.97952652 | 21.31523132 |
| Q13200 | PSMD2_HUMAN 26S proteasome non-ATPase regulatory subunit 2 OS | 20.71833229 | 20.72974396 | 20.90778542 | 20.67719269 |
| A0A7I2YQU2 | A0A7I2YQU2_HUMAN Eukaryotic translation initiation factor 3 subunit I OS | 20.01031494 | 18.65255928 | 19.58714485 | 19.62633324 |
| Q13404 | UB2V1_HUMAN Ubiquitin-conjugating enzyme E2 variant 1 OS | 25.04034424 | 25.15054321 | 24.50536728 | 24.32244301 |
| Q13509 | TBB3_HUMAN Tubulin beta-3 chain OS | 20.62729454 | 21.03704643 | 20.46293831 | 20.93919373 |
| Q13813-3 | SPTN1_HUMAN Isoform 3 of Spectrin alpha chain, non-erythrocytic 1 OS | 22.29094887 | 22.0790062 | 22.85004997 | 21.93340683 |
| Q13885 | TBB2A_HUMAN Tubulin beta-2A chain OS | 20.257761 | 20.23139954 | 20.50067139 | 18.76807785 |
| Q14019 | COTL1_HUMAN Coactosin-like protein OS | 21.47476768 | 21.88110352 | 21.42188263 | 21.59609604 |
| H0YA96 | H0YA96_HUMAN Heterogeneous nuclear ribonucleoprotein D0 (Fragment) OS | 21.51460648 | 21.17053795 | 21.23800659 | 21.82178116 |
| Q14152 | EIF3A_HUMAN Eukaryotic translation initiation factor 3 subunit A OS | 21.46453285 | 21.34044266 | 21.08679962 | 21.03402519 |
| Q14203-5 | DCTN1_HUMAN Isoform 5 of Dynactin subunit 1 OS | 19.51335335 | 19.29692459 | 19.15960503 | 19.35968971 |
| Q14204 | DYHC1_HUMAN Cytoplasmic dynein 1 heavy chain 1 OS | 24.34435081 | 24.31035805 | 24.19730568 | 24.18714523 |
| Q14315 | FLNC_HUMAN Filamin-C OS | 27.12149239 | 27.11723709 | 27.25349617 | 27.0048542 |
| Q14697 | GANAB_HUMAN Neutral alpha-glucosidase AB OS | 23.36292458 | 22.8890934 | 22.88393974 | 23.58060837 |
| Q14764 | MVP_HUMAN Major vault protein OS | 22.82702637 | 22.94881439 | 22.78640175 | 23.10726929 |
| Q14847 | LASP1_HUMAN LIM and SH3 domain protein 1 OS | 21.25678253 | 22.18474007 | 21.33788681 | 21.2075882 |
| Q14974 | IMB1_HUMAN Importin subunit beta-1 OS | 22.3130188 | 22.37877655 | 22.22552681 | 22.31893158 |
| Q15019 | SEPT2_HUMAN Septin-2 OS | 21.82667732 | 21.78524399 | 20.0318737 | 21.30557823 |
| Q15056-2 | IF4H_HUMAN Isoform Short of Eukaryotic translation initiation factor 4H OS | 21.22146225 | 21.41622162 | 21.5228653 | 22.01229477 |
| Q15084-3 | PDIA6_HUMAN Isoform 3 of Protein disulfide-isomerase A6 OS | 23.18219757 | 23.13641357 | 23.43948936 | 23.43109512 |
| Q15149-4 | PLEC_HUMAN Isoform 4 of Plectin OS | 25.25093269 | 24.91988945 | 25.1126709 | 25.1349659 |
| Q15293 | RCN1_HUMAN Reticulocalbin-1 OS | 20.77378273 | 21.71234131 | 17.98793602 | 21.14108467 |
| Q15365 | PCBP1_HUMAN Poly(rC)-binding protein 1 OS | 21.73677826 | 21.9072361 | 21.34472847 | 21.80565643 |
| Q15366-6 | PCBP2_HUMAN Isoform 6 of Poly(rC)-binding protein 2 OS | 20.30106354 | 20.06736565 | 20.74131203 | 20.65499687 |
| B8ZZU8 | B8ZZU8_HUMAN Elongin-B OS | 18.43782806 | 18.92963982 | 19.85376549 | 20.35638237 |
| F5H365 | F5H365_HUMAN Protein transport protein SEC23 OS | 21.41926003 | 21.0653286 | 20.91568375 | 21.73454666 |
| Q15691 | MARE1_HUMAN Microtubule-associated protein RP/EB family member 1 OS | 18.58977509 | 19.15172577 | 19.41030312 | 18.99092293 |
| Q15758 | AAAT_HUMAN Neutral amino acid transporter B(0) OS | 20.91210556 | 21.30374146 | 21.05629349 | 21.60082245 |
| Q15907 | RB11B_HUMAN Ras-related protein Rab-11B OS | 22.33606339 | 22.42513847 | 22.12543488 | 22.69670105 |
| Q15942 | ZYX_HUMAN Zyxin OS | 22.87152863 | 22.36383247 | 22.08916283 | 22.49947929 |
| E7ES33 | E7ES33_HUMAN Septin OS | 21.02823448 | 21.39937019 | 21.57740784 | 20.84544373 |
| Q16222-3 | UAP1_HUMAN Isoform 3 of UDP-N-acetylhexosamine pyrophosphorylase OS | 20.71774673 | 20.5890255 | 21.29092026 | 20.81362534 |
| Q16527 | CSRP2_HUMAN Cysteine and glycine-rich protein 2 OS | 20.24898911 | 19.03178215 | 20.2585659 | 20.15177536 |
| Q16555-2 | DPYL2_HUMAN Isoform 2 of Dihydropyrimidinase-related protein 2 OS | 22.89191437 | 22.48387146 | 22.51535225 | 22.79141808 |
| Q16658 | FSCN1_HUMAN Fascin OS | 23.12012863 | 23.95774841 | 23.68880463 | 23.29151154 |
| A0A7I2YQ74 | A0A7I2YQ74_HUMAN UTP--glucose-1-phosphate uridylyltransferase OS | 20.73040771 | 20.23572922 | 20.22599792 | 20.52821732 |
| E9PIR7 | E9PIR7_HUMAN Thioredoxin-disulfide reductase OS | 20.13520813 | 22.20421982 | 20.66031647 | 20.95347404 |
| Q6NZI2 | CAVN1_HUMAN Caveolae-associated protein 1 OS | 23.55027771 | 23.35231972 | 23.77846336 | 23.37583733 |
| Q6UVK1 | CSPG4_HUMAN Chondroitin sulfate proteoglycan 4 OS | 22.93912315 | 22.35345078 | 22.73419571 | 22.38031006 |
| Q70UQ0-4 | IKIP_HUMAN Isoform 4 of Inhibitor of nuclear factor kappa-B kinase-interacting protein OS | 20.96169281 | 20.2250576 | 19.98814011 | 20.65970612 |
| Q7KZF4 | SND1_HUMAN Staphylococcal nuclease domain-containing protein 1 OS | 23.31686211 | 23.32649422 | 23.29291534 | 23.51544952 |
| Q86VP6 | CAND1_HUMAN Cullin-associated NEDD8-dissociated protein 1 OS | 20.88084221 | 20.96027946 | 20.43338966 | 20.77313995 |
| Q8NBS9-2 | TXND5_HUMAN Isoform 2 of Thioredoxin domain-containing protein 5 OS | 20.94105911 | 20.64702225 | 21.04286766 | 20.72916222 |
| Q8TED1 | GPX8_HUMAN Probable glutathione peroxidase 8 OS | 18.95015335 | 20.26796532 | 20.07535362 | 20.22776222 |
| Q8WUM4 | PDC6I_HUMAN Programmed cell death 6-interacting protein OS | 21.52750206 | 21.55651093 | 21.44924736 | 20.98523521 |
| Q92499-3 | DDX1_HUMAN Isoform 3 of ATP-dependent RNA helicase DDX1 OS | 21.01780891 | 20.79344368 | 20.19544411 | 20.9557457 |
| Q92598-2 | HS105_HUMAN Isoform Beta of Heat shock protein 105 kDa OS | 20.23642921 | 20.25234222 | 20.68208885 | 19.83480835 |
| Q92616 | GCN1_HUMAN eIF-2-alpha kinase activator GCN1 OS | 19.96268082 | 19.60320663 | 19.48883629 | 18.87059975 |
| A0A087WTP3 | A0A087WTP3_HUMAN Far upstream element-binding protein 2 OS | 21.19490433 | 21.02018547 | 21.56917953 | 21.09732056 |
| Q92973-2 | TNPO1_HUMAN Isoform 2 of Transportin-1 OS | 20.04566956 | 20.53402519 | 18.87665558 | 20.35315514 |
| Q969G5 | CAVN3_HUMAN Caveolae-associated protein 3 OS | 21.82442665 | 20.65438461 | 21.56267357 | 21.22334862 |
| Q969H8 | MYDGF_HUMAN Myeloid-derived growth factor OS | 21.44091034 | 21.9212532 | 21.48394585 | 21.342453 |
| Q96AE4 | FUBP1_HUMAN Far upstream element-binding protein 1 OS | 20.93272209 | 20.83684158 | 20.31506538 | 21.13345337 |
| Q96AG4 | LRC59_HUMAN Leucine-rich repeat-containing protein 59 OS | 22.75144196 | 21.95095062 | 22.62464333 | 22.54898453 |
| Q96AY3 | FKB10_HUMAN Peptidyl-prolyl cis-trans isomerase FKBP10 OS | 20.19568443 | 20.47447014 | 20.399683 | 20.26132393 |
| Q96HC4 | PDLI5_HUMAN PDZ and LIM domain protein 5 OS | 19.94792175 | 19.39408112 | 19.55939865 | 19.54916763 |
| Q96QK1 | VPS35_HUMAN Vacuolar protein sorting-associated protein 35 OS | 20.62987518 | 20.65831184 | 20.38900566 | 21.01726532 |
| Q96TA1-2 | NIBA2_HUMAN Isoform 2 of Protein Niban 2 OS | 21.71686745 | 20.96402168 | 20.68157387 | 21.38052177 |
| A0A087X271 | A0A087X271_HUMAN Calponin (Fragment) OS | 23.08953476 | 22.62776184 | 22.79120064 | 22.18797493 |
| A0A7I2V641 | A0A7I2V641_HUMAN 26S proteasome non-ATPase regulatory subunit 1 OS | 20.179739 | 19.58616829 | 19.29665565 | 20.6661377 |
| Q99497 | PARK7_HUMAN Parkinson disease protein 7 OS | 22.87517166 | 22.71793556 | 22.83803558 | 22.54533577 |
| Q99536 | VAT1_HUMAN Synaptic vesicle membrane protein VAT-1 homolog OS | 21.73098755 | 21.59368324 | 22.00295448 | 21.65678596 |
| F5GY37 | F5GY37_HUMAN Prohibitin OS | 21.46203804 | 18.62091637 | 21.73525047 | 21.64891052 |
| Q99715-4 | COCA1_HUMAN Isoform 4 of Collagen alpha-1(XII) chain OS | 23.96322823 | 24.28029633 | 23.71826935 | 23.67012024 |
| Q99832 | TCPH_HUMAN T-complex protein 1 subunit eta OS | 22.27301407 | 22.31215858 | 21.68286133 | 22.31942749 |
| Q9BRA2 | TXD17_HUMAN Thioredoxin domain-containing protein 17 OS | 20.04393578 | 20.12754631 | 19.92491722 | 20.79519081 |
| Q9BSJ8 | ESYT1_HUMAN Extended synaptotagmin-1 OS | 21.77591324 | 21.3288784 | 21.09029388 | 21.22605705 |
| Q9BUF5 | TBB6_HUMAN Tubulin beta-6 chain OS | 22.85214806 | 22.76218987 | 22.93560219 | 22.94660187 |
| Q9BVK6 | TMED9_HUMAN Transmembrane emp24 domain-containing protein 9 OS | 20.93358612 | 20.93574715 | 21.05231857 | 21.12647438 |
| Q9H0U4 | RAB1B_HUMAN Ras-related protein Rab-1B OS | 22.43270111 | 22.57503319 | 22.97305679 | 22.72486115 |
| Q5T123 | Q5T123_HUMAN SH3 domain-binding glutamic acid-rich-like protein 3 OS | 20.5874691 | 20.44748306 | 20.63484573 | 20.56458092 |
| Q9H3N1 | TMX1_HUMAN Thioredoxin-related transmembrane protein 1 OS | 20.38553429 | 20.24435043 | 19.96592522 | 18.50144386 |
| Q9H4M9 | EHD1_HUMAN EH domain-containing protein 1 OS | 21.21626091 | 21.05609512 | 21.66209984 | 21.61800194 |
| Q9HB71 | CYBP_HUMAN Calcyclin-binding protein OS | 21.07012177 | 21.29193306 | 21.10667801 | 21.24196625 |
| A0A087X163 | A0A087X163_HUMAN Ras-related protein Rab-18 OS | 19.9343071 | 19.69951057 | 18.67591667 | 19.63078308 |
| Q9NQC3-2 | RTN4_HUMAN Isoform B of Reticulon-4 OS | 22.88684464 | 22.36828995 | 23.12352562 | 22.69799423 |
| Q9NR12 | PDLI7_HUMAN PDZ and LIM domain protein 7 OS | 19.44159889 | 19.90405464 | 20.33527184 | 20.15003967 |
| Q9NRV9 | HEBP1_HUMAN Heme-binding protein 1 OS | 20.5460434 | 20.23923111 | 19.58080673 | 18.86299706 |
| D6RGI3 | D6RGI3_HUMAN Septin OS | 22.09519577 | 21.59099007 | 22.53848457 | 21.22364426 |
| Q9NZM1-6 | MYOF_HUMAN Isoform 6 of Myoferlin OS | 23.33456421 | 23.35930824 | 23.51941299 | 23.40561485 |
| Q9NZN4 | EHD2_HUMAN EH domain-containing protein 2 OS | 21.6853447 | 21.87582779 | 22.1516819 | 21.82842255 |
| Q9P0L0 | VAPA_HUMAN Vesicle-associated membrane protein-associated protein A OS | 20.19111252 | 20.65377235 | 21.08173752 | 19.89276123 |
| Q9P2E9 | RRBP1_HUMAN Ribosome-binding protein 1 OS | 23.14600372 | 23.28489113 | 23.42598343 | 23.22552681 |
| F8VQE1 | F8VQE1_HUMAN LIM domain and actin-binding protein 1 OS | 20.20669556 | 20.37708282 | 20.42419052 | 20.58884239 |
| Q9UHD8-7 | SEPT9_HUMAN Isoform 7 of Septin-9 OS | 22.22393799 | 22.41959572 | 22.67260361 | 22.53791618 |
| Q9ULV4 | COR1C_HUMAN Coronin-1C OS | 20.28059387 | 21.59382057 | 20.74935532 | 20.60855865 |
| Q9UQ80-2 | PA2G4_HUMAN Isoform 2 of Proliferation-associated protein 2G4 OS | 21.73144341 | 21.54198456 | 22.05075836 | 21.95191002 |
| Q9Y230 | RUVB2_HUMAN RuvB-like 2 OS | 20.31572914 | 20.23186874 | 20.65342331 | 20.00360489 |
| Q9Y265 | RUVB1_HUMAN RuvB-like 1 OS | 20.3109684 | 20.10174942 | 19.86678696 | 19.98036194 |
| Q9Y266 | NUDC_HUMAN Nuclear migration protein nudC OS | 19.5786705 | 20.15721321 | 19.76089859 | 19.83787346 |
| Q9Y3U8 | RL36_HUMAN 60S ribosomal protein L36 OS | 20.25614929 | 20.49882126 | 20.18652534 | 18.73384285 |
| Q9Y490 | TLN1_HUMAN Talin-1 OS | 24.98315811 | 25.01260948 | 25.03216743 | 24.93251419 |
| A0A087X054 | A0A087X054_HUMAN Hypoxia up-regulated protein 1 OS | 21.84796906 | 21.17876625 | 20.70235443 | 21.04979515 |
| Q9Y617-2 | SERC_HUMAN Isoform 2 of Phosphoserine aminotransferase OS | 21.61265755 | 21.64315224 | 21.30635834 | 21.52750206 |
| Q9Y678 | COPG1_HUMAN Coatomer subunit gamma-1 OS | 22.16438293 | 21.94998932 | 21.65744019 | 22.20573997 |
| Q9Y696 | CLIC4_HUMAN Chloride intracellular channel protein 4 OS | 22.25942802 | 22.63710403 | 22.63955688 | 22.24251938 |
