# Supplementary material for: SARS-CoV-2 infection causes periodontal fibrotic pathogenesis through deregulating mitochondrial beta-oxidation
Source: Cell Death Discov. 2023 May 26;9:175. doi: 10.1038/s41420-023-01474-2 (PMC10214333; doi:10.1038/s41420-023-01474-2)
Supplement: Supplementary file 10 — Appendix Table 2 [file 41420_2023_1474_MOESM10_ESM.docx]

**Appendix Table 2**

The antibodies, primers, protein and viruses used in the study.

| **Primary antibodies (Immunofluorescence)** | | | | | | |
| --- | --- | --- | --- | --- | --- | --- |
| **Antigen** | **Figure** | **Final working concentration**  **(μg/ml)** | **Company** | **Cat. No.** | **Batch No.** |  |
| ACE2 | 1B, 1C | 5 | Proteintech | 66699-1-Ig | - |  |
| APC His-Tag conjugated antibody | 1E, 1F | 10 μl/10^6 cells | R&D | IC050A | ABUH0521061 |  |
| Collagen I | 3I, 5H | 5 | RockLand | 600-401-103 | 46588 |  |
| MMP1 | 3K | 5 | Genetex | GTX24043 | 18912 |  |
| TMPRSS2 | 1B, 1C | 8.86 | Abcam | ab109131 | GR3343890-9 |  |

| **Secondary antibodies (Immunofluorescence)** | | | | |
| --- | --- | --- | --- | --- |
| **Target** | **Final working concentration**  **(μg/ml)** | **Company** | **Cat. No.** | **Batch No.** |
| Alexa 488 donkey anti-rabbit IgG | 6.67 | Life Technologies | A21206 | 2072687 |
| Alexa 568 donkey anti-mouse IgG | 4 | Life Technologies | A10037 | 1303018 |
| Alexa 568 donkey anti-rabbit IgG | 4 | Life Technologies | A10042 | 1964370 |

| **Antibody (FACS)** | | | | | |
| --- | --- | --- | --- | --- | --- |
| **Protein** | **Figure** | **Final working concentration**  **(μg/ml)** | **Company** | **Cat. No.** | **Batch No.** |
| APC His-Tag conjugated antibody | 1G, 1H | 10 μl/10^6 cells | R&D | IC050A | ABUH0521061 |

| **Primary antibodies (Western Blotting)** | | | | | |
| --- | --- | --- | --- | --- | --- |
| **Protein** | **Figure** | **Final working concentration**  **(μg/ml)** | **Company** | **Cat. No.** | **Batch No.** |
| ACE2 | 1D | 1 | Proteintech | 66699-1-Ig | - |
| Collagen I | 3A, 3D, 5F | 2 | RockLand | 600-401-103 | 46588 |
| Lamin B1 | 1D, 5F | 1 | Abcam | ab16048 | GR3417466-1 |
| MMP1 | 3A, 3D | 2 | Genetex | GTX24043 | 18912 |
| GAPDH | 3A, 3D | 0.2 | Santa Cruz | SC-32233 | H2114 |
| TMPRSS2 | 1D | 1.77 | Abcam | ab109131 | GR3343890-9 |

| **Secondary antibodies (Western Blotting)** | | | | |
| --- | --- | --- | --- | --- |
| **Target** | **Final working concentration**  **(μg/ml)** | **Company** | **Cat. No.** | **Batch No.** |
| HRP mouse | 0.184 | Cell Signalling | 7076 | 33 |
| HRP rabbit | 0.06 | Cell Signalling | 7074 | 28 |

| **Primers** | | | |
| --- | --- | --- | --- |
| **Gene**  **(human)** | **Forward primer ('5-'3)** | **Reverse primer ('5-'3)** | **PCR**  **Product size** |
| COL1A1 | GTGCTAAAGGTGCCAATGGT | ACCAGGTTCACCGCTGTTAC | 128 |
| GapDH | ATCACTGCCACCCAGAAGAC | CAGTGAGCTTCCCGTTCAG | 148 |
| MMP1 | TGCTCATGCTTTTCAACCAG | AGTTCATGAGCTGCAACACG | 117 |
| 36b4 | GCAATGTTGCCAGTGTCTGT | GCCTTGACCTTTTCAGCAAG | 142 |

| **Recombinant Protein** | | |
| --- | --- | --- |
| **Protein** | **Company** | **Cat. No.** |
| SARS-CoV-2 Spike His tag Protein | R&D | 10549-CV-100 |

| **Plasmids** | | |
| --- | --- | --- |
| **Vector** | **Company** | **Cat. No.** |
| pLVX-EF1alpha-eGFP-2xStrep-IRES-Puro | Addgene | 141395 |
| pLVX-EF1alpha-SARS-CoV-2-E-2xStrep-IRES-Puro | Addgene | 141385 |
| pLVX-EF1alpha-SARS-CoV-2-M-2xStrep-IRES-Puro | Addgene | 141386 |
| pLVX-EF1alpha-SARS-CoV-2-N-2xStrep-IRES-Puro | Addgene | 141391 |
| pWPI-IRES-Puro-Ak | Addgene | 154984 |
| pWPI-IRES-Puro-Ak-ACE2 | Addgene | 154985 |

| **Mitochondrial inhibitor** | **Company** | **Cat. No.** |
| --- | --- | --- |
| Etomoxir | Sigma | 236020 |
